# Uncovering outer-sphere mechanisms governing chemoselectivity in copper-photocatalyzed ATRA reactions of CF_3_SO_2_Cl with alkenes

**DOI:** 10.1039/d5sc06553d

**Published:** 2025-12-10

**Authors:** Farshad Shiri, Morteza Jamshidi, Saba Hadidi, Robert Stranger, Alireza Ariafard

**Affiliations:** a Department of Chemistry, The Hong Kong University of Science and Technology Kowloon Hong Kong 999077 P. R. China; b Research School of Chemistry, Australian National University Canberra Australian Capital Territory 2601 Australia alireza.ariafard@utas.edu.au; c Inorganic Chemistry Department, Faculty of Chemistry, Razi University Kermanshah 6714414971 Iran s.hadidi@razi.ac.ir

## Abstract

This work presents a detailed DFT-based mechanistic investigation of copper-photocatalyzed atom transfer radical addition (ATRA) reactions between CF_3_SO_2_Cl and alkenes. Depending on the electronic nature of the alkene substrate, these reactions yield either RCl or RSO_2_Cl products. The unusual divergence in product selectivity has led to the proposal of multiple mechanistic pathways. In this study, we show that all productive pathways proceed exclusively *via* outer-sphere single-electron transfer and identify two previously unrecognized mechanisms: an S(vi)/S(iv) redox cycling mechanism responsible for RSO_2_Cl formation, and a 2c–3e Cl-coordination-induced SET mechanism accounting for RCl formation. These two pathways represent the first models to explicitly demonstrate the bifunctional role of the [SO_2_Cl]^−^ anion in governing divergent product formation. Additionally, we identify a third, cationic mechanism, in which the carbon-centred radical is oxidized to a carbocation by Cu(ii), competing with the other pathways and likewise leading to RCl. Taken together, these results provide a useful framework for understanding chemoselectivity in this class of photocatalytic transformations and may help guide the design of future ATRA protocols.

## Abstract 2

Cu-photocatalyzed ATRA of CF_3_SO_2_Cl with alkenes proceed *via* outer-sphere SET pathways. Three distinct mechanisms, S(vi)/S(iv) redox cycling, 2c–3e Cl-induced SET, and a cationic route, account for the divergent RSO_2_Cl and RCl formation.

## Graphical abstract

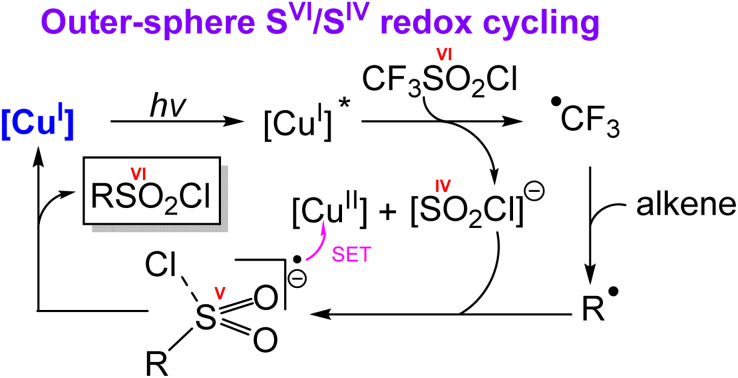

## Introduction

CF_3_-containing compounds are highly significant for their ability to enhance drug efficacy by increasing lipophilicity, improving bioactivity, and providing greater metabolic stability.^1,2^ These properties make them indispensable in pharmaceuticals, agrochemicals, and advanced material development.^3^ Consequently, many researchers have focused on developing methods to install a CF_3_ group onto organic frameworks.^4–32^ In this context, one effective approach involves incorporating a CF_3_ group into unsaturated C–C bonds, using methods such as transition metal catalysis (*e.g.*, thio-trifluoromethylation of alkenes) and photoredox catalysis (*e.g.*, cyclopropanation of alkynes).^33–38^

**Fig. 1:**
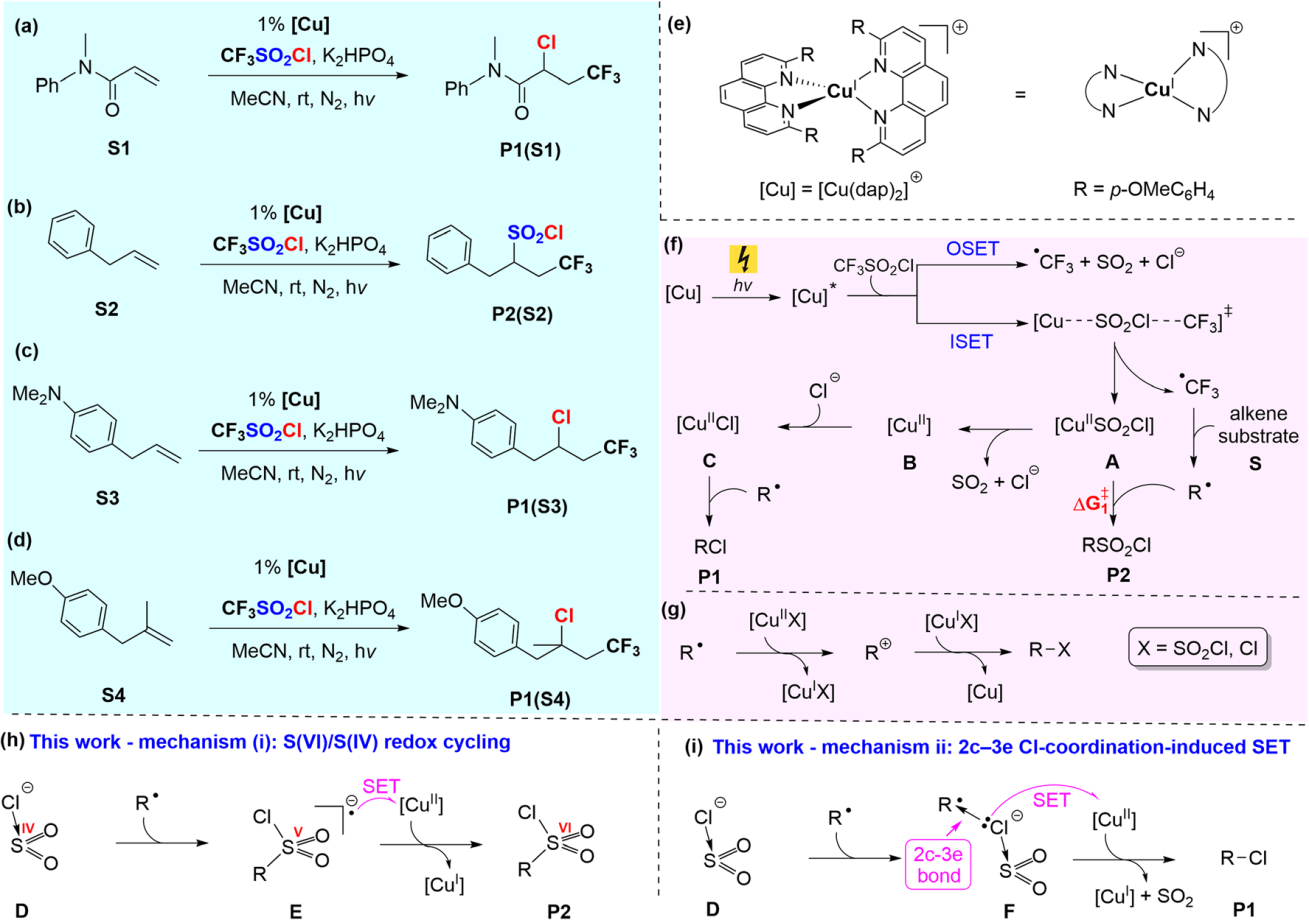
(a–d) Experimentally observed product selectivity in copper-photocatalyzed ATRA reactions of CF_3_SO_2_Cl with four representative alkene substrates S1–S4. (e) Structure of the photocatalyst [Cu(dap)_2_]Cl. (f) Previously proposed mechanism by Pham *et al.*, featuring inner-sphere (ISET) and outer-sphere (OSET) SET pathways. (g) Their evaluation of a cationic mechanism involving five-coordinate Cu(ii) complexes as oxidants. (h) Mechanism (i) proposed in this work: an S(vi)/S(iv) redox cycling pathway responsible for RSO_2_Cl formation. (i) Mechanism (ii) proposed in this work: a 2c–3e Cl-coordination-induced outer-sphere SET mechanism accounting for RCl formation.

Among photoredox catalysis methods, the copper photocatalyzed atom-transfer radical addition (ATRA) reactions of CF_3_SO_2_Cl (triflyl chloride) with alkenes, independently developed by Dolbier *et al.*^39^ and Reiser *et al.*,^40^ stand out as groundbreaking and original discoveries in the field (Fig. 1a–d). Dolbier *et al.* reported that the irradiation of electron-deficient alkenes, such as substrate S1, and triflyl chloride in the presence of a [Cu(dap)_2_]Cl catalyst (Fig. 1e) results in trifluoromethylchlorination with the extrusion of SO_2_, yielding P1(S1) as the sole product (Fig. 1a).^39^ In contrast, Reiser *et al.* observed that the irradiation of electron-neutral alkenes, such as substrate S2, and triflyl chloride, catalyzed by [Cu(dap)_2_]Cl, results in trifluoromethylchlorosulfonylation without SO_2_ extrusion, yielding P2(S2) as the major product (Fig. 1b). However, Reiser *et al.* observed that the product distribution is highly sensitive to the electronic nature of alkenes. For example, replacing the phenyl ring in substrate S2 with a *para*-aminophenyl group to form substrate S3 led to the selective formation of product P1 (Fig. 1c). Similarly, substrate S4, characterized by a disubstituted alkene at the C1 position, exclusively yielded product P1 (Fig. 1d).^40^

Building upon the pioneering work of Dolbier *et al.* and Reiser *et al.*, numerous studies have advanced this specific light-driven transformation, catalysed not only by copper complexes but also activated by alternative systems, leading to significant breakthroughs in synthetic methodology.^41–67^ However, despite substantial progress on the synthetic side, the mechanistic understanding of this process remains limited.

Various reaction mechanisms, based on both computational^68^ and experimental^39,40,69–72^ findings, have been proposed to explain the strikingly divergent product outcomes that arise from subtle changes in alkene structure. During the preparation of this work, a related computational study by Pham *et al.*^68^ was published. While their study offers valuable insights into this class of reactions, our independent investigation supports a different mechanistic picture, which is explored in detail herein.

In the following section, we briefly review the mechanism proposed by Pham *et al.*^68^ as background to our investigation. In their study, the reaction is proposed to initiate with photoexcitation of the Cu(i) complex [Cu(dap)_2_]^+^, followed by single-electron transfer (SET) to triflyl chloride *via* either an inner-sphere (ISET) or outer-sphere (OSET) pathway, as illustrated in Fig. 1f. Among these, the inner-sphere mechanism was reported to be more favourable, generating a ˙CF_3_ radical and leaving a [SO_2_Cl]^−^ anion coordinated to the *in situ* generated Cu(ii) atom (intermediate A in Fig. 1f). The ˙CF_3_ radical subsequently reacts with the alkene substrate S to form the R˙ radical. They also proposed that a ligand exchange between a Cl^−^ anion and the [SO_2_Cl]^−^ ligand in intermediate A, yielding the more stable intermediate C. The R˙ radical can subsequently react *via* two distinct pathways: (a) with the [SO_2_Cl]^−^ ligand in intermediate A, leading to the formation of product P2 (RSO_2_Cl), or (b) with the Cl ligand in intermediate C, resulting in the formation of product P1 (RCl). The product distribution has been proposed to depend on the 
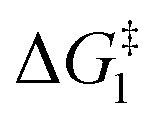
 value (Fig. 1f). A relatively small 
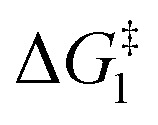
 favours the formation of product P2, whereas a higher 
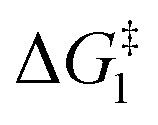
 enables the ligand exchange process to occur, leading to the formation of intermediate C, which subsequently reacts with the R˙ radical to afford product P1. However, this conclusion was reached without calculating the transition structures connecting A to B and B to C.

To evaluate the accuracy of this claim, we successfully located the crucial transition structures in this study and found that A connects to B through a remarkably low activation barrier of only 1.7 kcal mol^−1^ (*vide infra*, Fig. 5). Consequently, the conversion of A to B occurs significantly faster, effectively outcompeting the reaction of R˙ with A. It follows that if the reaction were to proceed *via* the inner-sphere mechanism proposed by Pham *et al.*,^68^ product P1 would be formed exclusively, regardless of the alkene substrate. This outcome does not align with the full set of experimental findings, suggesting that additional, previously unrecognised mechanisms may be needed to account for the observed product distributions.

Our efforts to identify new pathways for the formation of P1 and P2 have led to the discovery of two unprecedented mechanisms. We will show in this study that in these two mechanisms, the [SO_2_Cl]^−^ anion, generated *in situ via* outer sphere electron transfer from the excited Cu complex to CF_3_SO_2_Cl, plays a pivotal role as the key intermediate. This anion can interact with the R˙ radical through two distinct pathways:

Mechanism (i) (S(vi)/S(iv) redox cycling, Fig. 1h): The sulfur atom of [SO_2_Cl]^−^ binds to R˙, oxidizing sulfur from a formal oxidation state of +4 to +5 and forming intermediate E. This highly reactive intermediate then transfers an electron to the Cu(ii) atom *via* an outer-sphere mechanism, leading to the formation of product P2 and regenerating the Cu(i) catalyst.

Mechanism (ii) (2c–3e Cl-coordination-induced SET, Fig. 1i): The [SO_2_Cl]^−^ anion binds to R˙ *via* its Cl atom, forming a 2c–3e bond in intermediate F. This intermediate subsequently transfers an electron to the Cu(ii) atom through an outer-sphere mechanism, yielding product P1, accompanied by the release of SO_2_ and the regeneration of the Cu(i) catalyst.

The formation of a carbocation through outer-sphere electron transfer from R˙ to a Cu(ii) complex, followed by its trapping by an available anion, represents another plausible pathway for generating the desired products (mechanism (iii)). Pham *et al.*^68^ investigated this pathway using five-coordinate Cu(ii) complexes, [Cu^II^Cl] and [Cu^II^SO_2_Cl], as oxidants and concluded that it was unfavourable, ruling out its viability (Fig. 1g). However, in this study, we demonstrate that five-coordinate Cu(ii) complexes are significantly weaker oxidants compared to the four-coordinate Cu(ii) complex [Cu^II^]. As a result, the formation of carbocations becomes energetically much more favourable when the more potent four-coordinate Cu(ii) complex [Cu^II^] serves as the oxidant. In this study, we assert that this carbocationic pathway is indeed the operative mechanism when substrates can generate very stable carbocations.

As mentioned above, it was concluded by Pham *et al.* that the electron transfer between the photoexcited complex [Cu(dap)_2_]^+^* and CF_3_SO_2_Cl proceeds *via* an inner-sphere mechanism.^68^ In contrast, we demonstrate here that this is not the case. Once CF_3_SO_2_Cl and [Cu(dap)_2_]^+^* form an outer-sphere complex (the initial step of an inner-sphere mechanism), an electron is immediately transferred from [Cu(dap)_2_]^+^* to CF_3_SO_2_Cl with an activation energy as low as 0.2 kcal mol^−1^ (*vide infra*, Fig. 3c). This result suggests that an inner-sphere mechanism for electron transfer is unlikely. Here, we will demonstrate that all processes, including the electron transfer from [Cu(dap)_2_]^+^* to CF_3_SO_2_Cl and the formation of products P1 and P2, occur exclusively through outer-sphere mechanisms.

## Results and discussion

### Photoexcitation of [Cu(dap)_2_]^+^ and the outer-sphere electron transfer to CF_3_SO_2_Cl

**Fig. 2:**
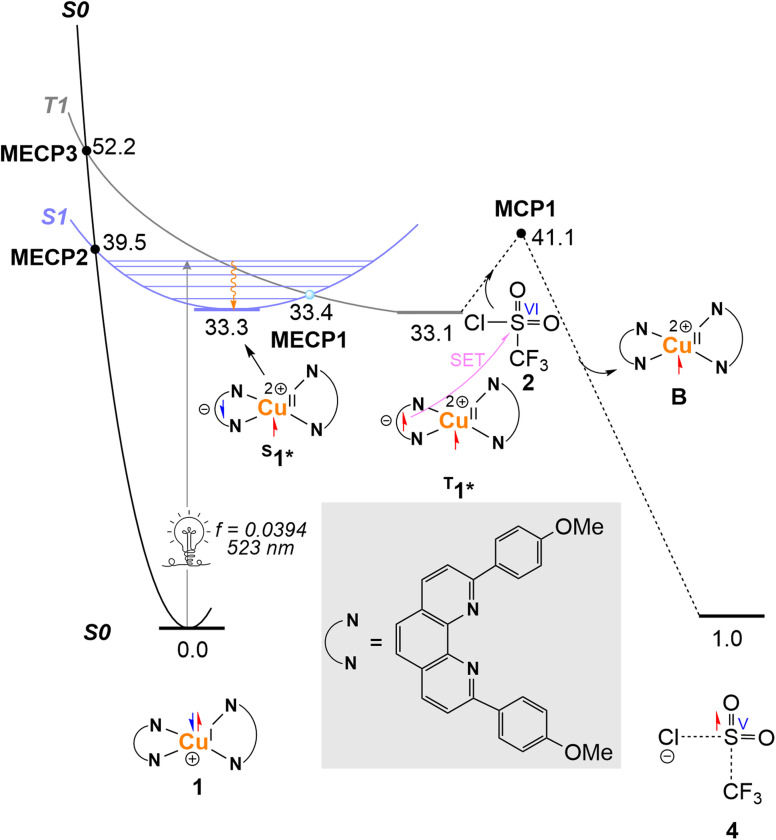
Calculated Jablonski-type energy diagram and free energy profile for the light-driven outer-sphere electron transfer (SET) mechanism between [Cu(dap)_2_]^+^ and CF_3_SO_2_Cl. Relative energies are given in kcal mol^−1^.

As proposed in the literature, the copper-photocatalyzed ATRA reaction of triflyl chloride with alkenes begins with the photoexcitation of [Cu(dap)_2_]^+^.^69–76^ This excitation promotes an electron from a fully occupied Cu d orbital to a π* orbital on the dap ligands, thereby oxidizing the Cu centre from Cu(i) to Cu(ii). This metal-to-ligand charge transfer (MLCT) generates complex ^S^1* on the S_1_ excited-state singlet surface (Fig. 2).

From S1*, we considered two possible deactivation pathways: (i) intersystem crossing (ISC) to the slightly more stable triplet-state complex ^T^1*via the minimum energy crossing point (MECP) MECP1 and (ii) non-radiative relaxation back to the ground-state complex 1*via*MECP2. MECP1 is calculated to be 6.1 kcal mol^−1^ lower in energy than MECP2, indicating that ISC to ^T^1* is energetically favoured and that ^T^1* should be readily populated upon photoexcitation of 1.

From ^T^1*, we then evaluated two subsequent pathways: (i) outer-sphere electron transfer from ^T^1* to CF_3_SO_2_Cl *via* the Marcus crossing point (MCP) MCP1 and (ii) relaxation back to the ground-state complex 1 through MECP3. MECP3 lies 12.7 kcal mol^−1^ above MECP2, suggesting that, once formed, ^T^1* is much less prone to non-radiative relaxation than ^S^1*.

MCP1 is found to lie 1.6 kcal mol^−1^ higher in energy than MECP2, indicating a competition between relaxation and electron transfer. Although relaxation from the excited state may also occur through radiative pathways, this small energy difference is reasonably consistent with the experimental quantum yield of 12% reported by Reiser *et al.*^40^

It should finally be noted that, although MECPs do not provide quantitative ISC rates, their relative positions nevertheless offer valuable insight into the accessibility of crossing events. In practice, ISC rates are determined not only by the location of MECPs but also by additional factors such as spin–orbit coupling,^77^ yet MECP energetics remain a useful qualitative tool for identifying feasible crossings in photochemical mechanisms.

### Mechanism for the formation of the ˙CF_3_ radical as a key intermediate

**Fig. 3:**
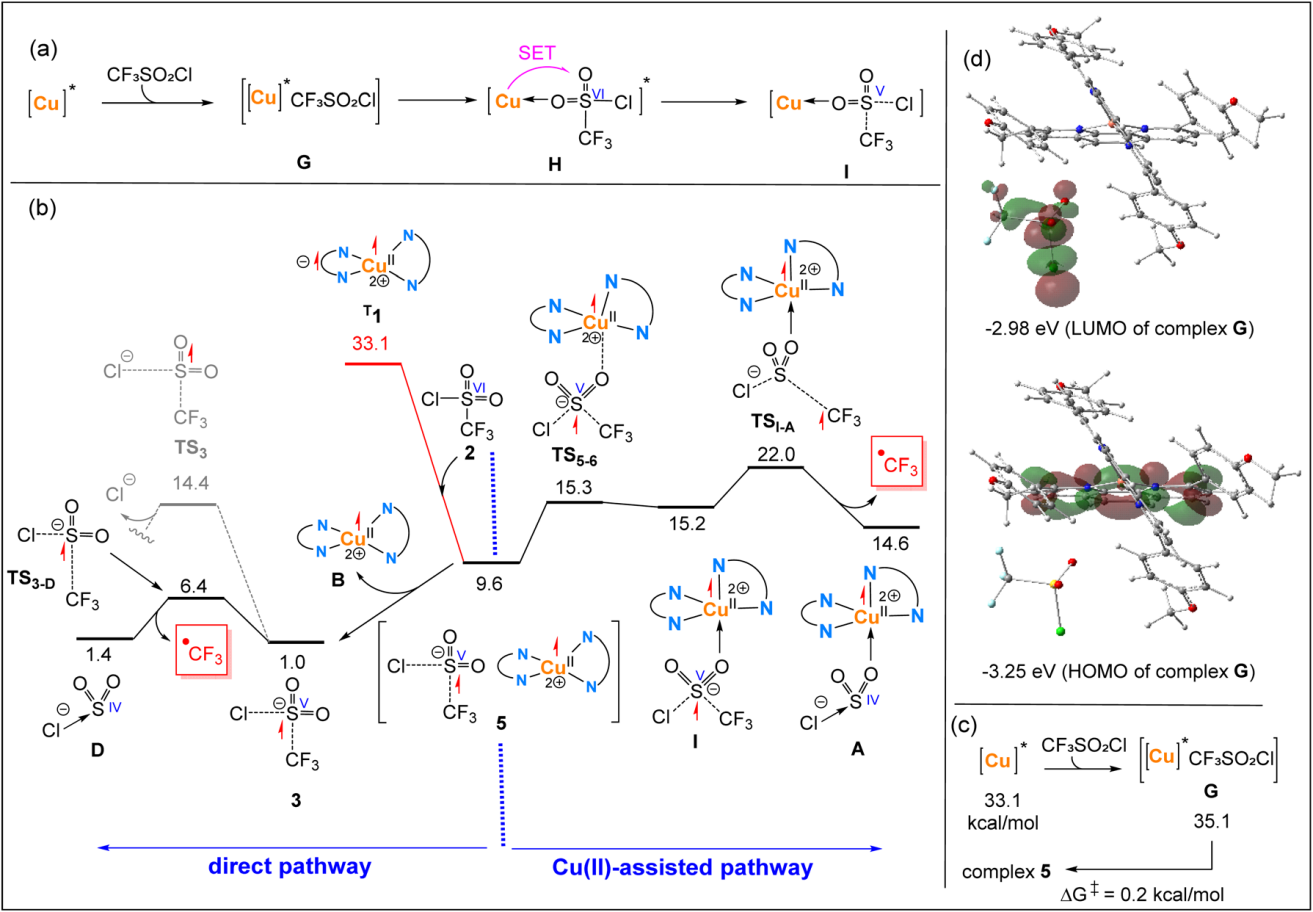
(a) Simplified schematic for the proposed inner-sphere single-electron transfer (SET) from the excited copper complex [Cu]^*^ to CF_3_SO_2_Cl. (b) Free energy profiles comparing Cu(ii)-assisted and direct pathways for *in situ* generation of the ˙CF_3_ radical. Relative free energies are given in kcal mol^−1^ and calculated at the SMD/B3LYP-D3/def2-TZVP//SMD/B3LYP-D3/def2-SVP level in acetonitrile. (c) Calculated mechanism for the conversion of outer-sphere complex G to 5, obtained using a larger basis set (BS3) for geometry optimization. (d) Frontier molecular orbitals (HOMO and LUMO) of complex G with corresponding energies, obtained from single-point calculations at the SMD/B3LYP-D3/def2-TZVP level of theory, highlighting its high reactivity toward SET.

Now, we investigate whether the SET process leading to the formation of the key ˙CF_3_ intermediate can proceed *via* an inner-sphere mechanism. For this to occur, the first step involves the formation of an outer-sphere complex G (Fig. 3a), where CF_3_SO_2_Cl, with the sulfur atom in a formal oxidation state of +6, is positioned near the coordination sphere of [Cu]*, after overcoming the energy barrier associated with bringing these two species into proximity. Then, CF_3_SO_2_Cl must coordinate to [Cu]^*^ to form intermediate H, in which orbital interaction with the metal centre is established, a prerequisite for SET *via* the inner-sphere mechanism.^78^ Finally, CF_3_SO_2_Cl accepts an electron from the excited [Cu]^*^ complex, leading to the formation of intermediate I, in which the sulfur atom adopts a formal oxidation state of +5.

Based on our calculations at the SMD/B3LYP-D3/def2-SVP level of theory, all attempts to optimize the outer-sphere complex G (Fig. 3b) consistently resulted in spontaneous electron transfer from [Cu]^*^ to CF_3_SO_2_Cl, yielding intermediate 5. This is evidenced by the increase in total spin density on CF_3_SO_2_Cl in intermediate 5, reaching 0.653, along with the elongation of the calculated S–Cl and S–CF_3_ bond distances from 2.112 and 1.910 Å in 2 to 2.727 and 1.912 Å in 5, respectively. The electron transfer renders the formation of the outer-sphere complex 5 highly exergonic, with a calculated free energy release of approximately 23.5 kcal mol^−1^ (Fig. 3b). This finding supports the conclusion that the SET process should proceed *via* an outer-sphere mechanism, because in the assumed outer-sphere complex G the two redox partners remain separated, without the orbital overlap required for an inner-sphere SET pathway.

To further validate the conclusion that electron transfer between [Cu]^*^ and CF_3_SO_2_Cl occurs essentially immediately once the outer-sphere complex G is formed, we employed a larger basis set (BS3) for its optimization (BS3: def2-TZVP for Cu, Cl and S, and def2-SVP for other atoms). Interestingly, this time we successfully located outer-sphere complex G; however, we found that it readily converts to the more stable complex 5 with an activation energy as low as 0.2 kcal mol^−1^ (Fig. 3c). This much lower barrier (0.2 kcal mol^−1^), compared with the ∼8 kcal mol^−1^ barrier obtained for MCP1 in Fig. 2, can be rationalized by Marcus theory: at longer donor–acceptor distances a substantial barrier exists, whereas at near-contact the reduced distance between the redox partners greatly increases the probability of electron transfer, making the process essentially barrierless. This behaviour is fully consistent with Marcus theory, in which the outer-sphere SET barrier decreases as the distance between the redox partners is reduced.^79,80^

An analysis of the frontier orbitals of complex G at the SMD/B3LYP/def2-TZVP level of theory provides insight into why this complex is highly reactive toward the SET process through the outer-sphere mechanism (Fig. 3d). The HOMO in this complex is primarily characterized by a π* orbital of the dap ligand that holds the unpaired electron, while the LUMO is predominantly composed of the σ* orbitals of the S–Cl bond (major contribution) and the S–CF_3_ bond (minor contribution) in CF_3_SO_2_Cl. The energy gap between the HOMO and LUMO in complex G is only 0.27 eV, which is sufficiently small to explain why the single electron transfer from the π* orbital of dap to the lowest lying σ* orbital of CF_3_SO_2_Cl occurs almost spontaneously.

From complex 5, we investigated two different pathways for the formation of ˙CF_3_: (i) a Cu(ii)-assisted dissociation pathway and (ii) a direct dissociation pathway (Fig. 3b).

In the Cu(ii)-assisted pathway, the [CF_3_SO_2_Cl]˙^−^ radical anion must first coordinate to the Cu(ii) centre, forming complex I. For this coordination to occur, the four-coordinate Cu(ii) complex, which adopts a distorted square planar geometry^76^ in complex 5, must undergo a rearrangement to a trigonal pyramidal geometry to create an open coordination site for [CF_3_SO_2_Cl]˙^−^. This structural rearrangement is energetically demanding, making complex I 5.6 kcal mol^−1^ less stable than complex 5. The release of the ˙CF_3_ radical from complex I proceeds through the transition structure TS_I–A_, with a relative free energy of 22.0 kcal mol^−1^, leading to the formation of complex A. This radical dissociation process induces a formal oxidation state change of the sulfur atom from +5 to +4.

In the direct dissociation pathway, the outer-sphere complex 5 undergoes fragmentation to yield the Cu(ii) complex B and the S(v) species 3 in a thermodynamically favourable process. From intermediate 3, we investigated two possible pathways: (a) dissociation of Cl^−^ through TS_3_ and (b) dissociation of ˙CF_3_ through TS_3-D_. The calculations clearly indicate that 3 is significantly more reactive toward ˙CF_3_ dissociation, as evidenced by the fact that TS_3-D_ lies 8.0 kcal mol^−1^ lower in energy than TS_3_.

From a comparison of the two pathways presented in Fig. 3b, it is evident that TS_3-D_ is approximately 15.6 kcal mol^−1^ lower in energy than TS_I–A_, indicating that ˙CF_3_ release preferentially occurs through the direct dissociation pathway rather than the Cu(ii)-assisted pathway. This conclusion is further supported by calculations at the SMD/wB97XD/def2-TZVP//SMD/B3LYP-D3/def2-SVP level of theory, where in this case TS_3-D_ lies about 19.9 kcal mol^−1^ lower in energy than TS_I–A_ (see Fig. S1).

The results presented in Fig. 3b offer an opportunity to revisit and refine the mechanistic interpretation proposed by Pham *et al.* in a recent publication.^68^ In their computational study, they proposed that the transformation I → TS_I–A_ → A + ˙CF_3_ corresponds to an inner-sphere SET process. However, our detailed analysis reveals that this transformation actually represents the Cu(ii)-assisted release of the ˙CF_3_ radical after the SET has already occurred from [Cu]^*^ to CF_3_SO_2_Cl *via* an outer-sphere mechanism.

### Stability of intermediate D ([SO_2_Cl]^−^) and formation of R˙

**Fig. 4:**
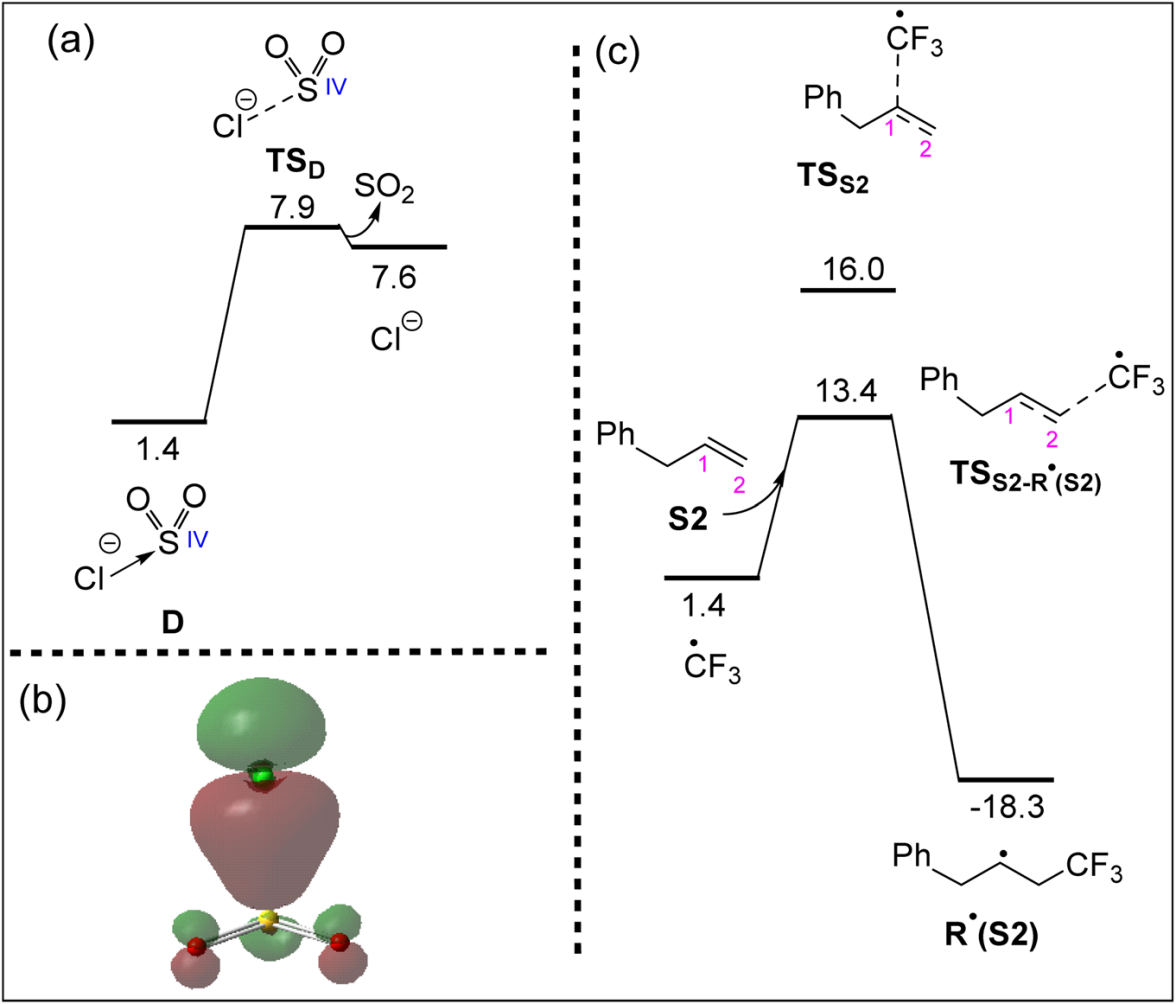
(a) Calculated free energy profile for the potential dissociation of intermediate D into SO_2_ and Cl^−^. (b) Natural localized molecular orbital (NLMO) representation of the stabilizing orbital interaction between the lone pair on Cl^−^ and the π* orbital of SO_2_ in intermediate D. (c) Free energy profile for the regioselective addition of the ˙CF_3_ radical to substrate S2, demonstrating a lower activation barrier for addition to the C2 position. Relative free energies are given in kcal mol^−1^ and calculated at the SMD/B3LYP-D3/def2-TZVP//SMD/B3LYP-D3/def2-SVP level in acetonitrile.

As depicted in Fig. 3b, the direct dissociation pathway leads to the formation of intermediate D and the ˙CF_3_ radical. This raises the question of whether intermediate D can further dissociate to yield SO_2_ and Cl^−^. However, our calculations indicate that this process is thermodynamically unfavourable, with an endergonic energy cost of approximately 6.5 kcal mol^−1^ (Fig. 4a). The stability of intermediate D is attributed to a strong orbital interaction between a lone pair on Cl^−^ and the SO_2_ π* orbital, as confirmed by NLMO analysis (Fig. 4b). This interaction with a second-order perturbation energy (*E*^2^) of 51.4 kcal mol^−1^, is sufficiently strong.

As previously proposed,^68^ once the ˙CF_3_ radical is formed, it is added to the alkene substrate during the catalytic cycle to form R˙. Here, we begin our discussion by focusing on substrate S2 (Fig. 1b), while the computational analysis of the reactivity of other substrates depicted in Fig. 1a, c, and d will be addressed later. The ˙CF_3_ radical can selectively add to either the C1 or C2 position of substrate S2. As shown in Fig. 4c, the addition to the C2 atom proceeds with a significantly lower activation barrier compared to C1, a result that aligns well with the experimental observations.^68^

### Formation of products P1(S2) and P2(S2) assisted by Cu(ii) complexes

**Fig. 5:**
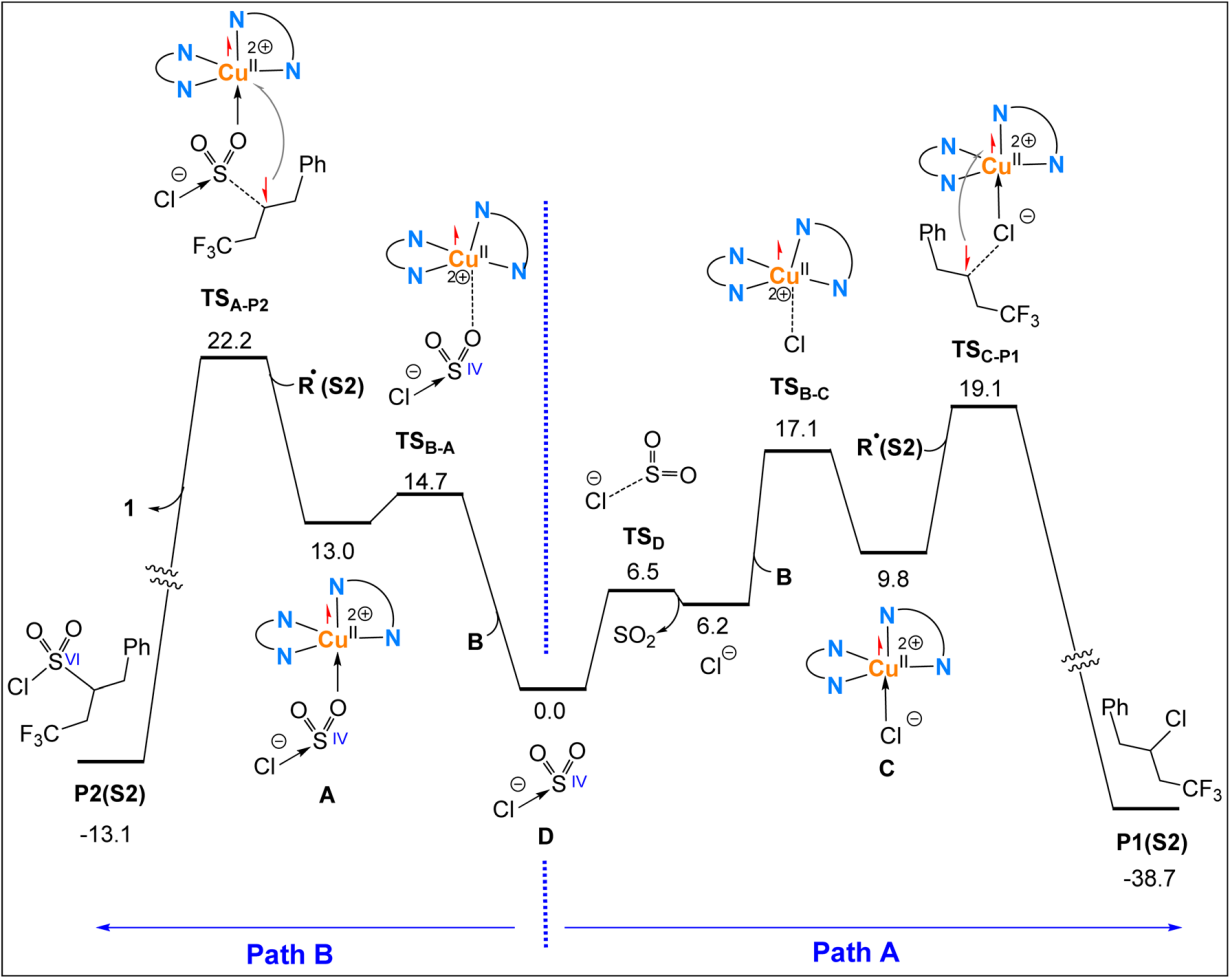
Computed free energy profiles for Cu(ii)-assisted formation of P1(S2) and P2(S2)*via* pathways A and B. Relative free energies are given in kcal mol^−1^ and calculated at the SMD/B3LYP-D3/def2-TZVP//SMD/B3LYP-D3/def2-SVP level in acetonitrile.

As discussed in the Introduction (Fig. 1f), Pham *et al.* proposed that once the R˙ radical is formed, it preferentially reacts with five-coordinate complexes [Cu^II^Cl] or [Cu^II^SO_2_Cl] to afford the final products P1 or P2. We re-evaluated the feasibility of this proposal by locating all relevant transition structures and intermediates involved in the processes originating from species D along pathways A and B (Fig. 5), using the R˙ radical derived from substrate S2, R˙(S2), as the key intermediate.

Pathway A (Fig. 5) begins with the fragmentation of species D into SO_2_ and Cl^−^, followed by coordination of the resultant Cl^−^ to the four-coordinate Cu(ii) complex B to form [Cu^II^Cl] (complex C). Finally, coupling between the R˙ radical and the Cl ligand in complex C on the symmetry-broken open-shell singlet surface facilitates single-electron transfer from the R˙ radical to the Cu(ii) centre *via* transition structure TS_C–P1_, ultimately affording the product P1(S2) and regenerating the Cu(i) catalyst 1.

Pathway B (Fig. 5) begins with the coordination of species D to the Cu(ii) complex B through one of its oxygen atoms, *via* the transition structure TS_B–A_, to form complex A. Subsequently, the R˙ radical attacks the sulfur atom in complex A, promoting the single electron on R˙ to transfer to the Cu(ii) centre *via* the transition structure TS_A–P2_ located on the symmetry-broken open-shell singlet surface, thereby forming the P2(S2) product.

A comparison of pathways A and B reveals that pathway A is energetically more favourable, as the highest energy point along this route (TS_C–P1_) lies 3.1 kcal mol^−1^ below the corresponding transition structure in pathway B (TS_A–P2_). This difference becomes even more pronounced at the SMD/wB97XD/def2-TZVP//SMD/B3LYP-D3/def2-SVP level of theory in acetonitrile, where TS_C–P1_ lies 8.7 kcal mol^−1^ lower in energy than TS_A–P2_ (Fig. S2). These findings suggest that if the final step of the reaction proceeds *via* a Cu(ii)-mediated mechanism, product P1(S2) would be expected as the predominant outcome. However, this prediction does not align with experimental observations, which show that when substrate S2 is employed, product P2 is formed preferentially (Fig. 1b). Together, these results indicate that alternative, more favourable mechanisms beyond those mediated by Cu(ii) complexes must be operative to account for the experimentally observed chemoselectivity. In the following sections, we demonstrate how our proposed outer-sphere mechanisms (i)–(iii), as introduced in the Introduction, rationalize the observed selectivity.

### Formation of products P1(S2) and P2(S2)*via* more favourable outer-sphere mechanisms

**Fig. 6:**
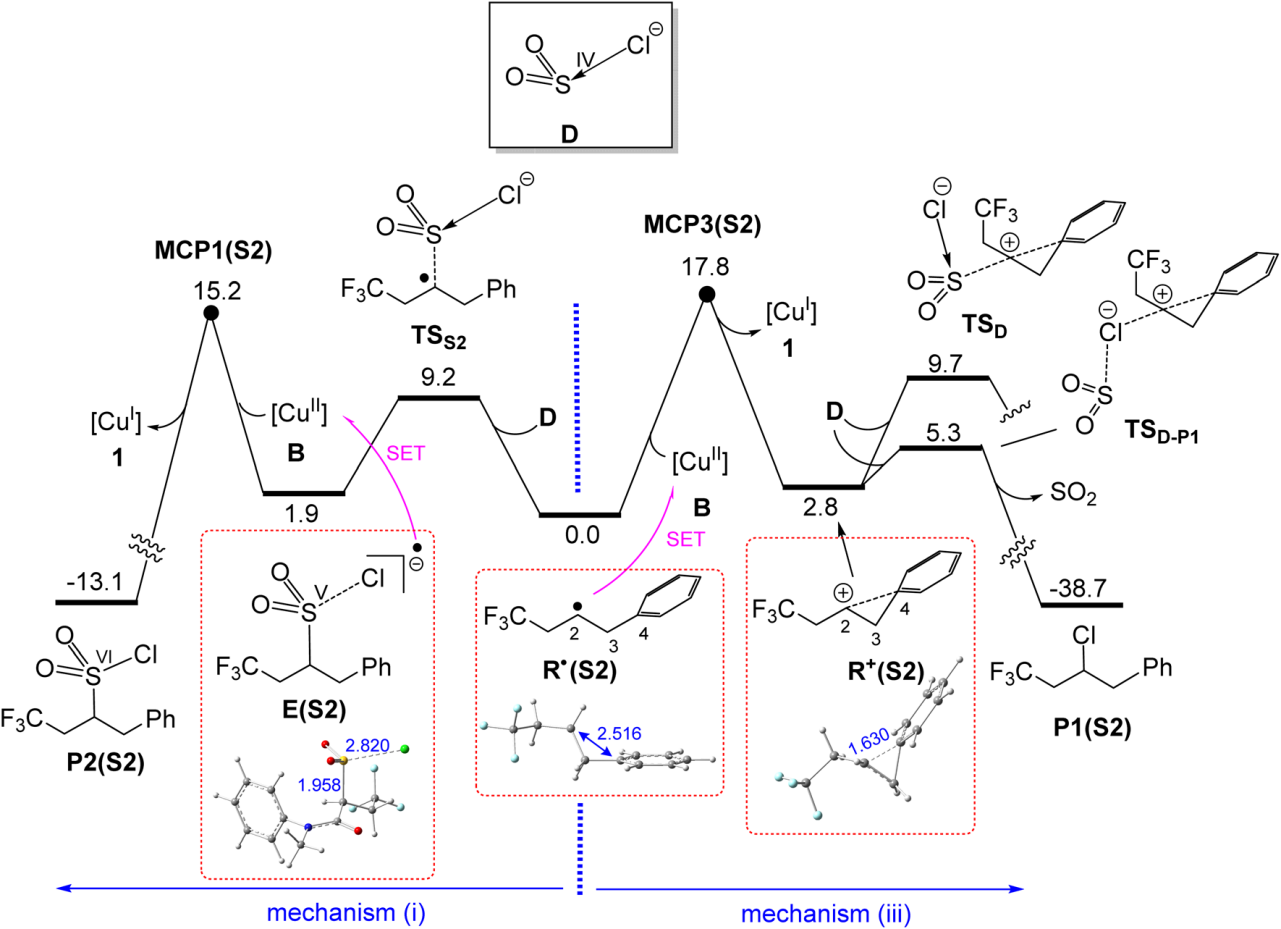
Calculated free energy profiles for formation of P1(S2)*via* mechanism (iii) and P2(S2)*via* mechanism (i). Relative free energies are given in kcal mol^−1^ and calculated at the SMD/B3LYP-D3/def2-TZVP//SMD/B3LYP-D3/def2-SVP level in acetonitrile. Distances are given in Å. Selected bond distances are shown in blue (Å).

In this subsection, we demonstrate that the formation of both products P1(S2) and P2(S2)*via* outer-sphere pathways proceeds with substantially lower activation barriers compared to the inner-sphere mechanisms mediated by Cu(ii) complexes A and C (Fig. 5). We have investigated two outer-sphere mechanisms, denoted as mechanisms (i) and (iii) in Fig. 6.

Mechanism (i), responsible for the formation of product P2(S2), was introduced earlier in the Introduction (Fig. 1h). In this pathway, the anion D (SO_2_Cl^−^) reacts with the radical R˙(S2)*via* transition structure TS_S2_, forming intermediate E(S2) (Fig. 6). From this intermediate, an electron is transferred to the Cu(ii) complex B through the Marcus crossing point MCP1(S2), ultimately resulting in the formation of product P2(S2) and the regeneration of the Cu(i) catalyst 1.

Mechanism (iii), which leads to the formation of product P1(S2), involves the transfer of an electron from the radical R˙(S2) to the Cu(ii) complex B*via* the Marcus crossing point MCP3(S2), resulting in the formation of the carbocation R^+^(S2) and the regeneration of the Cu(i) catalyst 1 (Fig. 6). This carbocation gains partial stabilization through interaction with the phenyl substituent, as evidenced by the shortening of the C2–C4 distance from 2.516 Å in R˙(S2) to 1.630 Å in R^+^(S2); the CASSCF results supported by spin-density analysis suggest that no electronic interaction exists between C2 and C4 in R˙(S2) (Fig. S3). Finally, R^+^(S2) is trapped by the Cl substituent of SO_2_Cl^−^*via* transition structure TS_D–P1_, followed by SO_2_ release, to yield product P1(S2). In contrast, trapping of R^+^(S2) through the S atom of SO_2_Cl^−^, involving transition structure TS_D_, requires a much higher activation free energy (Fig. 6). This difference reflects the greater nucleophilicity of the Cl substituent compared to the S atom in SO_2_Cl^−^, explaining why carbocation R^+^(S2) preferentially reacts with SO_2_Cl^−^ to form product P1(S2) rather than product P2(S2).

It follows from Fig. 6 that the highest energy point on mechanism (i), namely MCP1(S2), lies 2.6 kcal mol^−1^ lower in energy than the corresponding point on mechanism (iii), MCP3(S2). This energy difference indicates that the formation of P2(S2) is more favourable than that of P1(S2), thereby explaining the experimentally observed chemoselectivity for substrate S2, for which P2(S2) is the dominant product (Fig. 1b).

It is worth noting that for substrate S2, mechanism (ii) outlined in Fig. 1i is unlikely to be operative, as we were unable to locate intermediate F featuring the proposed 2c–3e C–Cl bond. This appears to be due to the full localization of the unpaired electron on the C2 atom in R˙(S2), which prevents the orbital overlap required to stabilize a C–Cl 2c–3e bond and thus precludes the formation of intermediate F.

### Formation of products P1(S1) and P2(S1)*via* outer-sphere mechanisms

**Fig. 7:**
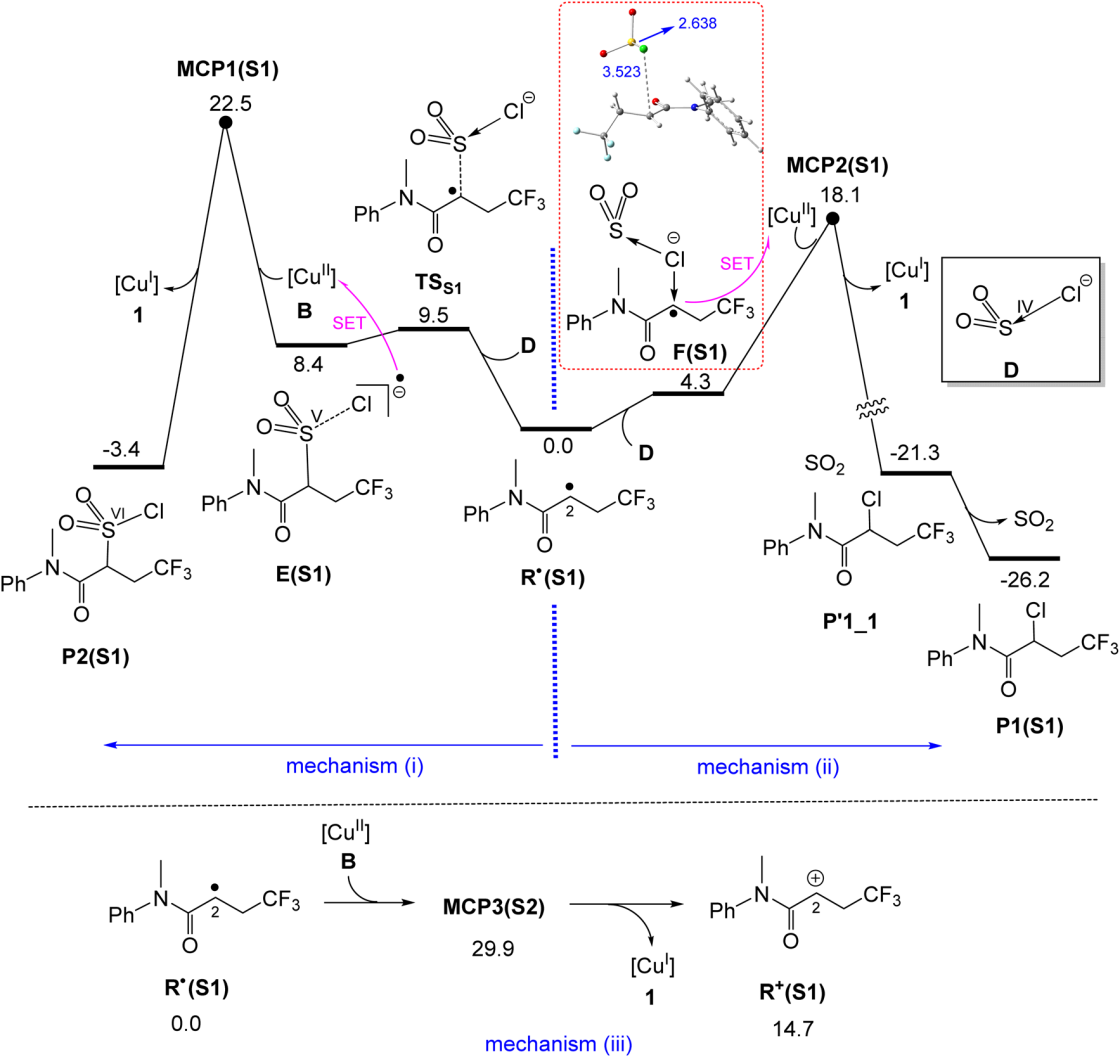
Calculated free energy profiles for formation of P1(S1)*via* mechanism (ii) and P2(S1)*via* mechanism (i) and calculated mechanism for formation of carbocation R^+^(S1)*via* mechanism (iii). Relative free energies are given in kcal mol^−1^ and calculated at the SMD/B3LYP-D3/def2-TZVP//SMD/B3LYP-D3/def2-SVP level in acetonitrile. Selected bond distances are shown in blue (Å).

As discussed in the Introduction, Dolbier *et al.* reported that irradiation of the electron-deficient alkene S1 with CF_3_SO_2_Cl in the presence of the Cu(i) catalyst 1 leads exclusively to trifluoromethylchlorination, with SO_2_ extrusion, yielding P1(S1) (Fig. 1a).^39^ In this subsection, we aim to elucidate the mechanistic origin of this observed selectivity. To this end, we have examined all three proposed outer-sphere mechanisms (i)–(iii) (Fig. 7).

For the electron-deficient substrate S1, we found that mechanism (ii) is indeed feasible. This is because the single electron on the resulting R˙ radical is not exclusively localized on the C2 atom but is delocalized between the C2 and O atoms, as evidenced by the spin density values of 0.852 for C2 and 0.120 for O. This delocalization significantly enhances the accessibility of the C2 atom for interaction with the Cl atom of the SO_2_Cl^−^ anion (species D), leading to the formation of intermediate F(S1), which features a characteristic C–Cl 2c–3e bond. This intermediate then transfers an electron to the four-coordinate Cu(ii) complex B*via* the Marcus crossing point MCP2(S1) with relative energy of 18.1 kcal mol^−1^, ultimately yielding product P1(S1).

Although the delocalization of the single electron in radical R˙(S1) facilitates its interaction with the Cl atom of the SO_2_Cl^−^ anion, it concurrently reduces its ability to oxidize the S(iv) centre in SO_2_Cl^−^ to S(v). As a result, the formation of intermediate E(S1) is energetically disfavoured, lying 8.4 kcal mol^−1^ above R˙(S1). This diminished oxidative capability plays a key role in positioning MCP1(S1) 4.4 kcal mol^−1^ higher in energy than MCP2(S1), thereby explaining why no product P2 is formed when electron-deficient alkenes such as S1 are used in the photocatalytic process.

Mechanism (iii) is highly unfavourable for this substrate due to its electron-deficient nature, which results in MCP3(S1) having a relative energy of 29.9 kcal mol^−1^, effectively ruling out its feasibility (Fig. 7). Consequently, based on the results presented in Fig. 7, it can be concluded that for the electron-deficient substrate S1, mechanism (ii) is the operative pathway, leading to the exclusive formation of product P1. This finding is in excellent agreement with the experimental observations.^68^

The CASSCF results supported by spin-density analysis for E(S1) show that the single unpaired electron mainly occupies the σ* orbital of the S–Cl bond, whereas the σ* orbital of the S–C bond is almost completely empty. This finding demonstrates that the formal oxidation state of sulfur in this species is best described as +V. The same analysis for F(S1) reveals that the C–Cl 2c–3e bond is highly polar, with the unpaired electron density residing primarily on the C atom (for details, see Fig. S3).

### Formation of products P1(S3) and P2(S3)*via* outer-sphere mechanisms

**Fig. 8:**
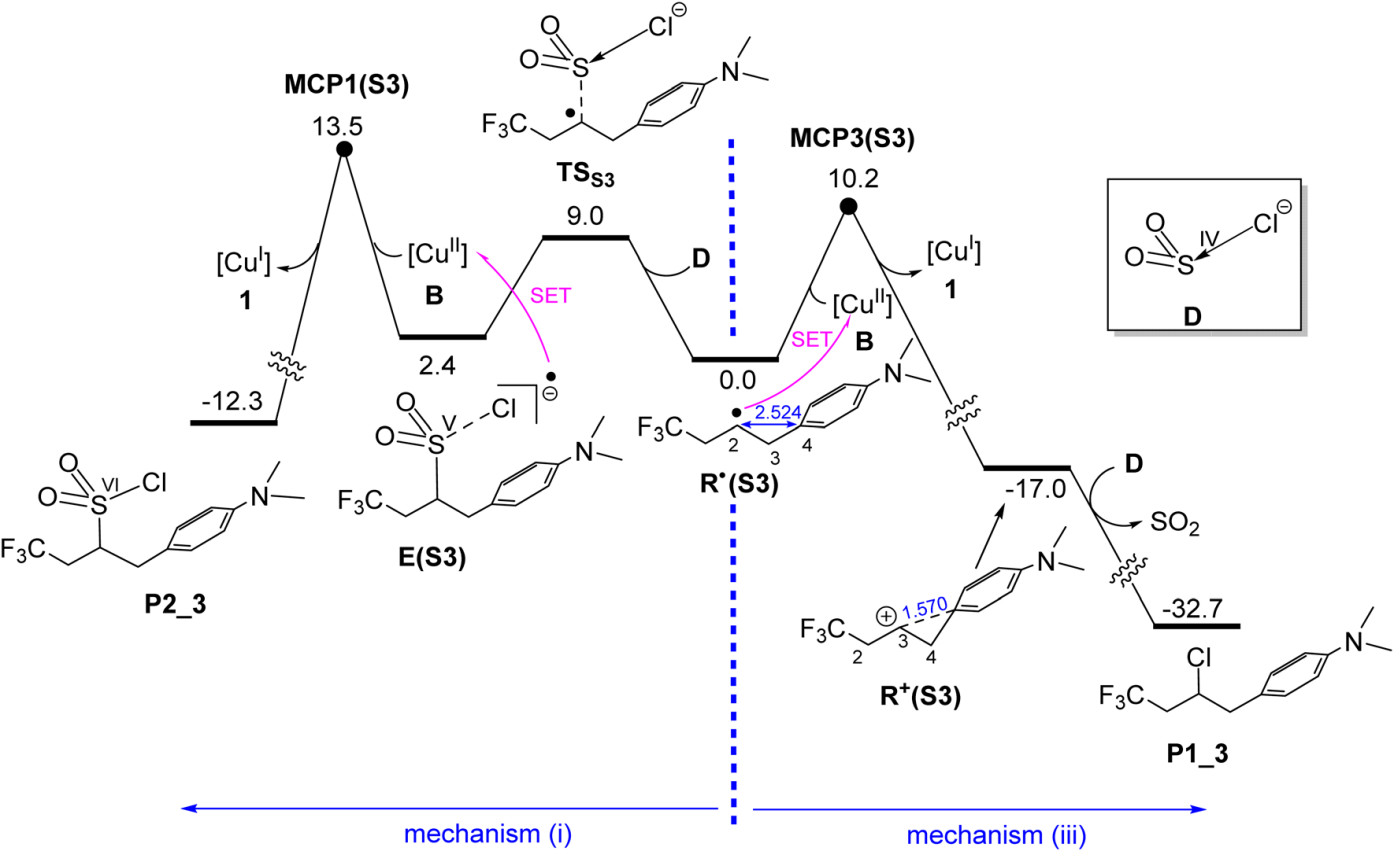
Calculated free energy profiles for formation of P1(S3)*via* mechanism (iii) and P2(S3)*via* mechanism (i). Relative free energies are given in kcal mol^−1^ and calculated at the SMD/B3LYP-D3/def2-TZVP//SMD/B3LYP-D3/def2-SVP level in acetonitrile. Selected bond distances are shown in blue (Å).

As discussed in the Introduction, while substrate S2 predominantly yields product P2 (Fig. 1b), para substitution of its phenyl ring with an NMe_2_ group (giving substrate S3, Fig. 1c) completely reverses the chemoselectivity, resulting in exclusive formation of product P1 under identical catalytic conditions. Our calculations show that this substitution significantly shifts the mechanistic preference toward mechanism (iii), as evidenced by MCP3(S3) lying 3.3 kcal mol^−1^ lower in energy than MCP1(S3) (Fig. 8). This shift is attributed to the strong electron-donating effect of the NMe_2_ group, which stabilizes the *in situ* generated carbocation through interaction with the phenyl ring, as illustrated in Fig. 8. Consequently, the single-electron transfer (SET) from R˙(S3) to the Cu(ii) centre proceeds with a much lower activation barrier (10.2 kcal mol^−1^, Fig. 8) compared to the corresponding SET from R˙(S2) (17.8 kcal mol^−1^, Fig. 6), explaining the observed chemoselectivity switch.

### Formation of products P1(S4) and P2(S4)*via* outer-sphere mechanisms

**Fig. 9:**
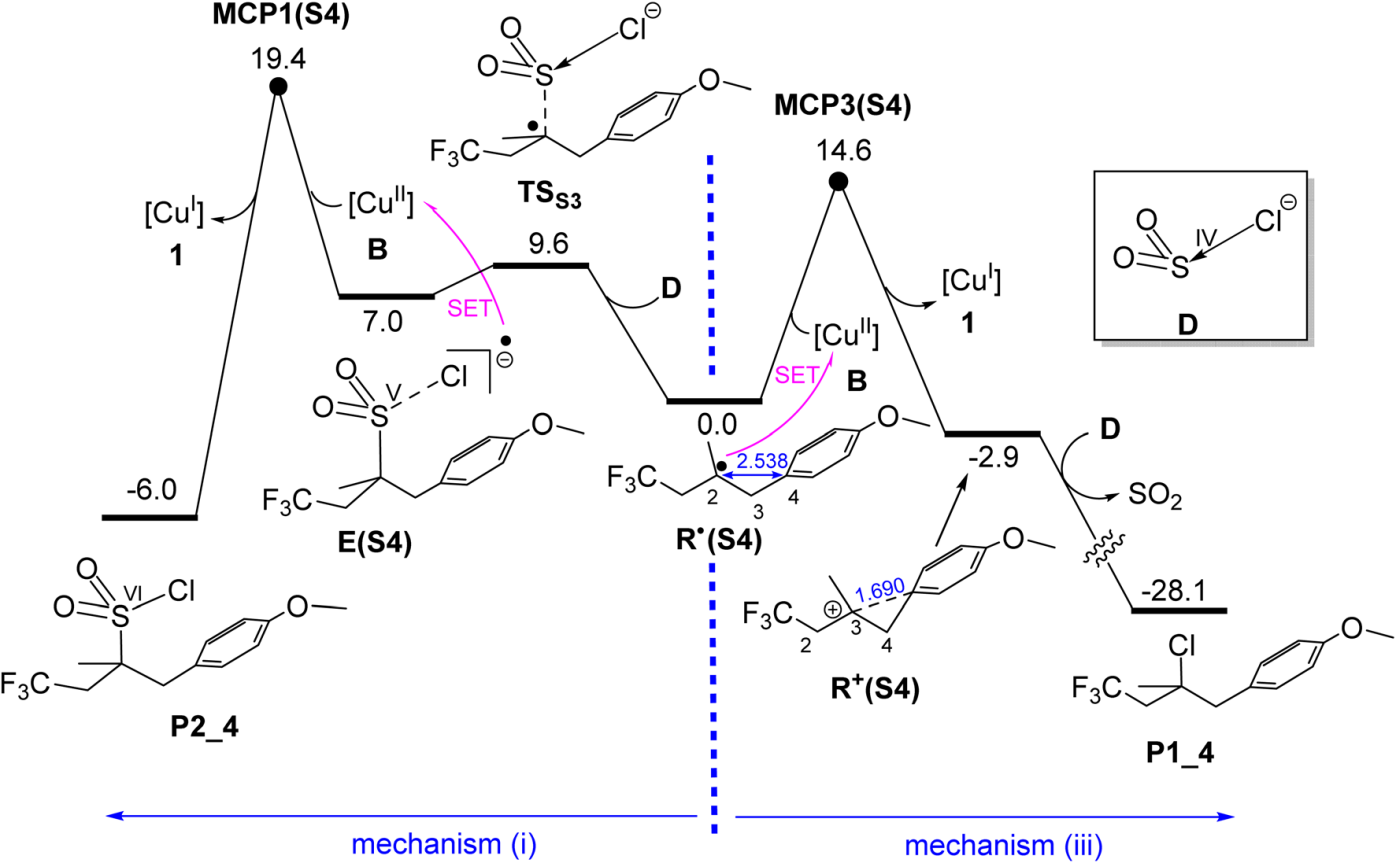
Calculated free energy profiles for formation of P1(S4)*via* mechanism (iii) and P2(S4)*via* mechanism (i). Relative free energies are given in kcal mol^−1^ and calculated at the SMD/B3LYP-D3/def2-TZVP//SMD/B3LYP-D3/def2-SVP level in acetonitrile. Selected bond distances are shown in blue (Å).

As discussed in the Introduction (Fig. 1), employing alkene substrate S4 instead of S2 in the catalytic process results in the exclusive formation of product P1 rather than P2. As shown in Fig. 9, the radical generated from S4, R˙(S4), is a tertiary radical, in contrast to the secondary radical R˙(S2) derived from substrate S2. Our calculations indicate that this tertiary radical is less prone to oxidize the sulfur centre from S(iv) to S(v), as evidenced by the formation of intermediate E(S4) from R˙(S4) and SO_2_Cl^−^ being significantly endergonic (Δ*G* = +7.0 kcal mol^−1^). This reduced oxidative propensity can be attributed to the greater intrinsic stability of tertiary radicals relative to secondary ones, rendering mechanism (i) significantly more energy demanding. As a result, the single-electron transfer (SET) process in mechanism (i) *via*MCP1(S4) proceeds with a substantially higher activation energy compared to the competing mechanism (iii) *via*MCP3(S4). The calculated energy difference between these two pathways, 4.8 kcal mol^−1^ in favour of MCP3(S4), strongly supports the operation of mechanism (iii), thereby explaining the exclusive formation of product P1(S4) when substrate S4 is employed.

### Evaluating the oxidative power of four- *vs.* five-coordinate Cu(ii) complexes

**Fig. 10:**
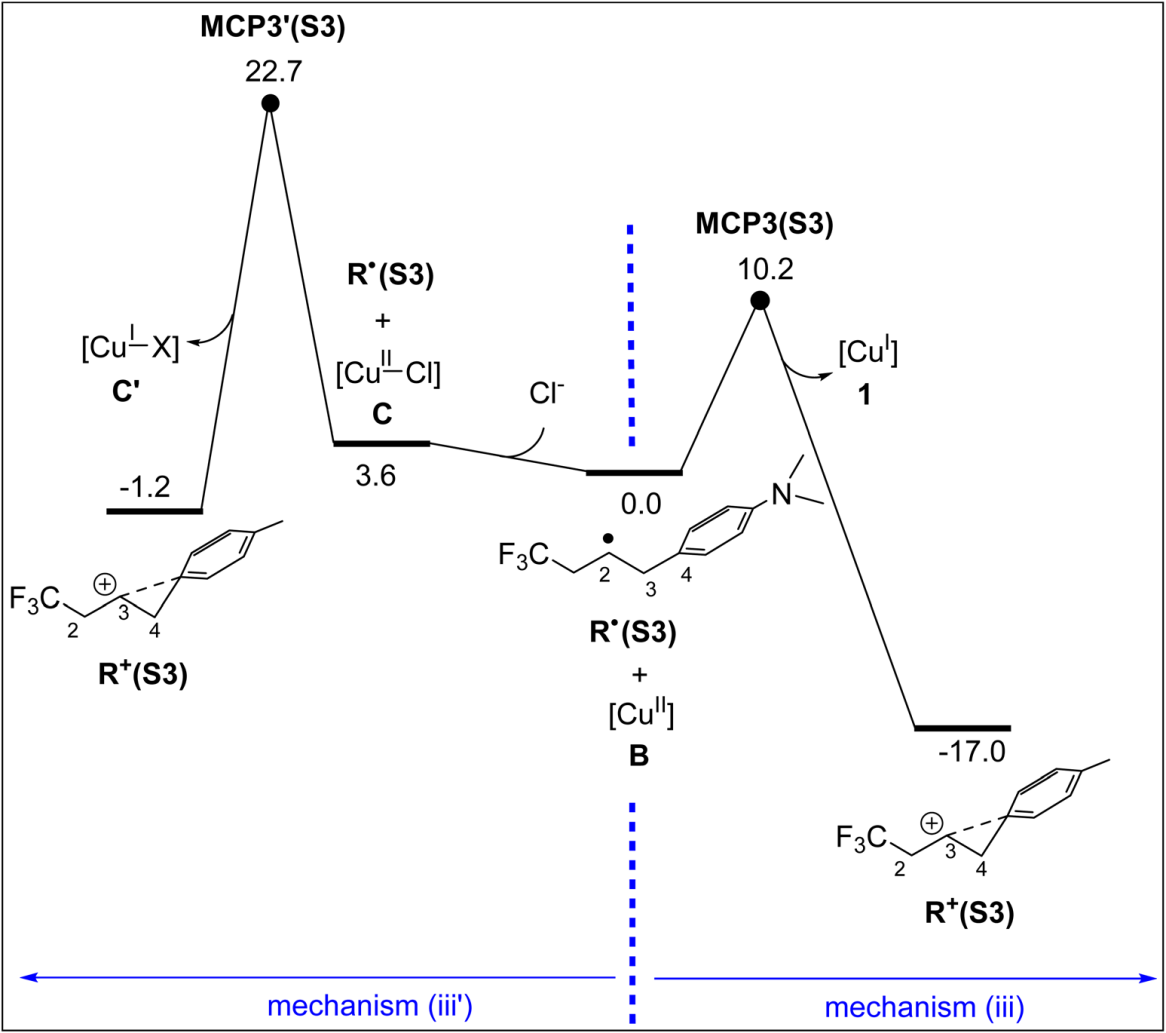
Free energy profiles comparing the oxidation of R˙(S3) to R^+^(S3) by four-coordinate [Cu^II^] and five-coordinate [Cu^II^–Cl] complexes. Relative free energies are given in kcal mol^−1^ and calculated at the SMD/B3LYP-D3/def2-TZVP//SMD/B3LYP-D3/def2-SVP level in acetonitrile.

As discussed in the Introduction, a recent computational study considered a five-coordinate Cu(ii) complex, [Cu^II^–X], as an oxidant for converting a carbon-centred radical R˙ into a carbocation R^+^ (Fig. 1g).^68^ This subsection aims to demonstrate that the four-coordinate [Cu^II^] complex 1 is a significantly more effective oxidant than [Cu^II^–X]. To evaluate this, we assume the coordination of the counterion Cl^−^ in the [Cu(dap)_2_]Cl catalyst to complex B, forming the five-coordinate complex C (Fig. 10). Our calculations show that complex C lies 3.6 kcal mol^−1^ higher in energy than the four-coordinate complex 1. As previously discussed, the low tendency of complex 1 to bind a fifth ligand stems from its distorted square-planar geometry, which must reorganize into a trigonal-pyramidal structure to accommodate an additional ligand, an energetically unfavourable process that renders complex C less stable than complex B.

Fig. 10 compares the free energy profiles for the oxidation of R˙(S3) to R^+^(S3) by [Cu^II^] and [Cu^II^–Cl]. As shown, the reaction R˙(S3) + [Cu^II^] → R^+^(S3) + [Cu^I^] is significantly more exergonic (Δ*G* = −17.0 kcal mol^−1^) than the corresponding process with [Cu^II^–Cl], which proceeds with a much smaller driving force (Δ*G* = −4.8 kcal mol^−1^). This thermodynamic difference is reflected in the position of the Marcus crossing points: MCP3(S3) lies 12.5 kcal mol^−1^ lower in energy than MCP3′(S3) (Fig. 10). These results clearly demonstrate that [Cu^II^] is a far superior oxidant compared to [Cu^II^–Cl]. The underlying reason is electronic: [Cu^II^] is a 17-electron species that becomes an 18-electron complex upon reduction, an electronically favourable transformation. In contrast, [Cu^II^–Cl] is a 19-electron species; accepting another electron would yield a 20-electron complex, significantly violating the 18-electron rule and making it a much less effective oxidant.

### Summary of the DFT-calculated mechanistic landscape

**Fig. 11:**
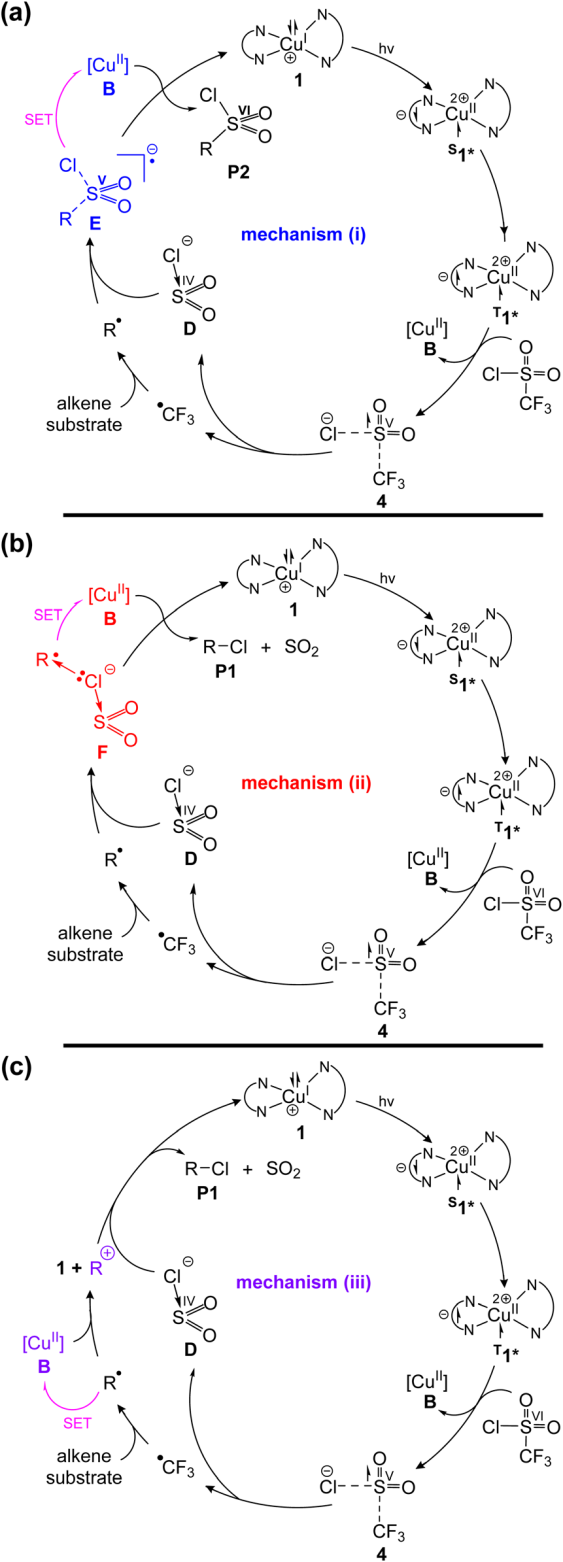
DFT-calculated mechanistic landscape for copper-photocatalyzed ATRA reactions of CF_3_SO_2_Cl with alkenes. The mechanism begins with outer-sphere single-electron transfer (SET) from the photoexcited copper complex [Cu]^*^ to CF_3_SO_2_Cl, generating SO_2_Cl^−^ and a ˙CF_3_ radical. The ˙CF_3_ radical then adds to the alkene to form the carbon-centred radical R˙, which furnishes the final product through one of three outer-sphere pathways: (a) S(vi)/S(iv) redox cycling (mechanism (i)), (b) 2c–3e Cl-coordination-induced SET (mechanism (ii)), or (c) direct radical oxidation to a carbocation (mechanism (iii)).

Fig. 11 provides a schematic summary of the DFT-calculated mechanistic landscape for copper-photocatalyzed ATRA reactions of CF_3_SO_2_Cl with alkenes. Upon photoexcitation of the [Cu(dap)_2_]^+^ catalyst 1, the resulting singlet excited state ^S^1* undergoes intersystem crossing to the more stable triplet state ^T^1*, which acts as a potent reducing agent. This triplet state initiates a single-electron transfer (SET) to CF_3_SO_2_Cl *via* an outer-sphere mechanism, leading to the formation of species 4. As a result of this SET process, the formal oxidation state of the sulfur atom decreases from +6 in CF_3_SO_2_Cl to +5 in species 4.

Species 4 then releases the ˙CF_3_ radical, further reducing the formal oxidation state of sulfur from +5 to +4 in the resulting SO_2_Cl^−^ anion. The generated ˙CF_3_ radical subsequently adds to the alkene substrate, forming the radical intermediate R˙, which serves as a key branching point for several plausible mechanistic pathways. We have explored three such pathways, mechanisms (i) to (iii), each capable of explaining the experimentally observed chemoselectivities arising from variations in alkene substrate structure.

Mechanism (i) (S^VI^/S^IV^ redox cycling – Fig. 11a): The R˙ radical is added to the sulfur atom of SO_2_Cl^−^ to form intermediate E, oxidizing the sulfur centre from S(iv) to S(v). A subsequent outer-sphere electron transfer from E to the Cu(ii) complex B regenerates the Cu(i) catalyst and affords product P2, in which the formal oxidation state of sulfur is restored to +6. As a result, when this mechanism is operative, the sulfur centre undergoes a stepwise redox sequence: S(vi) → S(v) → S(iv) → S(v) → S(vi), thus justifying the designation “S(vi)/S(iv) redox cycling”.

This mechanism is expected to be operative when intermediate E does not lie significantly higher in energy than the R˙ radical, or when the alternative R^+^ carbocation is not intrinsically very stable. Consequently, tertiary or electron-deficient R˙ radicals are less likely to oxidize S(iv) to S(v), resulting in a significantly less stable intermediate E and making this pathway less likely to operate.

Mechanism (ii) (2c–3e Cl-coordination-induced SET - Fig. 11b): The R˙ radical forms a weak C–Cl 2c–3e bond with the Cl atom of SO_2_Cl^−^, generating intermediate F. An electron is then transferred from this intermediate to the Cu(ii) complex B*via* an outer-sphere SET step, affording product P1 along with SO_2_ extrusion.

This mechanism is found to be operative for electron-deficient alkene substrates, where the radical centre in R˙ is adjacent to a π-acceptor carbonyl group. This electronic arrangement facilitates delocalization of the unpaired electron in R˙, partially unblocking the radical centre for formation of the weak 2c–3e bond and thereby stabilizing intermediate F as a genuine local minimum on the potential energy surface, ultimately enabling mechanism (ii) to proceed.

Mechanism (iii) (Fig. 11c): Alternatively, the R˙ radical directly transfers an electron to the Cu(ii) complex B, forming the carbocation R^+^. This highly reactive species is then trapped by SO_2_Cl^−^, yielding product P1 with concurrent SO_2_ extrusion. We found that this mechanism is operative when the R^+^ carbocation is intrinsically very stable.

### Assessment of the coordination-based mechanism involving dap ligand dissociation prior to SO_2_Cl^−^ coordination

Another possible pathway suggested by Reiser is that once [Cu(dap)_2_]^2+^ is formed, it undergoes ligand exchange, releasing one dap ligand to generate [Cu(dap)(Cl)(SO_2_Cl)].^71,72^ This Cu(ii) species can then react with the R˙ radical to yield the final product. Pham *et al.* examined this alternative pathway at the wB97XD level of theory and reported it to be highly unfavourable.^68^ For the sake of completeness, we also investigated this alternative pathway in more detail at the B3LYP-D3 level and likewise confirm that it is energetically unlikely (*vide infra*).

**Fig. 12:**
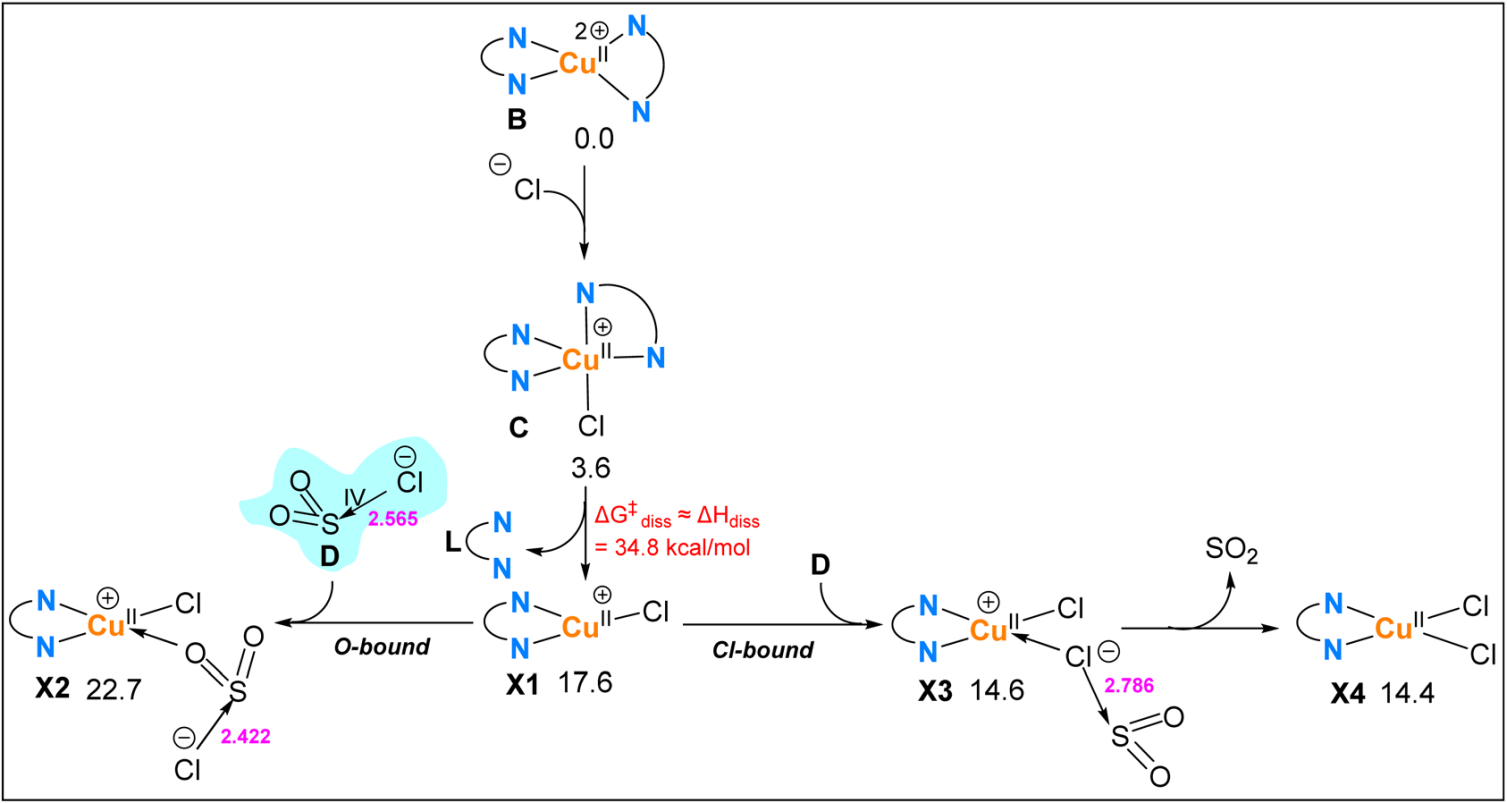
Mechanism for the coordination-based pathway leading from [Cu(dap)_2_]^2+^ to [Cu(dap)(Cl)(SO_2_Cl)] and its decomposition products calculated at the SMD/B3LYP-D3/def2-TZVP//SMD/B3LYP-D3/def2-SVP level in acetonitrile. The free-energy barrier for dap dissociation from complex C was estimated following the protocol of Hall and Hartwig,^81^ in which Δ*H*_diss_ for the dissociation process is taken to approximate 
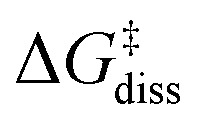
. Selected bond distances are shown in pink (Å), and relative free energies (kcal mol^−1^) are given with respect to intermediate B.

For this pathway to occur, we assumed that Cl^−^ first coordinates to [Cu(dap)_2_]^2+^, forming intermediate C (Fig. 12). One dap ligand can then dissociate, yielding structure X1. This dissociation process is endergonic by about 17.6 kcal mol^−1^. The resulting Cu(ii) species X1 can subsequently coordinate with SO_2_Cl^−^, either through its Cl atom or through one of the oxygen atoms. Our calculations show that coordination *via* the Cl atom is much more favourable, giving complex X3, which is 8.1 kcal mol^−1^ lower in energy than the O-coordinated species X2. However, X3 is highly reactive and readily releases SO_2_ gas, leading to the formation of X4.

From these results, two important conclusions can be drawn. (1) The relative free energy of intermediate X2 (22.7 kcal mol^−1^, Fig. 12), which would be the species attacked by the R˙ radical to form RSO_2_Cl, lies much higher than that of MCP1(S2) (15.2 kcal mol^−1^, Fig. 6). This comparison shows that this coordination-based pathway is considerably less favourable than the S(vi)/S(iv) redox cycling mechanism identified in this study. (2) The SO_2_Cl^−^ anion shows a clear preference for coordination to Cu(ii) through its Cl atom rather than through an oxygen atom. However, when bound through the Cl atom, SO_2_ is readily released, forming gaseous SO_2_ and complex X4. Therefore, if the reaction were to proceed *via* a coordination-based pathway, it would necessarily lead to R–Cl, because in this case the system is less likely to attain the Cu–OSOCl configuration that reacts with the R˙ radical to form RSO_2_Cl.

### Comment on the Ru *vs.* Cu mechanistic pathways

Reiser *et al.* found that for substrate S2 when [Cu(dap)_2_]^+^ is replaced with [Ru(bpy)_3_]^2+^, the reaction predominantly affords R–Cl but still gives ∼4% of RSO_2_Cl.^40^ This observation indicates that formation of RSO_2_Cl, P2(S2), must proceed through a non-coordinative pathway, since [Ru(bpy)_3_]^3+^ generated in solution is a coordinatively saturated octahedral complex incapable of binding SO_2_Cl^−^. The mechanisms proposed in this study, namely pathways (i) and (iii), provide an explanation for this result. Because Ru(iii) is a much stronger oxidant than Cu(ii), replacing Cu(ii) with Ru(iii) markedly lowers the energies of both MCP3(S2) and MCP1(S2), whereas the energy of TS_S2_ remains essentially unchanged (to follow the discussion, see Fig. 6, which shows the corresponding Cu(ii) results for substrate S2). Under these conditions MCP3(S2) is expected to lie slightly below TS_S2_, so that while R–Cl is the dominant product, a small amount of RSO_2_Cl is also formed. The greater oxidizing power of [Ru(bpy)_3_]^3+^ compared to [Cu(dap)_2_]^2+^ is also supported by our calculations: oxidation of radical R˙(S2) to carbocation R^+^(S2) by Ru(iii) is highly exergonic (≈−16 kcal mol^−1^), whereas with Cu(ii) it is only slightly endergonic (≈3 kcal mol^−1^, Fig. 6). It follows from the above discussion that the reduction potential of the oxidant (*e.g.*, Cu(ii) *versus* Ru(iii)) should play an important role in determining the product distribution.

### A final comment concerns stability and reactivity of SO_2_Cl^−^ in the presence of radicals

Although our calculations show that the decomposition of SO_2_Cl^−^ into SO_2_ and Cl^−^ is endergonic (Fig. 4), this anion has not been experimentally characterized as a persistent species. The absence of experimental observation may be attributed to the gaseous nature of SO_2_, which upon release leaves the system and thus hinders detection of SO_2_Cl^−^. Importantly, under the reaction conditions the presence of reactive radicals such as R˙ alters this scenario: SO_2_Cl^−^ can be rapidly trapped by R˙ to form the [RSO_2_Cl]˙^−^ radical anion, which is subsequently oxidized by Cu(ii). This radical-trapping pathway therefore prevents the otherwise expected decomposition of SO_2_Cl^−^ and channels it into the productive route leading to product P2.

## Conclusion

This study provides a comprehensive DFT-based analysis of the mechanism underlying copper-photocatalyzed ATRA reactions between CF_3_SO_2_Cl and alkenes. While a recent computational study proposed inner-sphere SET as the dominant pathway, our findings reveal that productive reactivity proceeds exclusively *via* outer-sphere mechanisms, which feature significantly lower activation barriers. The three mechanisms elucidated, namely S(vi)/S(iv) redox cycling, 2c–3e Cl coordination-induced SET, and the cationic pathway, collectively offer a framework that helps rationalize chemoselectivity in these copper-photocatalyzed transformations.

## Computational details

Gaussian 16^82^ was used to fully optimize all the structures reported in this paper at the B3LYP level of theory.^83–85^ We chose the B3LYP functional, as it has been demonstrated to provide good performance in computational studies of copper-catalyzed reactions.^86–92^ For all the calculations, solvent effects were considered using the SMD solvation model^93^ with acetonitrile as the solvent. Grimme's empirical dispersion correction (D3) was included in all optimization calculations.^94^ The def2-SVP basis set (BS1) was used for all atoms in the geometry optimizations. Frequency calculations were carried out at the same level of theory as those for structural optimization. Transition structures were located using the Berny algorithm. Intrinsic reaction coordinate (IRC) calculations were used to confirm the connectivity between transition structures and minima.^95^ The open-shell singlet transition structures for TS_A–P2_ and TS_C–P1_ (Fig. 5) were calculated using the broken-symmetry approach.^96–98^ To improve the precision of the energies obtained from the SMD/B3LYP-D3/def2-SVP calculations, we performed single-point energy calculations for all structures using the B3LYP-D3 density functional with a larger basis set in acetonitrile, modeled by SMD. This expanded basis set incorporates def2-TZVP (BS2) for all atoms.

The overall solvation free energy values reported in this work were obtained by adding the free energy corrections from the frequency analysis to the single-point solvation free energies calculated using the SMD solvation model. Moreover, the overall free energy values were corrected by adding Δ*G*^1atm→1M^ = 1.89 kcal mol^−1^ to account for the free-energy change associated with compressing 1 mol of an ideal gas from 1 atm to the 1 M solution phase standard state.

The activation free energies for outer-sphere single-electron transfer (SET) processes were calculated using the asymmetric Marcus theory, as recommended in the literature for obtaining more accurate barriers when the curvatures of the reactant and product potential energy surfaces differ.^99,100^ The solvent reorganisation contribution was obtained using the NonEq = write and NonEq = read keywords in Gaussian 16, which store and retrieve the nonequilibrium solvation effects arising from the reorganisation of the solvent cage in response to changes in the solute's electronic structure, as applied in previous studies.^101–103^

The respective minimum energy crossing points (MECPs), as reported in Fig. 2, were located using the code developed by Harvey and co-workers.^104^

To more accurately describe the electronic structure of the open-shell intermediates, multiconfigurational CASSCF calculations^105^ were performed for R˙(S3), F(S1), and E(S1). For F(S1) and E(S1), a (5,4) active space was employed, comprising five electrons in four orbitals corresponding to the σ and σ* orbitals of the C–S and S–Cl bonds. This selection effectively accounts for the bonding and antibonding interactions governing electron delocalization along these bonds. For R˙(S3), a (7, 7) active space was defined to include the six π electrons of the aromatic ring and the singly occupied p orbital on the β-carbon radical center, thereby encompassing the π/π* orbitals of the aromatic system and the benzylic radical orbital. This multireference treatment affords a more rigorous description of the electronic structure of these intermediates, complementing the DFT results and elucidating the extent of spin and charge delocalization. Because spin density is not rigorously defined for multiconfigurational wavefunctions, the distribution of unpaired electrons was examined through the odd-electron density (OED), computed using the Multiwfn 3.8 program.^106^ The OED provides a physically meaningful representation of the unpaired electron distribution, analogous to the spin density obtained from single-determinant methods.

## Author contributions

F. S. carried out most of the DFT, TD-DFT, and CASSCF calculations, performed data analysis, and contributed to drafting the paper. M. J. assisted with computational work and data analysis and contributed to drafting the paper. S. H. provided scientific guidance and co supervised the overall project. R. S. contributed to project discussion and finalisation. A. A. contributed to the computational work, led the data analysis, and supervised all aspects of the project and paper preparation.

## Conflicts of interest

There are no conflicts to declare.

## Supplementary Material

SC-017-D5SC06553D-s001

## Data Availability

All computational data supporting the findings of this study, including free energy profiles, Cartesian coordinates, calculated thermodynamic parameters, and CASSCF analyses of key intermediates, are provided in the supplementary information (SI). Supplementary information is available. See DOI: https://doi.org/10.1039/d5sc06553d.
