# Supplementary material for: Uncovering outer-sphere mechanisms governing chemoselectivity in copper-photocatalyzed ATRA reactions of CF3SO2Cl with alkenes
Source: Chem Sci. 2025 Dec 10;17(6):3224–39. doi: 10.1039/d5sc06553d (PMC12709544; doi:10.1039/d5sc06553d)
Supplement: SC-017-D5SC06553D-s001 [file SC-017-D5SC06553D-s001.pdf]

Supplementary Information (SI) for Chemical Science.
This journal is © The Royal Society of Chemistry 2025

Supporting Information

Uncovering Outer-Sphere Mechanisms Governing Chemoselectivity in Copper-Photocatalyzed ATRA Reactions of CF_3SO_2Cl with Alkenes

Farshad Shiri,[a] Morteza Jamshidi,[b] Saba Hadidi,*[c] Robert Stranger,[b] Alireza Ariafard*[b]

[a] Department of Chemistry, The Hong Kong University of Science and Technology, Kowloon, Hong Kong 999077, P. R. China

[b] Research School of Chemistry, Australian National University, Canberra, Australian Capital Territory 2601, Australia

[c] Inorganic Chemistry Department, Faculty of Chemistry, Razi University, Kermanshah 6714414971, Iran

Table of contents:

Figure S1. Free energy profiles comparing Cu(II)-assisted and direct pathways for in situ generation of the $^{\bullet}CF_3$ radical calculated at the SMD/wB97XD/def2-TZVP//SMD/B3LYP-D3/def2-SVP level in acetonitrile. (page S2)

Figure S2. Computed free energy profiles for Cu(II)-assisted formation of **P1(S2)** and **P2(S2)** via pathways A and B calculated at the SMD/wB97XD/def2-TZVP //SMD/B3LYP-D3/def2-SVP level in acetonitrile. (page S3)

CASSCF Analysis of the Electronic Structures of Key Intermediates. (page S4)

Figure S3. CASSCF analysis of selected radical intermediates **R$^{\bullet}$(S3)**, **F(S1)**, and **E(S1)**. (page S5)

Table S1. Cartesian coordinates along with total energies for all structures optimized in this study. (page S6)

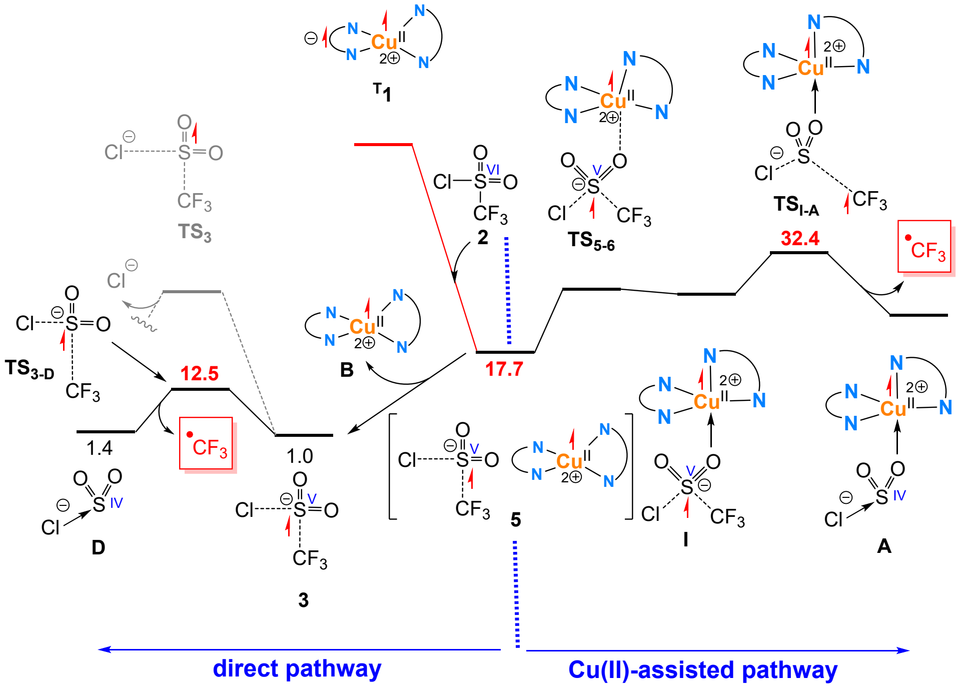

Figure S1. Free energy profiles comparing Cu(II)-assisted and direct pathways for in situ generation of the $^{\bullet}CF_3$ radical. Selected relative free energies are given in kcal/mol and calculated at the SMD/wB97XD/def2-TZVP//SMD/B3LYP-D3/def2-SVP level in acetonitrile.

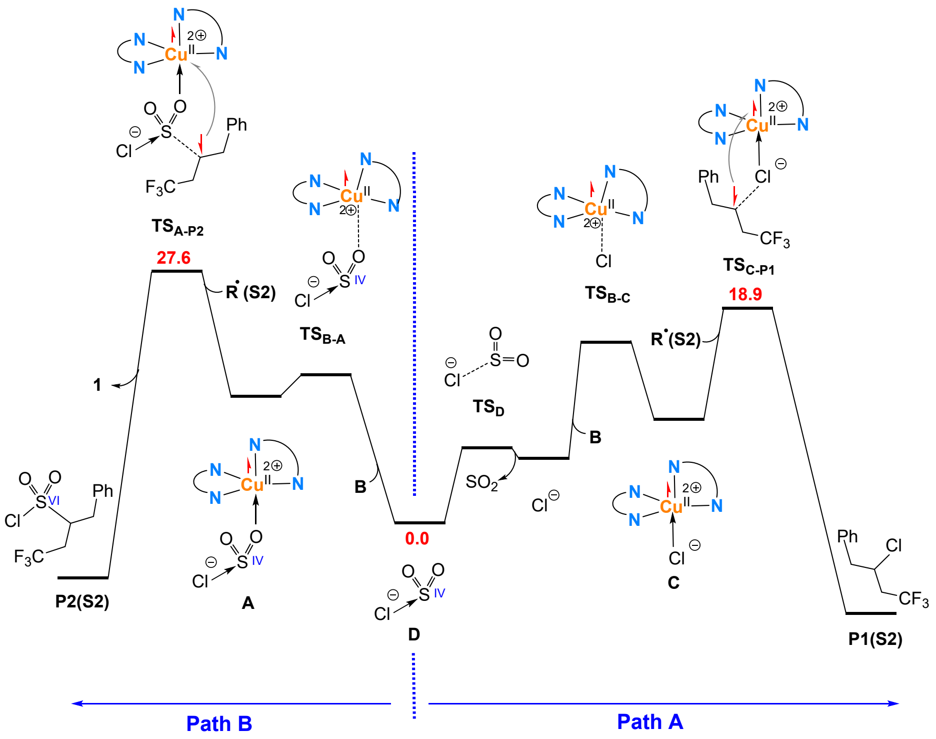

Figure S2. Computed free energy profiles for Cu(II)-assisted formation of **P1(S2)** and **P2(S2)** via pathways A and B. Selected relative free energies are given in kcal/mol and calculated at the SMD/wB97XD/def2-TZVP //SMD/B3LYP-D3/def2-SVP level in acetonitrile.

CASSCF Analysis of the Electronic Structures of Key Intermediates. We have performed CASSCF calculations on the key radical intermediates **R•(S3)**, **F(S1)**, and **E(S1)** to obtain a more accurate description of their electronic structures (Figure S3). For **F(S1)** and **E(S1)**, we employed a (5,4) active space comprising five electrons distributed across four orbitals corresponding to the σ and σ* orbitals of the C–S and S–Cl bonds to obtain a more detailed understanding of the electronic structures of these species. For **R•(S3)**, we constructed a (7,7) active space consisting of the six π electrons of the aromatic ring together with the singly occupied p orbital at the β-carbon radical centre to examine potential electronic interaction between the ring and the radical centre.

Because spin density is not rigorously defined for multiconfigurational wavefunctions, the distribution of unpaired electrons was examined through the odd-electron density (OED), computed using the Multiwfn 3.8 program.

The CASSCF natural orbital occupation numbers and odd electron density (OED) analysis provide important insights into the electronic structure of these intermediates:

- **E(S1)**: The odd-electron density is predominantly localized on the sulfur and chlorine atoms, with minimal contribution from the adjacent carbon. This distribution confirms that the unpaired electron primarily occupies the σ* antibonding orbital of the S–Cl bond, while the C–S bond retains single-bond character. Such an electronic configuration supports the notion that addition of the R• radical occurs at the sulfur atom of $[SO_2Cl]^-$, resulting in oxidation of S(IV) to S(V).
- **F(S1)**: The CASSCF calculations reveal that the doubly occupied σ orbital of the C–Cl bond is largely localized on Cl, whereas the singly occupied orbital is predominantly centred on C (Cl and C contributions of 0.015 and 0.72, respectively), confirming the presence of a highly polar 2c–3e bond.

- **R•(S3)**: the CASSCF results show that the singly occupied molecular orbital is completely localised on the β-carbon radical centre, with no participation from the aromatic ring, confirming that the radical is fully localised and does not interact with the π system of the ring.

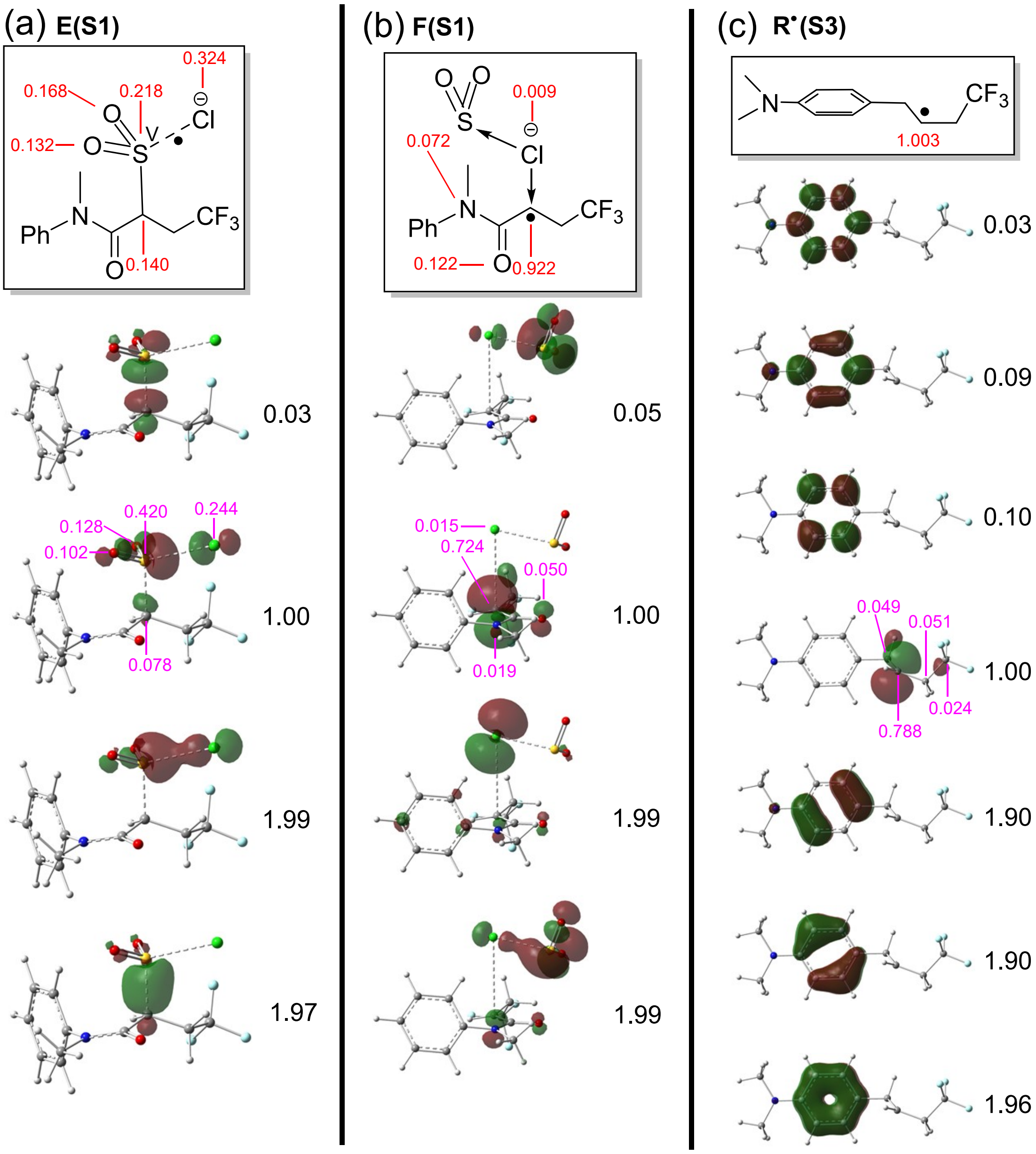

Figure S3. CASSCF analysis of selected radical intermediates **R•(S3)**, **F(S1)**, and **E(S1)**. Black numbers denote CASSCF natural-orbital occupation numbers, and pink values indicate the corresponding atomic contributions to the odd-electron density. Red values in the boxed structures represent the spin density values obtained from B3LYP-D3 calculations. The results show good agreement between the CASSCF and DFT spin distributions.

Table S1. Cartesian coordinates along with total potential (E), enthalpy (H) and Gibbs free energies (G) of all structures (page S2)

1
E (SMD/B3LYP-D3/def2-SVP) = -4164.250680 au
H (SMD/B3LYP-D3/def2-SVP) = -4163.399569 au
G (SMD/B3LYP-D3/def2-SVP) = -4163.538678 au
E (SMD/B3LYP-D3/def2-TZVP//SMD/B3LYP-D3/def2-SVP) = -4167.203621 au

C	-3.20923300	-4.50616100	-3.75541800
O	-2.33579600	-4.37051100	-2.64372100
C	-2.50178800	-3.33561900	-1.79244900
C	-1.60022600	-3.26668000	-0.71191600
C	-1.68704400	-2.23105700	0.20812000
C	-3.49901800	-2.35179900	-1.91835700
C	-3.58133500	-1.32289300	-0.97551900
C	-2.67542100	-1.23584700	0.09387900
C	-2.74487900	-0.10874500	1.05235800
C	-4.00460000	0.33896900	1.53130900
C	-4.07396400	1.43992700	2.35684200
C	-2.88784500	2.13296100	2.69346200
C	-1.66457900	1.61196900	2.19339400
N	-1.60821400	0.50233400	1.41026900
Cu	0.35739700	0.13947700	0.71312100
N	1.26938500	0.54273000	-1.13481900
C	2.24195500	-0.35605000	-1.41835800
C	2.37807100	-1.51659000	-0.55802000
N	1.59150900	-1.60258000	0.54639900
C	1.71697300	-2.65272100	1.36907100
C	0.84671100	-2.73915400	2.56758000
C	0.50609500	-1.59872800	3.32452200
C	-0.35930500	-1.68554400	4.40644800
C	-0.92328300	-2.92315500	4.77230600
C	-0.58227100	-4.07413500	4.03952800
C	0.29703400	-3.97232600	2.95910600
O	-1.76692000	-2.90718500	5.82513400
C	-2.38053100	-4.11705500	6.24691800
C	2.65703700	-3.68472300	1.09951400
C	3.46961700	-3.60429300	-0.01062100
C	3.35752800	-2.49324600	-0.87895100
C	4.21933400	-2.30616700	-2.01117300
C	4.13338800	-1.17983900	-2.77947500
C	3.14710600	-0.17721500	-2.49872000
C	3.03089900	1.01830400	-3.24761500
C	2.05382900	1.93711500	-2.92335300
C	1.15707800	1.66122700	-1.85974500
C	0.07059400	2.60322300	-1.50453400
C	-1.22008600	2.12734400	-1.22980200
C	-2.24040600	2.98284700	-0.81458800
C	-1.97447200	4.35348800	-0.65010500
C	-0.68902800	4.84743000	-0.94810600
C	0.31401900	3.98492400	-1.37825500
O	-2.87640600	5.25886500	-0.21172700
C	-4.19218800	4.82969100	0.10812400
N	0.71801600	1.86042800	1.93039900
C	-0.44005900	2.35338000	2.43273100
C	-0.50262700	3.59980500	3.11328500
C	-1.75239200	4.06987700	3.63779000
C	-2.90005800	3.35491700	3.44607100
C	0.69604500	4.34631900	3.20719900
C	1.85609500	3.84813000	2.65114700
C	1.84382600	2.57438500	2.02683600
C	3.08513700	2.00386600	1.45082200
C	3.88752300	2.75974700	0.57425000
C	5.01772100	2.20299200	-0.01518400
C	5.39411800	0.87780800	0.27793400
C	4.62283900	0.12795700	1.18184700
C	3.47615400	0.69041000	1.74418400
O	6.49766400	0.41584100	-0.35133400
C	6.98168900	-0.88225700	-0.03951300
H	-2.88792900	-5.40790700	-4.29427200
H	-3.14186100	-3.63845300	-4.43497000
H	-4.25876300	-4.63305900	-3.43666900
H	-0.83833400	-4.04341500	-0.61354800

H -0.99265300 -2.19421500 1.04362100
H -4.20804300 -2.37247000 -2.74582200
H -4.35247200 -0.55831700 -1.09888000
H -4.90791900 -0.20103500 1.24500300
H -5.03430700 1.79948200 2.73427000
H 0.92309800 -0.62725700 3.06044300
H -0.61892100 -0.79874500 4.98889000
H -1.00330600 -5.04706400 4.29274600
H 0.52518600 -4.87496100 2.38747000
H -3.01826500 -3.85888900 7.10327700
H -1.63258800 -4.86297000 6.56789200
H -3.00773100 -4.55460500 5.45055000
H 2.75192900 -4.51880300 1.79552200
H 4.21519900 -4.37647100 -0.21571200
H 4.96555200 -3.07461200 -2.22616700
H 4.81230000 -1.02128400 -3.62073000
H 3.71636500 1.19990600 -4.07915100
H 1.94081400 2.86045600 -3.49336900
H -1.42842900 1.06081900 -1.32713000
H -3.22452600 2.56864800 -0.59694300
H -0.49700300 5.91462100 -0.81682300
H 1.31191900 4.38156200 -1.58129700
H -4.73414400 5.72127200 0.45174900
H -4.19055700 4.07742200 0.91475700
H -4.70977700 4.41222300 -0.77338400
H -1.76580300 5.01932100 4.17840800
H -3.85676800 3.71737700 3.82941700
H 0.68802800 5.31414800 3.71472200
H 2.79159800 4.40671200 2.70772600
H 3.60263600 3.78539300 0.32677100
H 5.62600000 2.77462600 -0.71946800
H 4.89033300 -0.89750600 1.43377100
H 2.87195300 0.09142300 2.42513000
H 7.87591500 -1.03690400 -0.65870400
H 6.24172400 -1.66367000 -0.27864000
H 7.26193100 -0.96458100 1.02543200

^{s}**1***
E (SMD/B3LYP-D3/def2-SVP) = -4164.24646781 au
H (SMD/B3LYP-D3/def2-SVP) = -4163.3537380 au
G (SMD/B3LYP-D3/def2-SVP) = -4163.4884610 au
E (SMD/B3LYP-D3/def2-TZVP//SMD/B3LYP-D3/def2-SVP) = -4167.19813632 au
C -1.04905900 -4.85012600 -3.67443500
O -1.69780600 -5.07080700 -2.42862400
C -1.90193100 -4.03115100 -1.59594700
C -2.50838000 -4.33165800 -0.35924900
C -2.73294500 -3.33053400 0.57582400
C -1.55866900 -2.69911600 -1.88717200
C -1.78137400 -1.70665900 -0.93380400
C -2.34500100 -1.99887700 0.31886100
C -2.53396100 -0.94199900 1.33489300
C -3.72759600 -0.88946900 2.10884200
C -3.93830600 0.15496700 2.97941000
C -2.96740200 1.18002700 3.10383500
C -1.77883300 1.03141800 2.34587600
N -1.58397200 -0.01776800 1.50808600
Cu 0.30672100 0.11552600 0.60155800
N 1.15411400 0.47639800 -1.20395900
C 1.83984000 -0.64573800 -1.63804700
C 1.78167800 -1.80508000 -0.83919800
N 1.09799800 -1.73290400 0.36383000
C 1.01889500 -2.83035600 1.16079500
C 0.34586800 -2.68619500 2.47476500
C 0.53216900 -1.53511700 3.26827100
C -0.14142300 -1.36712300 4.47055800
C -1.03589600 -2.35318100 4.92789800
C -1.20604000 -3.52587500 4.17033300
C -0.51537600 -3.68114300 2.96553700
O -1.67732000 -2.08760900 6.08757200
C -2.61445000 -3.02591900 6.59395200
C 1.58010500 -4.04859400 0.76551800
C 2.28080900 -4.14666400 -0.45431900
C 2.41176800 -3.01744700 -1.26823900
C 3.14572300 -2.99548600 -2.50873300

C	3.24124400	-1.85727200	-3.26018600
C	2.59165700	-0.63532400	-2.85723100
C	2.64551400	0.55845700	-3.58315500
C	1.93456900	1.68661200	-3.12364400
C	1.18226500	1.61243400	-1.94773300
C	0.37161300	2.75945500	-1.47169300
C	-0.91940100	2.56754200	-0.95396500
C	-1.65042900	3.61190200	-0.38716400
C	-1.08806700	4.89751300	-0.32445200
C	0.17758500	5.12121600	-0.90194700
C	0.88895000	4.07082000	-1.46997300
O	-1.67647000	5.96094400	0.26691300
C	-2.91832800	5.78102800	0.93252300
N	0.41132600	1.86009500	1.76123700
C	-0.73782000	2.03232600	2.46115000
C	-0.95716200	3.16536100	3.28332500
C	-2.18050100	3.29379700	4.01921400
C	-3.14668100	2.32958800	3.94104700
C	0.07273800	4.13752700	3.32101200
C	1.23851700	3.93127900	2.62064600
C	1.40988100	2.74419100	1.85340600
C	2.69061800	2.47085900	1.16815400
C	3.41430200	3.50720500	0.54078500
C	4.58667500	3.24012000	-0.15282300
C	5.08905000	1.92512800	-0.22827100
C	4.40817900	0.89073800	0.43790900
C	3.22723300	1.17357000	1.12177000
O	6.21592000	1.75644500	-0.94726500
C	6.75191900	0.44908300	-1.10645100
H	-0.97180800	-5.83182600	-4.16102700
H	-0.03572300	-4.43517400	-3.53585000
H	-1.63165000	-4.17239800	-4.32234900
H	-2.77717800	-5.36893400	-0.14862500
H	-3.17511700	-3.59218000	1.53862300
H	-1.11155100	-2.42695800	-2.84260800
H	-1.50848100	-0.67968800	-1.17966700
H	-4.48365100	-1.66336600	1.97504600
H	-4.86085300	0.21748800	3.56167500
H	1.21611900	-0.75313800	2.93249100
H	-0.00027400	-0.46739800	5.07332800
H	-1.88633000	-4.31225800	4.49741400
H	-0.68865100	-4.58192000	2.37414000
H	-3.01347200	-2.59650600	7.52312500
H	-2.13930200	-3.99617300	6.82225000
H	-3.44742100	-3.19084800	5.88780900
H	1.51495700	-4.90682100	1.43519900
H	2.74187100	-5.09062700	-0.75465200
H	3.63615200	-3.91695100	-2.83445800
H	3.81113300	-1.85583600	-4.19339200
H	3.21790700	0.60413900	-4.51278300
H	1.92517300	2.61006200	-3.70394900
H	-1.36284100	1.57002600	-0.97081200
H	-2.63941000	3.40814300	0.02218700
H	0.59835800	6.12844000	-0.86111600
H	1.88671100	4.25679500	-1.87111200
H	-3.17782100	6.75283300	1.37424900
H	-2.84276600	5.02847600	1.73682700
H	-3.71779000	5.48097600	0.23246400
H	-2.31875600	4.17749100	4.64604700
H	-4.07647500	2.41912200	4.50726100
H	-0.06308600	5.03851300	3.92404400
H	2.05183400	4.65514100	2.67123100
H	3.03025800	4.52823900	0.55872600
H	5.12935200	4.03632200	-0.66669600
H	4.78129100	-0.13263100	0.42422100
H	2.71365700	0.35693600	1.63148400
H	7.63795700	0.55388200	-1.74697500
H	6.03011500	-0.22770800	-1.59558700
H	7.05706700	0.01410300	-0.13887600

^{T}**1***

E (SMD/B3LYP-D3/def2-SVP) = -4164.199251 au
H (SMD/B3LYP-D3/def2-SVP) = -4163.349732 au
G (SMD/B3LYP-D3/def2-SVP) = -4163.484431 au

E (SMD/B3LYP-D3/def2-TZVP//SMD/B3LYP-D3/def2-SVP) = -4167.153712 au

C	-1.01092400	-4.84845200	-3.69343800
O	-1.66385000	-5.07340000	-2.45070000
C	-1.87632400	-4.03528700	-1.61784300
C	-2.48752500	-4.33936200	-0.38434800
C	-2.71906700	-3.34021000	0.55124100
C	-1.53684800	-2.70161400	-1.90573000
C	-1.76631000	-1.71143800	-0.95166200
C	-2.33406700	-2.00653400	0.29871800
C	-2.52226700	-0.95518200	1.31992600
C	-3.70910700	-0.91406700	2.10231800
C	-3.92590600	0.12676600	2.97794400
C	-2.96130900	1.15711000	3.10125400
C	-1.77390700	1.01854100	2.33962600
N	-1.57519600	-0.02182500	1.48780100
Cu	0.31921200	0.10792200	0.60861900
N	1.15569800	0.48298200	-1.18454300
C	1.82146100	-0.64652000	-1.63294800
C	1.76365000	-1.80339800	-0.83438400
N	1.06762100	-1.73184100	0.36262500
C	1.00909300	-2.81845100	1.17562500
C	0.33903000	-2.67539300	2.49019500
C	0.53541800	-1.53006000	3.28950900
C	-0.13804600	-1.36243400	4.49202600
C	-1.04047100	-2.34411200	4.94318200
C	-1.21771700	-3.51312000	4.18103500
C	-0.52746000	-3.66803500	2.97616100
O	-1.68175700	-2.07909500	6.10255100
C	-2.62811000	-3.01171400	6.60281900
C	1.58246200	-4.03500000	0.78749900
C	2.27707000	-4.13896600	-0.43505700
C	2.39829000	-3.01447700	-1.25754900
C	3.12243300	-2.99554300	-2.50409700
C	3.20188300	-1.86317300	-3.26651100
C	2.55427500	-0.64085100	-2.86346900
C	2.59313300	0.54975100	-3.59577400
C	1.88718300	1.67662500	-3.13186900
C	1.15746300	1.60985700	-1.93709400
C	0.35896900	2.76286300	-1.45789900
C	-0.92950600	2.58146400	-0.93062600
C	-1.64883300	3.63397700	-0.36390900
C	-1.07597800	4.91553300	-0.31168100
C	0.18675400	5.12812300	-0.90012500
C	0.88619600	4.07011600	-1.46834000
O	-1.65183600	5.98581600	0.27813300
C	-2.89290900	5.82075700	0.94956900
N	0.41782000	1.83776100	1.76415400
C	-0.73857100	2.01974400	2.45536200
C	-0.96349600	3.15826500	3.26832900
C	-2.18731400	3.28137300	4.00484900
C	-3.14559800	2.30824500	3.93554600
C	0.06092300	4.13545400	3.30057200
C	1.22921800	3.92675100	2.60223500
C	1.41149700	2.73375000	1.84997700
C	2.69602600	2.46217800	1.17245800
C	3.41618300	3.49827900	0.54000500
C	4.58795400	3.23231600	-0.15509100
C	5.09376200	1.91838600	-0.22778000
C	4.41764200	0.88460400	0.44407300
C	3.23763700	1.16659100	1.12992600
O	6.21864300	1.75002900	-0.95015100
C	6.75494400	0.44285100	-1.10928400
H	-0.92706000	-5.82924600	-4.18080600
H	-0.00002500	-4.42898300	-3.55047200
H	-1.59385000	-4.17270200	-4.34315500
H	-2.75370700	-5.37783300	-0.17619900
H	-3.16363900	-3.60553000	1.51192000
H	-1.08603400	-2.42669300	-2.85868500
H	-1.49375900	-0.68354000	-1.19400400
H	-4.46165900	-1.69142400	1.96773900
H	-4.84747700	0.18100800	3.56251900
H	1.22473500	-0.75197000	2.95472500
H	0.00959600	-0.46698800	5.09951800
H	-1.90317500	-4.29632800	4.50477500
H	-0.70493600	-4.56536500	2.38088400

H	-3.02630600	-2.58226700	7.53230400
H	-2.16139400	-3.98649300	6.82900000
H	-3.46012400	-3.16710500	5.89348600
H	1.52179900	-4.88853400	1.46361400
H	2.74096900	-5.08295200	-0.73124100
H	3.61568700	-3.91630400	-2.82740700
H	3.75989000	-1.86633300	-4.20679600
H	3.15174200	0.59249400	-4.53409800
H	1.86788600	2.59895100	-3.71354000
H	-1.37889700	1.58640600	-0.93992700
H	-2.63660700	3.43987000	0.05283800
H	0.61452700	6.13263700	-0.86755600
H	1.88181000	4.24671600	-1.87886000
H	-3.14233400	6.79766800	1.38570500
H	-2.82046600	5.07295200	1.75837900
H	-3.69710400	5.52296600	0.25407000
H	-2.33155400	4.16749200	4.62703700
H	-4.07409000	2.39324500	4.50475300
H	-0.07899000	5.04082700	3.89595200
H	2.03960500	4.65427600	2.64985100
H	3.02890500	4.51814500	0.55400000
H	5.12701700	4.02837400	-0.67302200
H	4.79350800	-0.13786100	0.43314900
H	2.72645100	0.34992100	1.64262100
H	7.63769600	0.54691300	-1.75445300
H	6.03108400	-0.23555400	-1.59329900
H	7.06529000	0.01008800	-0.14235400

2

E (SMD/B3LYP-D3/def2-SVP) = -1345.767427 au
H (SMD/B3LYP-D3/def2-SVP) = -1345.733458 au
G (SMD/B3LYP-D3/def2-SVP) = -1345.776480 au
E (SMD/B3LYP-D3/def2-TZVP//SMD/B3LYP-D3/def2-SVP) = -1346.716906 au

S	-0.12689800	-2.18247200	-3.49587100
O	1.23510600	-2.14521100	-3.99864400
O	-0.64142200	-1.23436400	-2.52352000
C	-1.30219800	-2.16357400	-5.00135000
F	-0.92771200	-3.10145300	-5.84640500
F	-1.19364900	-0.96904900	-5.55193600
F	-2.53395200	-2.37286400	-4.58508800
Cl	-0.54047000	-4.13761200	-2.81378900

3

E (SMD/B3LYP-D3/def2-SVP) = -4164.094171 au
H (SMD/B3LYP-D3/def2-SVP) = -4163.240293 au
G (SMD/B3LYP-D3/def2-SVP) = -4163.372198 au
E (SMD/B3LYP-D3/def2-TZVP//SMD/B3LYP-D3/def2-SVP) = -4167.047543 au

C	-0.61834800	-4.75237400	-3.58732900
O	-1.32544700	-5.01202500	-2.38009100
C	-1.68021900	-3.98114200	-1.58993100
C	-2.31379800	-4.31229800	-0.37390300
C	-2.65013200	-3.32051400	0.53711300
C	-1.45734700	-2.62882900	-1.90340300
C	-1.79041900	-1.64587300	-0.97412200
C	-2.34645000	-1.96860000	0.27468400
C	-2.55892600	-0.92718800	1.29902200
C	-3.74737100	-0.88906100	2.07664500
C	-3.96489200	0.14667900	2.95945300
C	-2.99894300	1.17531100	3.09424600
C	-1.80945600	1.03916800	2.33792300
N	-1.60972000	0.00223600	1.48165600
Cu	0.25986400	0.13377200	0.62547400
N	1.13097700	0.48295900	-1.20153800
C	1.75775400	-0.64044500	-1.64180300
C	1.66472300	-1.83116800	-0.83614400
N	1.01102900	-1.75335200	0.35451100
C	0.91061400	-2.82615800	1.15300500
C	0.28556000	-2.68664900	2.48393000
C	0.51087400	-1.54593700	3.28389400
C	-0.12514600	-1.38656800	4.50527900
C	-1.01710500	-2.37103900	4.97494100
C	-1.21922700	-3.53626100	4.21106700
C	-0.56289000	-3.68698700	2.98948500

O	-1.61849300	-2.11546600	6.15165000
C	-2.54797600	-3.05192900	6.68234300
C	1.41902100	-4.08003900	0.71500200
C	2.07193300	-4.18387500	-0.49339000
C	2.24045500	-3.03556200	-1.30588400
C	2.94975400	-3.04490800	-2.55214700
C	3.07715200	-1.90269500	-3.29503400
C	2.48118100	-0.67353900	-2.85833900
C	2.56303300	0.53934400	-3.58737500
C	1.90779900	1.66222300	-3.12993400
C	1.14834800	1.60765500	-1.93047000
C	0.36345500	2.76744500	-1.46359900
C	-0.93210700	2.59920600	-0.94664500
C	-1.63539300	3.65867600	-0.37761200
C	-1.03795800	4.92987400	-0.31171300
C	0.22884000	5.12874900	-0.89908100
C	0.91169900	4.06655200	-1.47515100
O	-1.59416400	6.00109300	0.28483900
C	-2.84215300	5.85768700	0.95280300
N	0.38184200	1.84541300	1.76296400
C	-0.77290400	2.03289400	2.45713900
C	-0.99009700	3.16929200	3.27245200
C	-2.21473200	3.29295900	4.00795300
C	-3.17817200	2.32442600	3.93279500
C	0.04189700	4.13911700	3.30554900
C	1.20884200	3.92333900	2.60668500
C	1.38438300	2.73240700	1.84964100
C	2.66313300	2.45039000	1.16809900
C	3.39463100	3.48046800	0.53781600
C	4.55626800	3.20053800	-0.16731900
C	5.04215000	1.87891700	-0.25200300
C	4.35694200	0.85132300	0.42038600
C	3.18737500	1.14772300	1.11742400
O	6.15661300	1.69853300	-0.98465400
C	6.68201000	0.38633200	-1.14482900
H	-0.39449700	-5.73187900	-4.03076000
H	0.32765500	-4.21774700	-3.39419000
H	-1.22789000	-4.16720700	-4.29726500
H	-2.50203200	-5.36525000	-0.15425000
H	-3.10116800	-3.60058200	1.49046500
H	-1.00884100	-2.33531700	-2.85157000
H	-1.59028000	-0.60108200	-1.22184800
H	-4.49821400	-1.66665800	1.93272900
H	-4.88832100	0.19812600	3.54119900
H	1.19550400	-0.76896800	2.93729700
H	0.04646200	-0.49867900	5.11697200
H	-1.89447800	-4.32093000	4.55152800
H	-0.75689500	-4.58434400	2.40020800
H	-2.91023500	-2.62573200	7.62748000
H	-2.07150600	-4.02651000	6.88529400
H	-3.40409400	-3.20130700	6.00185300
H	1.31838300	-4.94793000	1.36674300
H	2.48063900	-5.14124100	-0.82488200
H	3.39084000	-3.98518400	-2.89013800
H	3.62532400	-1.90656400	-4.23973000
H	3.12901000	0.56638500	-4.52154900
H	1.92967500	2.59247900	-3.69846300
H	-1.39889300	1.61216700	-0.97154700
H	-2.63000200	3.48009500	0.02901700
H	0.67045000	6.12671900	-0.86033500
H	1.90856000	4.23218700	-1.88684600
H	-3.06912000	6.83641700	1.39632700
H	-2.78636600	5.10139300	1.75439600
H	-3.64899600	5.58453300	0.25105000
H	-2.35612600	4.17642000	4.63435300
H	-4.10709300	2.41241500	4.50062100
H	-0.09064500	5.04444700	3.90249100
H	2.02462300	4.64466700	2.65555500
H	3.02296800	4.50581700	0.56144100
H	5.10422600	3.99153200	-0.68352700
H	4.72049900	-0.17551500	0.40506400
H	2.66971200	0.33725700	1.63479200
H	7.55974700	0.48227900	-1.79778400
H	5.94885800	-0.28764000	-1.62074600
H	6.99674400	-0.04511000	-0.17893500

4
E (SMD/B3LYP-D3/def2-SVP) = -1345.923331 au
H (SMD/B3LYP-D3/def2-SVP) = -1345.890909 au
G (SMD/B3LYP-D3/def2-SVP) = -1345.937010 au
E (SMD/B3LYP-D3/def2-TZVP//SMD/B3LYP-D3/def2-SVP) = -1346.876683 au

S	-0.04689100	-2.11312900	-3.43372400
O	1.26312700	-1.88519300	-4.12121500
O	-0.62582900	-0.98598600	-2.63680300
C	-1.24549200	-2.16710300	-4.92635500
F	-0.90011700	-3.12594000	-5.78500700
F	-1.19588000	-0.99180500	-5.56830900
F	-2.50023400	-2.37157100	-4.52688000
Cl	-0.77988100	-4.66587200	-2.818308001

D
E (SMD/B3LYP-D3/def2-SVP) = -1008.601853 au
H (SMD/B3LYP-D3/def2-SVP) = -1008.588188 au
G (SMD/B3LYP-D3/def2-SVP) = -1008.622551 au
E (SMD/B3LYP-D3/def2-TZVP//SMD/B3LYP-D3/def2-SVP) = -1009.143238 au

S	-0.14120500	-1.73967100	-3.77732900
O	1.31631600	-1.75184100	-4.06681100
O	-0.52175800	-0.88166100	-2.62521900
Cl	-0.51008500	-4.09313900	-2.82591000

TS_{3-D}
E (SMD/B3LYP-D3/def2-SVP) = -1345.905863 au
H (SMD/B3LYP-D3/def2-SVP) = -1345.874958 au
G (SMD/B3LYP-D3/def2-SVP) = -1345.926942 au
E (SMD/B3LYP-D3/def2-TZVP//SMD/B3LYP-D3/def2-SVP) = -1346.860800 au

S	-0.09478000	-2.23557600	-3.36635700
O	1.30444300	-2.25117600	-3.86114600
O	-0.32930900	-1.31195300	-2.22857600
C	-1.86711300	-1.95509400	-5.80038200
F	-1.34680100	-2.94701500	-6.50283300
F	-1.97234200	-0.86057100	-6.53714000
F	-3.04061300	-2.29768000	-5.29674300
Cl	-0.33239400	-4.51816400	-2.28295000

TS_3
E (SMD/B3LYP-D3/def2-SVP) = -1345.887954 au
H (SMD/B3LYP-D3/def2-SVP) = -1345.857013 au
G (SMD/B3LYP-D3/def2-SVP) = -1345.906724 au
E (SMD/B3LYP-D3/def2-TZVP//SMD/B3LYP-D3/def2-SVP) = -1346.850285 au

S	0.11621100	-1.94548100	-3.60099300
O	1.30797000	-1.43436300	-4.35261000
O	-0.57477300	-1.01703300	-2.64831700
C	-1.20328300	-2.05464800	-5.03110000
F	-0.76184700	-2.87158500	-5.98294300
F	-1.40625900	-0.84703300	-5.55142400
F	-2.35021800	-2.51863800	-4.54429900
Cl	-1.55219400	-7.24964300	-1.62846500

5
E (SMD/B3LYP-D3/def2-SVP) = -5510.037951 au
H (SMD/B3LYP-D3/def2-SVP) = -5509.150513 au
G (SMD/B3LYP-D3/def2-SVP) = -5509.311120 au
E (SMD/B3LYP-D3/def2-TZVP//SMD/B3LYP-D3/def2-SVP) = -5513.926071 au

C	-3.45910000	-4.47041900	-3.55841700
O	-2.48730700	-4.29791500	-2.53555400
C	-2.62930000	-3.29531600	-1.64577300
C	-1.68229400	-3.24596500	-0.60491000
C	-1.73767300	-2.23473700	0.34182800
C	-3.65111800	-2.33016300	-1.69303800
C	-3.70006700	-1.32468500	-0.72713500
C	-2.73693700	-1.24867100	0.29379100
C	-2.75858200	-0.13071100	1.25525100
C	-3.99582900	0.31660500	1.79638500
C	-4.03505500	1.41628000	2.62160900
C	-2.84345900	2.13411400	2.88501400

C	-1.64751800	1.62613600	2.32051200
N	-1.61397600	0.49211300	1.56724300
Cu	0.33846500	0.21548900	0.77276500
N	1.32723700	0.61702600	-0.91397000
C	2.20796100	-0.34773200	-1.26790800
C	2.30230600	-1.51402000	-0.42988700
N	1.52163500	-1.56891300	0.68079000
C	1.61547500	-2.60849700	1.51998000
C	0.76612900	-2.63784400	2.73088100
C	0.47290200	-1.46830600	3.46573200
C	-0.38204800	-1.50121300	4.55740900
C	-0.98082100	-2.71218900	4.95768000
C	-0.68460600	-3.89161400	4.24910700
C	0.18324900	-3.84480400	3.15793600
O	-1.80925800	-2.64513600	6.01704900
C	-2.45556300	-3.82522000	6.47714500
C	2.50819900	-3.67853200	1.23234500
C	3.28862800	-3.64817300	0.09638700
C	3.21225800	-2.53861900	-0.78045400
C	4.01533700	-2.39999300	-1.96253000
C	3.91925900	-1.28929300	-2.75467100
C	3.01421600	-0.22832700	-2.42128900
C	2.85871300	0.94761400	-3.19536500
C	1.94128400	1.90784600	-2.81694700
C	1.14782100	1.70606900	-1.66340400
C	0.07196600	2.63720400	-1.25417800
C	-1.24959900	2.17036400	-1.16864300
C	-2.27176600	2.98951400	-0.68644100
C	-1.97403600	4.29707800	-0.26604900
C	-0.65609900	4.78291900	-0.38900300
C	0.35129300	3.96566300	-0.88727000
O	-2.87272300	5.15246100	0.26602300
C	-4.22340300	4.73962200	0.41811200
N	0.69220500	1.92862800	1.85899300
C	-0.43374800	2.39349900	2.45876500
C	-0.45593800	3.62946500	3.15358000
C	-1.67508500	4.08691000	3.75271100
C	-2.82611900	3.36184200	3.62563100
C	0.74697600	4.37540700	3.18270700
C	1.87680100	3.88050200	2.56711500
C	1.83622200	2.61972100	1.92040500
C	3.05759500	2.03933200	1.32160200
C	3.81465500	2.76045500	0.37823100
C	4.93110000	2.18763600	-0.21673900
C	5.34079600	0.88604300	0.13778000
C	4.61899700	0.17793900	1.11316800
C	3.48216500	0.75362900	1.68233500
O	6.42413800	0.40792600	-0.50835400
C	6.91797800	-0.88373300	-0.18138700
H	-3.13265500	-5.33560900	-4.15121900
H	-3.52309400	-3.59037900	-4.21628400
H	-4.45648700	-4.67998400	-3.13334200
H	-0.90641200	-4.01361800	-0.56430000
H	-1.01804200	-2.21583700	1.15515300
H	-4.39617000	-2.34083000	-2.48768600
H	-4.48375700	-0.56632000	-0.79610600
H	-4.90592600	-0.23422600	1.55801500
H	-4.97807600	1.76107400	3.05222000
H	0.93049300	-0.51742100	3.18614400
H	-0.60440600	-0.59401000	5.12314700
H	-1.13379100	-4.84390900	4.53069400
H	0.37414100	-4.76638100	2.60371300
H	-3.07336400	-3.52495600	7.33412400
H	-1.72632700	-4.58516500	6.80713800
H	-3.10567500	-4.26111000	5.69882100
H	2.58260600	-4.51065200	1.93276600
H	3.98150600	-4.46350700	-0.12515200
H	4.70730400	-3.20675700	-2.21417400
H	4.52134200	-1.19147300	-3.65921600
H	3.44961600	1.07022600	-4.10404600
H	1.77772900	2.80341300	-3.41661900
H	-1.47122100	1.14858700	-1.48198800
H	-3.28394900	2.59169700	-0.61827200
H	-0.44505800	5.80342300	-0.06267400
H	1.37437600	4.34310400	-0.95099600

H	-4.75111000	5.57638100	0.89529500
H	-4.30725600	3.84875300	1.06146600
H	-4.69379700	4.52560000	-0.55757600
H	-1.66410000	5.03523900	4.29435700
H	-3.76342800	3.71689300	4.05955000
H	0.76738500	5.33940900	3.69657100
H	2.81861800	4.43010200	2.58866600
H	3.49990300	3.76374500	0.08253300
H	5.50281400	2.72482300	-0.97629200
H	4.91492600	-0.82592200	1.41512400
H	2.91005300	0.18701900	2.41820600
H	7.78863000	-1.05487200	-0.82885000
H	6.16714500	-1.66736900	-0.37584900
H	7.23655400	-0.94012100	0.87408600
C	-1.51251500	-1.43848300	-5.07200200
F	-1.47671700	-1.11139300	-6.36155600
F	-1.20507600	-2.72737300	-4.93665100
F	-2.76315500	-1.25230400	-4.62576300
S	-0.31581500	-0.34999900	-4.05358700
O	-0.47958200	-0.94147700	-2.68544200
O	-0.88459200	1.01470100	-4.28819000
Cl	1.75234500	-1.21585100	-5.60520400

TS$_{5-6}$
E (SMD/B3LYP-D3/def2-SVP) = -5510.037398 au
H (SMD/B3LYP-D3/def2-SVP) = -5509.150607 au
G (SMD/B3LYP-D3/def2-SVP) = -5509.306258 au
E (SMD/B3LYP-D3/def2-TZVP//SMD/B3LYP-D3/def2-SVP) = -5513.921355 au

C	-3.94802500	-5.08272400	-2.76191600
O	-2.92370900	-4.77781900	-1.82656300
C	-2.95997200	-3.60842500	-1.15701900
C	-1.91043900	-3.37465500	-0.24952700
C	-1.86935900	-2.19631300	0.47664700
C	-3.96527000	-2.63607000	-1.31099900
C	-3.91588000	-1.46323000	-0.55906700
C	-2.86851700	-1.21981900	0.34741700
C	-2.82678100	0.02423400	1.13643500
C	-4.05327900	0.58093600	1.60276800
C	-4.06104200	1.75035300	2.32167200
C	-2.84073300	2.42083900	2.57434500
C	-1.65545000	1.81005800	2.09193700
N	-1.65748400	0.62759700	1.40946000
Cu	0.30859900	0.30765600	0.58129500
N	1.36030400	0.97128700	-1.00904100
C	2.31445900	0.09801500	-1.41602100
C	2.40094000	-1.17074800	-0.74513700
N	1.55698600	-1.40403000	0.29518200
C	1.69105800	-2.52391200	1.02010400
C	0.85122200	-2.73183600	2.22041800
C	0.50064700	-1.66856900	3.08047600
C	-0.35118000	-1.86349500	4.15743500
C	-0.88686300	-3.13979400	4.42002200
C	-0.52598300	-4.21802400	3.59085600
C	0.33767900	-4.00778700	2.51573900
O	-1.72152300	-3.23039000	5.47270500
C	-2.32263700	-4.48041200	5.78628500
C	2.64968700	-3.50893200	0.65126300
C	3.47632900	-3.30447700	-0.42944300
C	3.39153200	-2.09351300	-1.15400500
C	4.27369600	-1.76465800	-2.23270200
C	4.20048600	-0.54986800	-2.85511800
C	3.22223900	0.41668200	-2.45463100
C	3.11466200	1.70480400	-3.03082200
C	2.12800400	2.56721400	-2.60526500
C	1.21635900	2.16115900	-1.59907500
C	0.08129300	3.02035600	-1.19926500
C	-1.22682100	2.51031400	-1.21588800
C	-2.30901900	3.29149000	-0.80791800
C	-2.08960000	4.60395100	-0.35660500
C	-0.78219600	5.13145800	-0.36824800
C	0.28593800	4.35392600	-0.79656400
O	-3.05662600	5.42551800	0.10268100
C	-4.40380700	4.97499900	0.11853700
N	0.72379700	1.95286400	1.76123400

C	-0.40613800	2.51260300	2.26395700
C	-0.38811000	3.78000700	2.90274600
C	-1.60488700	4.34886100	3.39784300
C	-2.78958200	3.68877300	3.23860200
C	0.85845900	4.44479700	2.98833200
C	1.99175800	3.84638500	2.48395800
C	1.90863100	2.56510700	1.88182900
C	3.13390900	1.89034000	1.40165800
C	4.03986400	2.55699700	0.55232000
C	5.17497100	1.91022300	0.08263900
C	5.45429800	0.58417800	0.47119100
C	4.58410300	-0.07098900	1.35811400
C	3.43233700	0.58014700	1.80046700
O	6.56631000	0.03127800	-0.05518700
C	6.91880300	-1.29841800	0.30154400
H	-3.70187300	-6.06867000	-3.17935700
H	-3.98364700	-4.34507900	-3.58039800
H	-4.93865600	-5.13527700	-2.27693700
H	-1.12404000	-4.12503300	-0.14812400
H	-1.05765500	-2.02387800	1.17442500
H	-4.77740700	-2.77758300	-2.02361000
H	-4.69693800	-0.71491600	-0.71151900
H	-4.98475300	0.05225300	1.40267400
H	-4.99719500	2.17675300	2.68932000
H	0.89008600	-0.66556700	2.90017600
H	-0.62301300	-1.03441400	4.81408600
H	-0.92306000	-5.21785600	3.76455300
H	0.57345200	-4.85297700	1.86573000
H	-2.96099600	-4.30454400	6.66258000
H	-1.56612700	-5.24335600	6.03869000
H	-2.94623600	-4.84968900	4.95373500
H	2.74159800	-4.41263900	1.25387000
H	4.22129700	-4.05151900	-0.71264700
H	5.02119800	-2.50234500	-2.53209200
H	4.88578300	-0.29101800	-3.66511800
H	3.80981100	1.99795800	-3.82106500
H	2.00534500	3.55281300	-3.05592900
H	-1.39106900	1.48349200	-1.55022300
H	-3.31010800	2.86175700	-0.82412700
H	-0.63043500	6.15442100	-0.01769200
H	1.29634900	4.76894000	-0.78432300
H	-4.99912300	5.79412300	0.54403700
H	-4.52663400	4.07752000	0.74654700
H	-4.76859200	4.75497600	-0.90008800
H	-1.56317400	5.32442000	3.88688500
H	-3.72661900	4.12322600	3.59369100
H	0.90867200	5.42889800	3.46037800
H	2.96614900	4.33012900	2.55987000
H	3.83079300	3.57932700	0.22980500
H	5.86098000	2.40643600	-0.60686000
H	4.77662100	-1.09142500	1.68701300
H	2.75644300	0.05755500	2.47636200
H	7.83299800	-1.53708500	-0.25848200
H	6.13003900	-2.01733900	0.02541800
H	7.12536500	-1.38590100	1.38263600
C	-1.59904200	-1.65101400	-4.30080200
F	-1.17312800	-1.76213600	-5.55840600
F	-2.04787600	-2.83497100	-3.89223400
F	-2.61157900	-0.77882800	-4.26436300
S	-0.18048600	-1.00411000	-3.18837000
O	-0.92143300	-0.70903600	-1.91671300
O	0.29702200	0.17125300	-3.98463700
Cl	1.03331400	-3.42535400	-3.20900700

I

E (SMD/B3LYP-D3/def2-SVP) = -5510.041147 au
H (SMD/B3LYP-D3/def2-SVP) = -5509.153475 au
G (SMD/B3LYP-D3/def2-SVP) = -5509.309192 au
E (SMD/B3LYP-D3/def2-TZVP//SMD/B3LYP-D3/def2-SVP) = -5513.915686 au

C	-3.04872000	-5.40190200	-2.93239200
O	-2.58096100	-5.21427400	-1.60474900
C	-2.77997400	-4.02056400	-1.00974900
C	-2.00007000	-3.74844100	0.12809100
C	-2.09227900	-2.51603400	0.75548700

C	-3.70807400	-3.06154000	-1.45319900
C	-3.81711900	-1.83986500	-0.78808000
C	-2.98467400	-1.53320500	0.29796700
C	-3.01129200	-0.19867100	0.92499600
C	-4.26150300	0.38610600	1.26431900
C	-4.30921700	1.59251800	1.92287600
C	-3.10347900	2.25588900	2.25279000
C	-1.89243900	1.64054500	1.84594100
N	-1.86254500	0.44431900	1.19204600
Cu	0.01803600	0.26096600	0.24930100
N	1.39766800	0.93738900	-1.11548300
C	2.32358700	0.02473600	-1.49794700
C	2.37897400	-1.25077100	-0.81448000
N	1.55124100	-1.46338300	0.23650200
C	1.67383800	-2.57318300	0.97079800
C	0.86715100	-2.72515900	2.20330100
C	0.44653400	-1.60428100	2.95110100
C	-0.33512500	-1.74336500	4.08863700
C	-0.73731900	-3.02120700	4.52441100
C	-0.31790500	-4.15229400	3.80101000
C	0.47930900	-3.99543500	2.66597600
O	-1.51068100	-3.06095800	5.62729300
C	-1.96857500	-4.31479900	6.11656500
C	2.60603000	-3.58519500	0.60241800
C	3.42101500	-3.40511100	-0.49114900
C	3.35105000	-2.19851100	-1.22631100
C	4.24888100	-1.89890600	-2.30130900
C	4.21494400	-0.68214900	-2.92045100
C	3.26088500	0.31059700	-2.52460200
C	3.21801500	1.59919600	-3.10460300
C	2.28018600	2.51359800	-2.67754400
C	1.34072500	2.14474100	-1.68505300
C	0.28327500	3.09830500	-1.26637100
C	-1.07353800	2.75871800	-1.35660800
C	-2.07507100	3.63225900	-0.93003400
C	-1.72278900	4.87925000	-0.38841300
C	-0.36338400	5.24501700	-0.32842200
C	0.62278100	4.37200700	-0.77167500
O	-2.60474000	5.77978800	0.09596000
C	-3.99409800	5.49131400	0.03592800
N	0.50614300	1.75333100	1.64504500
C	-0.64536500	2.32002300	2.09971600
C	-0.65479500	3.56565700	2.77939700
C	-1.89667500	4.14773300	3.19286400
C	-3.07898600	3.51598000	2.93489300
C	0.59277600	4.19607500	2.99422900
C	1.74662400	3.58998900	2.55151200
C	1.68629000	2.34470200	1.87467400
C	2.94942100	1.72086000	1.41440700
C	3.81304500	2.43865800	0.56256200
C	4.98105400	1.85993900	0.08587800
C	5.33681400	0.55205900	0.47203200
C	4.51968600	-0.14348000	1.37716100
C	3.33435100	0.44065700	1.82856500
O	6.46689600	0.05593100	-0.07500900
C	6.86758800	-1.26929600	0.24285500
H	-2.64922200	-6.36849400	-3.26974900
H	-2.67434300	-4.60462700	-3.59672500
H	-4.15125300	-5.43764500	-2.98223600
H	-1.29213100	-4.50385700	0.47257900
H	-1.46258700	-2.29589100	1.61145900
H	-4.32712200	-3.24788000	-2.32981300
H	-4.51674400	-1.09246800	-1.16664400
H	-5.17589900	-0.15733900	1.02410400
H	-5.26490200	2.04212200	2.20277300
H	0.72002600	-0.60357500	2.62711600
H	-0.65819700	-0.86810500	4.65630700
H	-0.61409000	-5.15578400	4.10570900
H	0.76531700	-4.89105500	2.11127700
H	-2.57852400	-4.09648900	7.00356600
H	-1.12854600	-4.96841300	6.40913900
H	-2.59166700	-4.83846300	5.37074400
H	2.69653400	-4.48508700	1.21023500
H	4.15168000	-4.16632100	-0.77502500
H	4.97946800	-2.65658900	-2.59349000

H	4.91638000	-0.43802500	-3.72136300
H	3.93351400	1.85718300	-3.88911400
H	2.22271900	3.51190900	-3.11285500
H	-1.35494300	1.78601600	-1.75196800
H	-3.11607000	3.32154200	-1.01200300
H	-0.10428800	6.21893500	0.09206400
H	1.67326600	4.66316200	-0.70089200
H	-4.50818100	6.34335800	0.50099700
H	-4.24594700	4.57324400	0.59254200
H	-4.34034100	5.38604000	-1.00721700
H	-1.87292900	5.11215000	3.70448900
H	-4.03060500	3.96126100	3.23281100
H	0.62670000	5.15862200	3.50993500
H	2.72239300	4.04742900	2.71795400
H	3.53740200	3.44418100	0.23749700
H	5.62995400	2.39298600	-0.61195900
H	4.77738300	-1.14739300	1.71256700
H	2.70611000	-0.11568100	2.52189500
H	7.77403700	-1.46848600	-0.34475400
H	6.09280000	-2.00494600	-0.03047400
H	7.10424400	-1.37550800	1.31607600
C	-2.53913900	-0.25389500	-3.60043800
F	-2.30928500	0.46285300	-4.69263800
F	-3.30460400	-1.29621100	-3.91513500
F	-3.17537900	0.50291900	-2.70778300
S	-0.79417500	-0.89111400	-2.88351100
O	-0.97563500	-0.51948200	-1.42191200
O	0.15733400	-0.05119200	-3.65304500
Cl	0.14579400	-3.35049000	-2.90279100

$TS_{I\text{-}A}$

E (SMD/B3LYP-D3/def2-SVP) = -5510.034838 au
H (SMD/B3LYP-D3/def2-SVP) = -5509.148827 au
G (SMD/B3LYP-D3/def2-SVP) = -5509.305415 au
E (SMD/B3LYP-D3/def2-TZVP//SMD/B3LYP-D3/def2-SVP) = -5513.908891 au

C	-2.93869800	-5.29057300	-3.07840000
O	-2.46099000	-5.12821700	-1.75191600
C	-2.70317000	-3.96811400	-1.10835600
C	-1.94488500	-3.72765900	0.05157900
C	-2.07727900	-2.52892100	0.73262600
C	-3.65601900	-3.01770900	-1.51811700
C	-3.80991800	-1.83391900	-0.79431400
C	-2.99090100	-1.55096600	0.30768200
C	-3.02016700	-0.22444100	0.95126200
C	-4.26764600	0.36015000	1.29917800
C	-4.30924200	1.57155400	1.94895600
C	-3.10064200	2.23880500	2.26062700
C	-1.89233100	1.61923600	1.85077500
N	-1.86877600	0.41885400	1.20597500
Cu	0.01914200	0.21703100	0.25605600
N	1.38882600	0.89728300	-1.13495000
C	2.31812200	-0.01598500	-1.50553400
C	2.39827700	-1.27125300	-0.78645200
N	1.57662700	-1.47199900	0.27047100
C	1.70387000	-2.57236200	1.01779300
C	0.87908900	-2.72225900	2.23861500
C	0.44715400	-1.60000600	2.97688300
C	-0.35486500	-1.73666700	4.10059300
C	-0.76497700	-3.01353900	4.53152000
C	-0.33520900	-4.14615500	3.81660300
C	0.48104900	-3.99166100	2.69459100
O	-1.55590300	-3.05078700	5.62237700
C	-2.01766700	-4.30365900	6.10994200
C	2.64950900	-3.57860100	0.67141600
C	3.46624900	-3.40651000	-0.42259200
C	3.37979200	-2.21658000	-1.18149200
C	4.26240400	-1.93557400	-2.27383300
C	4.20274000	-0.73965000	-2.93000000
C	3.24029800	0.25197900	-2.55107100
C	3.18009700	1.52599000	-3.16144600
C	2.24457200	2.44555900	-2.74007700
C	1.32136400	2.09281500	-1.72658300
C	0.26545500	3.04937700	-1.31210900
C	-1.08771700	2.69223400	-1.36679700

C	-2.09370800	3.56440700	-0.94933800
C	-1.74741800	4.82982900	-0.44837800
C	-0.39135200	5.21212000	-0.41977000
C	0.59837900	4.33914500	-0.85640800
O	-2.63289700	5.73318100	0.02464500
C	-4.01962000	5.42816700	-0.01089000
N	0.50773300	1.72764900	1.63564800
C	-0.64235200	2.30145300	2.08707200
C	-0.64926800	3.55888900	2.74535200
C	-1.88833600	4.14571900	3.16097800
C	-3.07195800	3.50816800	2.92492100
C	0.59754100	4.19798500	2.93703900
C	1.74925100	3.58779400	2.49486100
C	1.68677700	2.32880000	1.84422200
C	2.94997200	1.70113800	1.38872300
C	3.79840900	2.40084200	0.50724000
C	4.96855400	1.81994300	0.03867300
C	5.34207000	0.52918400	0.46368400
C	4.53994200	-0.14631400	1.39697400
C	3.35131500	0.43853900	1.83935200
O	6.47356500	0.02855600	-0.07663800
C	6.88956300	-1.28293100	0.27616400
H	-2.52387000	-6.23989600	-3.44480800
H	-2.58871800	-4.46961300	-3.72750100
H	-4.04076900	-5.34635200	-3.11875900
H	-1.21980700	-4.47861200	0.36973800
H	-1.45614300	-2.32626200	1.59957500
H	-4.26279700	-3.18065900	-2.40838800
H	-4.53404000	-1.09422600	-1.13862000
H	-5.18372500	-0.18458000	1.06821900
H	-5.26223400	2.02395300	2.23355000
H	0.72951900	-0.60053200	2.65581000
H	-0.68665800	-0.86047000	4.66179100
H	-0.63779500	-5.14880500	4.11782600
H	0.77461300	-4.88783000	2.14451200
H	-2.64027200	-4.08348200	6.98766300
H	-1.18026000	-4.95424300	6.41663300
H	-2.62923700	-4.83185700	5.35779100
H	2.74797100	-4.46683900	1.29513000
H	4.20709700	-4.16334100	-0.69153600
H	5.00060300	-2.69127200	-2.55185900
H	4.89128200	-0.50985500	-3.74626800
H	3.88275700	1.76979000	-3.96198400
H	2.17633000	3.43405200	-3.19584000
H	-1.36189000	1.70415900	-1.72531900
H	-3.13157400	3.23889400	-1.00746400
H	-0.13720300	6.20004900	-0.02995500
H	1.64637300	4.64439800	-0.81121700
H	-4.53765800	6.28621300	0.43849400
H	-4.25485200	4.52192800	0.57181000
H	-4.37711300	5.29238100	-1.04671200
H	-1.86126200	5.11841100	3.65655900
H	-4.02138800	3.95640200	3.22549700
H	0.63233300	5.17023700	3.43418100
H	2.72492400	4.05189900	2.64251000
H	3.50835300	3.39224800	0.15269700
H	5.60554100	2.33734000	-0.68157600
H	4.81182500	-1.13627400	1.76138100
H	2.73323900	-0.10291200	2.55382800
H	7.79349700	-1.49035500	-0.31249200
H	6.11997200	-2.03358500	0.03022800
H	7.13544700	-1.35594700	1.35009500
C	-3.09809400	0.18910800	-3.42158800
F	-2.77641200	1.01220000	-4.39873300
F	-3.98704000	-0.70078900	-3.81951200
F	-3.54363000	0.86019100	-2.36810000
S	-0.90917700	-0.99182700	-2.81903600
O	-0.94246300	-0.63506100	-1.34568800
O	-0.03738700	-0.10518300	-3.61904100
Cl	0.29241500	-3.09859500	-2.81448000

·CF3
E (SMD/B3LYP-D3/def2-SVP) = -337.299090 au
H (SMD/B3LYP-D3/def2-SVP) = -337.282623 au

G (SMD/B3LYP-D3/def2-SVP) = -337.313703 au
E (SMD/B3LYP-D3/def2-TZVP//SMD/B3LYP-D3/def2-SVP) = -337.714254 au

C	-1.29477000	-2.16458700	-4.99206700
F	-0.92302400	-3.11758100	-5.82447900
F	-1.15025000	-0.96930400	-5.53161600
F	-2.52657900	-2.35354900	-4.56063200

A

E (SMD/B3LYP-D3/def2-SVP) = -5172.721758 au
H (SMD/B3LYP-D3/def2-SVP) = -5171.853414 au
G (SMD/B3LYP-D3/def2-SVP) = -5171.999640 au
E (SMD/B3LYP-D3/def2-TZVP//SMD/B3LYP-D3/def2-SVP) = -5176.187521 au

C	-2.93286400	-5.35613200	-3.05543700
O	-2.51283400	-5.18041800	-1.71167000
C	-2.72964000	-3.99338000	-1.10739500
C	-2.01997600	-3.76349900	0.08473700
C	-2.13362000	-2.54804500	0.74005500
C	-3.61125900	-3.00754300	-1.58684100
C	-3.74466200	-1.80158000	-0.89509400
C	-2.98035400	-1.54019500	0.25031100
C	-3.00437300	-0.20943000	0.88824600
C	-4.25165900	0.38125200	1.22490800
C	-4.29152200	1.59054600	1.87926500
C	-3.08145000	2.24882000	2.20270600
C	-1.87308400	1.62376600	1.80027900
N	-1.85145200	0.42412100	1.15449800
Cu	0.05279900	0.19485400	0.22730700
N	1.42169100	0.88344800	-1.16827100
C	2.37781400	-0.01121100	-1.51304000
C	2.47376600	-1.25600100	-0.77828400
N	1.63613700	-1.46407400	0.26365200
C	1.75635600	-2.56444500	1.01040100
C	0.89867300	-2.72514200	2.20725200
C	0.45487900	-1.60884800	2.94682600
C	-0.36623000	-1.75411900	4.05589100
C	-0.78471100	-3.03420800	4.46878900
C	-0.34507700	-4.16110600	3.75071100
C	0.49056100	-3.99774300	2.64401000
O	-1.59340800	-3.07994900	5.54655900
C	-2.06391300	-4.33637700	6.01588600
C	2.72391600	-3.55758000	0.68846800
C	3.56641500	-3.37277100	-0.38397900
C	3.47843900	-2.18669200	-1.14875000
C	4.37518700	-1.89667200	-2.22712800
C	4.29957500	-0.71011400	-2.89901900
C	3.30784400	0.26349500	-2.54965800
C	3.22227700	1.52550900	-3.18168700
C	2.25587600	2.42644000	-2.78897800
C	1.33006200	2.06621400	-1.78005600
C	0.25138100	3.00475100	-1.38044000
C	-1.09803400	2.63008600	-1.44007700
C	-2.11325200	3.49015800	-1.01690600
C	-1.78296900	4.75775200	-0.51040800
C	-0.43208300	5.15634400	-0.47816200
C	0.56757100	4.29602900	-0.91671200
O	-2.67983000	5.64837300	-0.03425500
C	-4.06343000	5.33047800	-0.07610200
N	0.52998900	1.72129500	1.60137300
C	-0.62137500	2.30075900	2.04328500
C	-0.62906800	3.56062200	2.69746400
C	-1.86792800	4.15101500	3.10876000
C	-3.05228100	3.51714200	2.86877900
C	0.61687800	4.20067300	2.88951300
C	1.76911500	3.58797800	2.45282500
C	1.70778300	2.32412600	1.81146800
C	2.97532700	1.69282700	1.37315700
C	3.82781300	2.38078600	0.48656100
C	5.00870600	1.80044000	0.04492500
C	5.38852300	0.52307600	0.50325500
C	4.58035200	-0.13991700	1.44033900
C	3.38109400	0.44339600	1.85538900
O	6.53181100	0.02162100	-0.01105300
C	6.95553600	-1.27736300	0.37665400
H	-2.55450700	-6.33735500	-3.37438000

H	-2.50598600	-4.57601800	-3.70910500
H	-4.03291800	-5.35292300	-3.15125200
H	-1.34694100	-4.54006900	0.45164700
H	-1.55087500	-2.35865000	1.63715100
H	-4.17974400	-3.16436800	-2.50358000
H	-4.40755900	-1.02990800	-1.29481900
H	-5.16825700	-0.15777200	0.98248700
H	-5.24393100	2.04814400	2.15737000
H	0.74683900	-0.60730900	2.63985600
H	-0.70633800	-0.88252600	4.61927900
H	-0.65452000	-5.16588800	4.03750200
H	0.79391500	-4.88897000	2.09099300
H	-2.70056000	-4.12314400	6.88521900
H	-1.23214300	-4.99036100	6.33067200
H	-2.66359200	-4.85777500	5.24954800
H	2.81796700	-4.44476200	1.31468500
H	4.32559700	-4.11813800	-0.63314200
H	5.13480000	-2.63852100	-2.48405900
H	4.99637500	-0.47492200	-3.70673000
H	3.92931100	1.77527800	-3.97656100
H	2.16775800	3.40499700	-3.26255600
H	-1.36285900	1.63853900	-1.80726700
H	-3.14823400	3.15403200	-1.06871500
H	-0.18979900	6.14516900	-0.08313300
H	1.61224000	4.61183600	-0.86653000
H	-4.59076200	6.18004700	0.37852000
H	-4.29230400	4.41766200	0.49865800
H	-4.41652900	5.19972100	-1.11412500
H	-1.83960900	5.12428300	3.60316700
H	-4.00173000	3.96867400	3.16421900
H	0.65053400	5.17526800	3.38213400
H	2.74470500	4.05242000	2.60002200
H	3.53383900	3.36216300	0.10795300
H	5.65005600	2.30777600	-0.67860100
H	4.85620500	-1.11954600	1.82878000
H	2.75779500	-0.08843900	2.57296200
H	7.87024100	-1.48833900	-0.19381900
H	6.19727100	-2.04114900	0.13600500
H	7.18612400	-1.32429000	1.45544900
S	-0.79424900	-1.18430300	-2.79033400
O	-0.85498500	-0.73865400	-1.33849800
O	0.08102400	-0.32456500	-3.61655900
Cl	0.42069300	-3.25246100	-2.63220300

[Cu]*
E (SMD/B3LYP-D3/def2-TZVP) = -4164.436951 au
H (SMD/B3LYP-D3/def2-TZVP) = -4163.587327 au
G (SMD/B3LYP-D3/def2-TZVP) = -4163.721607 au
E (SMD/B3LYP-D3/def2-TZVP//SMD/B3LYP-D3/def2-TZVP) = -4167.153874 au

C	-1.00949100	-4.84641400	-3.70514400
O	-1.65913100	-5.07378400	-2.46113300
C	-1.87182500	-4.03681400	-1.62690900
C	-2.48011300	-4.34312400	-0.39254200
C	-2.71106300	-3.34532300	0.54468000
C	-1.53546600	-2.70224700	-1.91454000
C	-1.76423900	-1.71361900	-0.95873300
C	-2.32868000	-2.01085900	0.29258600
C	-2.51611700	-0.96073900	1.31524800
C	-3.70341200	-0.92025600	2.09686100
C	-3.92208500	0.12034900	2.97228800
C	-2.95855400	1.15156000	3.09590200
C	-1.77048600	1.01328400	2.33569700
N	-1.56888800	-0.02761300	1.48522700
Cu	0.32149900	0.10609000	0.61163900
N	1.15370700	0.48744700	-1.17524900
C	1.81723000	-0.64216400	-1.62700100
C	1.75867400	-1.79944300	-0.82894000
N	1.06088500	-1.72645000	0.36728900
C	1.00383200	-2.81360000	1.18119400
C	0.33673800	-2.67228200	2.49777500
C	0.53891700	-1.53143200	3.30210500
C	-0.13124900	-1.36657900	4.50688400
C	-1.03722300	-2.34645400	4.95490400
C	-1.22030400	-3.51134600	4.18800500

C	-0.53288900	-3.66349100	2.98106700
O	-1.67561000	-2.08385000	6.11648400
C	-2.62525200	-3.01459900	6.61390300
C	1.57556900	-4.03019400	0.79197600
C	2.26910100	-4.13551800	-0.43106400
C	2.39156700	-3.01050900	-1.25289100
C	3.11514700	-2.99195800	-2.49976800
C	3.19541900	-1.85930900	-3.26174200
C	2.54896100	-0.63686000	-2.85767200
C	2.58762900	0.55392700	-3.58983600
C	1.88161400	1.67998800	-3.12505800
C	1.15313700	1.61375200	-1.92942700
C	0.35416300	2.76799300	-1.45315800
C	-0.93472600	2.58926300	-0.92602400
C	-1.65451300	3.64404600	-0.36409400
C	-1.08108300	4.92552800	-0.31574000
C	0.18206700	5.13556900	-0.90405900
C	0.88165600	4.07516300	-1.46771100
O	-1.65693900	5.99797900	0.27019300
C	-2.89681300	5.83481100	0.94428600
N	0.41997100	1.83252200	1.75993700
C	-0.73599700	2.01474200	2.45174800
C	-0.96181700	3.15381000	3.26332800
C	-2.18648500	3.27704000	3.99848300
C	-3.14422300	2.30336600	3.92904000
C	0.06226800	4.13125400	3.29519900
C	1.23099000	3.92169400	2.59776300
C	1.41418000	2.72820200	1.84677800
C	2.69993600	2.45693700	1.17118800
C	3.41527400	3.49143200	0.53083200
C	4.58687400	3.22496000	-0.16448200
C	5.09687900	1.91228600	-0.22977600
C	4.42648900	0.88076200	0.45129900
C	3.24658400	1.16329700	1.13712100
O	6.22013600	1.74272100	-0.95441000
C	6.75963700	0.43607500	-1.10703400
H	-0.92462700	-5.82662000	-4.19352700
H	0.00080800	-4.42488000	-3.56408100
H	-1.59529000	-4.17134300	-4.35295400
H	-2.74438000	-5.38219600	-0.18499300
H	-3.15366500	-3.61215300	1.50583200
H	-1.08725600	-2.42560600	-2.86822000
H	-1.49542300	-0.68465500	-1.20050100
H	-4.45535600	-1.69797600	1.96109300
H	-4.84430100	0.17379000	3.55589400
H	1.23118700	-0.75506700	2.96987700
H	0.02121000	-0.47448700	5.11810200
H	-1.90803900	-4.29343100	4.50961100
H	-0.71434000	-4.55784700	2.38258100
H	-3.02044100	-2.58756100	7.54578100
H	-2.16243400	-3.99235600	6.83524600
H	-3.45882200	-3.16335900	5.90496900
H	1.51514500	-4.88344000	1.46847500
H	2.73153800	-5.08007200	-0.72760300
H	3.60705200	-3.91318600	-2.82378600
H	3.75298000	-1.86247900	-4.20228500
H	3.14519000	0.59696800	-4.52875000
H	1.86061900	2.60187100	-3.70734000
H	-1.38442500	1.59448800	-0.93240500
H	-2.64266500	3.45165100	0.05256500
H	0.61017900	6.14004500	-0.87498100
H	1.87735700	4.25028700	-1.87861300
H	-3.14593300	6.81317200	1.37735300
H	-2.82260000	5.08988300	1.75566200
H	-3.70212000	5.53417500	0.25132900
H	-2.33180500	4.16365700	4.61970000
H	-4.07329800	2.38836300	4.49728600
H	-0.07800500	5.03727000	3.88949200
H	2.04168600	4.64888600	2.64527700
H	3.02448700	4.50999300	0.53921100
H	5.12216000	4.01943800	-0.68871000
H	4.80602500	-0.14038900	0.44682500
H	2.73954600	0.34885000	1.65731500
H	7.63965900	0.53843600	-1.75620300
H	6.03587300	-0.24749700	-1.58388200

H	7.07481300	0.01053000	-0.13848300

CF_3SO_2Cl

E (SMD/B3LYP-D3/def2-TZVP) = -1346.133681 au
H (SMD/B3LYP-D3/def2-TZVP) = -1346.099232 au
G (SMD/B3LYP-D3/def2-TZVP) = -1346.141756 au
E (SMD/B3LYP-D3/def2-TZVP//SMD/B3LYP-D3/def2-TZVP) = -1346.718981 au

S	-0.13797300	-2.19325500	-3.50218200
O	1.19540200	-2.14783300	-3.99213900
O	-0.63962100	-1.26528200	-2.54978900
C	-1.29492400	-2.16629900	-4.98999000
F	-0.92895800	-3.10400200	-5.84319300
F	-1.17354900	-0.96820800	-5.53784200
F	-2.53456800	-2.36446600	-4.58332200
Cl	-0.51700500	-4.09725300	-2.81814400

G

E (SMD/B3LYP-D3/def2-TZVP) = -5510.587697 au
H (SMD/B3LYP-D3/def2-TZVP) = -5509.701239 au
G (SMD/B3LYP-D3/def2-TZVP) = -5509.862346 au
E (SMD/B3LYP-D3/def2-TZVP//SMD/B3LYP-D3/def2-TZVP) = -5513.883905 au

C	-1.27936500	-5.11574100	-3.51288800
O	-1.90240400	-5.29348900	-2.24704400
C	-2.07414600	-4.22603100	-1.44261300
C	-2.66316300	-4.47496700	-0.18616000
C	-2.85774600	-3.43810700	0.71610800
C	-1.71149000	-2.91154000	-1.78266700
C	-1.90650700	-1.88347200	-0.86252000
C	-2.45951000	-2.11982900	0.40563500
C	-2.62888400	-1.02401400	1.38154700
C	-3.79217800	-0.97310900	2.19914200
C	-4.00397400	0.09429300	3.04182600
C	-3.05479100	1.14380100	3.09897900
C	-1.89168100	1.00111500	2.30153000
N	-1.69669000	-0.06443200	1.47787700
Cu	0.17018900	0.10611700	0.52823900
N	0.95661300	0.50782900	-1.28336800
C	1.64330900	-0.60182400	-1.74198900
C	1.63909800	-1.75430800	-0.93435500
N	0.97273000	-1.69187700	0.28147200
C	0.96291700	-2.78141500	1.10078800
C	0.32910900	-2.65115000	2.43407600
C	0.50342100	-1.49096100	3.21701300
C	-0.12741300	-1.34179400	4.44495200
C	-0.96542800	-2.35819000	4.94034000
C	-1.12431500	-3.53872600	4.19204300
C	-0.47819200	-3.67336300	2.96049500
O	-1.56549600	-2.11367600	6.12647000
C	-2.44099400	-3.08648400	6.67586400
C	1.55354500	-3.98223700	0.69897700
C	2.21551300	-4.07714400	-0.54445300
C	2.28715800	-2.95087700	-1.37276200
C	2.97169700	-2.92117700	-2.64165100
C	2.99881200	-1.79211500	-3.41355100
C	2.33192600	-0.58733500	-2.99699900
C	2.30771000	0.59646700	-3.74071100
C	1.58182200	1.70018600	-3.26694600
C	0.88974000	1.62051200	-2.04713700
C	0.03252200	2.74017900	-1.59342300
C	-1.23095500	2.49849000	-1.03301600
C	-2.04051100	3.53162200	-0.56077600
C	-1.58929200	4.85920700	-0.64614300
C	-0.34453500	5.12349900	-1.25245600
C	0.44612500	4.08177300	-1.72265500
O	-2.26953200	5.92918600	-0.18001400
C	-3.50339900	5.72539000	0.49369600
N	0.26062300	1.84584600	1.63211700
C	-0.87355700	2.02437200	2.35817000
C	-1.08694200	3.17888700	3.15194200
C	-2.28908300	3.30428500	3.92230500
C	-3.23292000	2.31501600	3.90632600
C	-0.07350800	4.16809300	3.13322000
C	1.07378400	3.96104400	2.39971400

C	1.24341800	2.75709400	1.66434100
C	2.49998800	2.48974800	0.93500600
C	3.13158100	3.50519000	0.18716300
C	4.26075100	3.23157600	-0.57324300
C	4.80870300	1.93294300	-0.59728400
C	4.22550500	0.92579800	0.19230600
C	3.08637800	1.21361500	0.94216700
O	5.88082300	1.75251600	-1.39402100
C	6.45143800	0.45577200	-1.51265400
H	-1.23170200	-6.11023800	-3.97663600
H	-0.25580800	-4.71620400	-3.40769200
H	-1.86452600	-4.44253100	-4.16341800
H	-2.94314800	-5.49995800	0.06606000
H	-3.28464100	-3.66309000	1.69494700
H	-1.26986900	-2.67871800	-2.74930600
H	-1.61769400	-0.87097900	-1.14263500
H	-4.53421900	-1.76739300	2.11723000
H	-4.90818600	0.15264000	3.65244300
H	1.14772500	-0.68777600	2.85439200
H	0.00657600	-0.43561400	5.03961300
H	-1.76291100	-4.34732700	4.54785600
H	-0.64387900	-4.58120600	2.37795900
H	-2.81328000	-2.67064500	7.62215200
H	-1.91824600	-4.03686300	6.88306800
H	-3.29837400	-3.28572000	6.00889200
H	1.53477100	-4.83434300	1.37956600
H	2.69140200	-5.01159500	-0.85110300
H	3.47610100	-3.83206800	-2.97566600
H	3.52535600	-1.78787600	-4.37181600
H	2.82826600	0.64580900	-4.70043700
H	1.50024400	2.60797700	-3.86453600
H	-1.59345300	1.47286000	-0.95237800
H	-3.00867200	3.29009900	-0.12316400
H	-0.00901600	6.16059600	-1.32262500
H	1.41961300	4.30729100	-2.16155500
H	-3.85330400	6.71808100	0.80830000
H	-3.37927200	5.08776600	1.38689200
H	-4.26090900	5.27173100	-0.16928300
H	-2.42723700	4.20329000	4.52723500
H	-4.14479000	2.39962400	4.50187500
H	-0.20505200	5.08211800	3.71727400
H	1.87744400	4.69786600	2.40316400
H	2.70503400	4.50940700	0.16412500
H	4.73004500	4.00699500	-1.18239500
H	4.63826000	-0.08205900	0.21972300
H	2.64146000	0.41844300	1.54306100
H	7.27893900	0.54632800	-2.22924800
H	5.71937900	-0.27553900	-1.89708000
H	6.84924600	0.09599500	-0.54788600
S	-2.16020200	0.24011000	-4.00634500
O	-0.93456300	-0.36737600	-3.62153100
O	-3.12360200	0.69310600	-3.06405800
C	-1.74838400	1.68760700	-5.14903600
F	-0.80865000	1.31045500	-5.99452800
F	-1.31419700	2.67046900	-4.37900100
F	-2.83699900	2.06015400	-5.79565200
Cl	-3.09459400	-1.00983400	-5.34917000

TS$_G$
E (SMD/B3LYP-D3/def2-TZVP) = -5510.587455 au
H (SMD/B3LYP-D3/def2-TZVP) = -5509.702568 au
G (SMD/B3LYP-D3/def2-TZVP) = -5509.864459 au
E (SMD/B3LYP-D3/def2-TZVP//SMD/B3LYP-D3/def2-TZVP) = -5513.883692 au

C	-1.28641600	-5.13403500	-3.48774800
O	-1.90597100	-5.30625400	-2.21934900
C	-2.08072700	-4.23440300	-1.42157100
C	-2.66812200	-4.47731500	-0.16317000
C	-2.86478700	-3.43543100	0.73265600
C	-1.72259100	-2.92101200	-1.77038400
C	-1.92088100	-1.88732400	-0.85719900
C	-2.47094100	-2.11785400	0.41337600
C	-2.64065800	-1.01780200	1.38428100
C	-3.80302200	-0.96475800	2.20296000
C	-4.01385800	0.10443400	3.04356400

C	-3.06427200	1.15370800	3.09788600
C	-1.90235500	1.00965800	2.29906300
N	-1.70854600	-0.05752900	1.47669700
Cu	0.14945500	0.11517700	0.51837900
N	0.93459400	0.51776500	-1.29133500
C	1.62933800	-0.58654200	-1.74765700
C	1.63178900	-1.74113600	-0.94097800
N	0.96800300	-1.68418500	0.27495400
C	0.96260000	-2.76985200	1.09135600
C	0.32760300	-2.64577000	2.42458300
C	0.49215200	-1.48454100	3.20770700
C	-0.13921400	-1.34234300	4.43597700
C	-0.96799300	-2.36690900	4.93047500
C	-1.11719400	-3.54811400	4.18103200
C	-0.46990200	-3.67615500	2.94969400
O	-1.56860600	-2.12963400	6.11705700
C	-2.43257500	-3.11221800	6.66810500
C	1.55941200	-3.97135900	0.68830600
C	2.21843800	-4.06071300	-0.55275200
C	2.28387300	-2.93465400	-1.38204500
C	2.96387600	-2.90427700	-2.65192900
C	2.98391400	-1.77413100	-3.42203500
C	2.31488300	-0.57089400	-3.00184400
C	2.29062500	0.61494300	-3.74460800
C	1.57065700	1.72025700	-3.26510500
C	0.87407100	1.63770300	-2.04967900
C	0.02008300	2.75743600	-1.59182600
C	-1.24198900	2.51815900	-1.02678800
C	-2.04322900	3.55192500	-0.54180000
C	-1.58536000	4.87761500	-0.61882300
C	-0.34380400	5.14040300	-1.23255500
C	0.43882200	4.09831200	-1.71484800
O	-2.25539700	5.94694100	-0.13634200
C	-3.48334600	5.74280800	0.54796600
N	0.24880000	1.85266900	1.62555700
C	-0.88386600	2.03236400	2.35348800
C	-1.09486400	3.18734800	3.14712500
C	-2.29610100	3.31442500	3.91850700
C	-3.24076300	2.32590100	3.90418900
C	-0.08008900	4.17516100	3.12703300
C	1.06633200	3.96617400	2.39267500
C	1.23347200	2.76195400	1.65716800
C	2.48916200	2.49149400	0.92767000
C	3.12477000	3.50515900	0.18078800
C	4.25232000	3.22747800	-0.58051400
C	4.79447800	1.92640600	-0.60662600
C	4.20806400	0.92114900	0.18298100
C	3.07083800	1.21324900	0.93396800
O	5.86434500	1.74176700	-1.40533900
C	6.42682800	0.44185000	-1.52855800
H	-1.23588000	-6.13125700	-3.94527300
H	-0.26420900	-4.72982300	-3.38739700
H	-1.87570900	-4.46726900	-4.14108900
H	-2.94491600	-5.50148500	0.09578800
H	-3.28957700	-3.65550100	1.71351900
H	-1.28208400	-2.69342100	-2.73858000
H	-1.63862500	-0.87433100	-1.14385200
H	-4.54513500	-1.75921900	2.12363000
H	-4.91741000	0.16414800	3.65501400
H	1.12957200	-0.67585900	2.84547300
H	-0.01269800	-0.43590500	5.03178600
H	-1.74852500	-4.36255700	4.53633400
H	-0.62733000	-4.58521200	2.36683600
H	-2.80670300	-2.70074600	7.61552100
H	-1.89892800	-4.05689800	6.87325700
H	-3.28934300	-3.31994200	6.00305100
H	1.54343500	-4.82427000	1.36763500
H	2.69744300	-4.99302700	-0.86138300
H	3.46984100	-3.81341700	-2.98800900
H	3.50699600	-1.76654700	-4.38210400
H	2.80936200	0.66518900	-4.70508900
H	1.49497500	2.63123300	-3.85856500
H	-1.60910800	1.49368400	-0.95191000
H	-3.00938400	3.31215200	-0.09882000
H	-0.00325800	6.17624900	-1.29678500

H	1.41115300	4.32239700	-2.15703900
H	-3.82413500	6.73394800	0.87714700
H	-3.35367100	5.09456500	1.43274100
H	-4.25035500	5.30088700	-0.11203300
H	-2.43284400	4.21395500	4.52296100
H	-4.15197100	2.41166900	4.50056400
H	-0.20983200	5.08960800	3.71080400
H	1.87123400	4.70164100	2.39547100
H	2.70247800	4.51116100	0.15904900
H	4.72442400	4.00145700	-1.18931500
H	4.61690800	-0.08831400	0.20960600
H	2.62310700	0.41984300	1.53495500
H	7.25269800	0.52904000	-2.24744100
H	5.68919400	-0.28413600	-1.91244800
H	6.82538700	0.07749200	-0.56582900
S	-2.07905700	0.17348000	-4.04310500
O	-0.85300600	-0.45496600	-3.68607100
O	-3.01310500	0.63655400	-3.07300700
C	-1.66916500	1.63499600	-5.17722700
F	-0.69493900	1.28147000	-5.99392000
F	-1.28680400	2.63743400	-4.40363300
F	-2.74782600	1.97490100	-5.85918200
Cl	-3.07560600	-1.08013200	-5.38976900

TS_D

E (SMD/B3LYP-D3/def2-SVP) = -1008.565987 au
H (SMD/B3LYP-D3/def2-SVP) = -1008.553349 au
G (SMD/B3LYP-D3/def2-SVP) = -1008.592695 au
E (SMD/B3LYP-D3/def2-TZVP//SMD/B3LYP-D3/def2-SVP) = -1009.124473 au

S	-0.05410100	-1.35140000	-3.87602800
O	1.37933700	-1.47742100	-4.15809800
O	-0.45099400	-0.51668400	-2.73761300
Cl	-0.89242800	-6.15088200	-2.10711100

SO_2

E (SMD/B3LYP-D3/def2-SVP) = -548.363425 au
H (SMD/B3LYP-D3/def2-SVP) = -548.352629 au
G (SMD/B3LYP-D3/def2-SVP) = -548.381545 au
E (SMD/B3LYP-D3/def2-TZVP//SMD/B3LYP-D3/def2-SVP) = -548.726952 au

S	-0.11854400	-2.31938500	-3.40155900
O	1.16455600	-2.09172800	-4.07213400
O	-0.68375400	-1.21736800	-2.61802300

Cl^-

E (SMD/B3LYP-D3/def2-SVP) = -460.202036 au
H (SMD/B3LYP-D3/def2-SVP) = -460.199675 au
G (SMD/B3LYP-D3/def2-SVP) = -460.217059 au
E (SMD/B3LYP-D3/def2-TZVP//SMD/B3LYP-D3/def2-SVP) = -460.396980 au

Cl	-0.84141100	-0.63376900	-1.96516600

S2

E (SMD/B3LYP-D3/def2-SVP) = -348.730812 au
H (SMD/B3LYP-D3/def2-SVP) = -348.560561 au
G (SMD/B3LYP-D3/def2-SVP) = -348.603778 au
E (SMD/B3LYP-D3/def2-TZVP//SMD/B3LYP-D3/def2-SVP) = -349.110770 au

C	7.41230600	5.04018000	-6.13686400
C	8.72270400	5.13851900	-5.88962500
C	9.34055100	5.28179500	-4.52170500
H	9.92187300	6.21894600	-4.48257800
H	8.54166000	5.36081900	-3.76495400
H	7.02846500	4.93129400	-7.15646500
H	6.67388700	5.06851700	-5.32692200
H	9.43402600	5.09920800	-6.72533400
C	10.25849800	4.12035600	-4.17841600
C	9.72480100	2.83503500	-3.97890000
C	11.64725100	4.29149700	-4.07271400
C	10.55493700	1.75287700	-3.67412000
H	8.64447100	2.68534600	-4.06703200
C	12.48311600	3.20930700	-3.76980000
H	12.07937200	5.28479000	-4.22645800

C	11.93980800	1.93655900	-3.56866800
H	10.12080800	0.76125200	-3.51814500
H	13.56290800	3.36352100	-3.69023500
H	12.59062700	1.09078700	-3.33072400

TS_{S2}
E (SMD/B3LYP-D3/def2-SVP) = -686.034961 au
H (SMD/B3LYP-D3/def2-SVP) = -685.847838 au
G (SMD/B3LYP-D3/def2-SVP) = -685.904472 au
E (SMD/B3LYP-D3/def2-TZVP//SMD/B3LYP-D3/def2-SVP) = -686.826734 au

C	7.59142600	4.69731400	-6.21987600
C	8.85842300	5.11017200	-5.98170400
C	9.44486200	5.28853100	-4.59814800
H	10.06888600	6.19482700	-4.56830900
H	8.62654200	5.42540400	-3.87231900
H	7.24306900	4.48901000	-7.23585500
H	6.85986400	4.61264700	-5.40898400
H	9.58162900	5.11223800	-6.80652100
C	10.29803500	4.09673800	-4.19638500
C	9.69927200	2.85853700	-3.90651900
C	11.69622200	4.19594100	-4.13102400
C	10.47619600	1.75193700	-3.55345300
H	8.61077500	2.76431900	-3.96329000
C	12.47872600	3.08882500	-3.78002000
H	12.17788300	5.15246400	-4.35475900
C	11.87126300	1.86327100	-3.48947600
H	9.99205400	0.79779300	-3.32702800
H	13.56695700	3.18647100	-3.73310300
H	12.48025300	0.99791700	-3.21415500
C	8.58008500	7.48521100	-6.45159000
F	7.80345700	7.61363300	-7.51811600
F	7.99232600	8.01992400	-5.38815200
F	9.75581200	8.06812600	-6.66499400

$TS_{S2\text{-}R^{\bullet}(S2)}$
E (SMD/B3LYP-D3/def2-SVP) = -686.036488 au
H (SMD/B3LYP-D3/def2-SVP) = -685.849157 au
G (SMD/B3LYP-D3/def2-SVP) = -685.907807 au
E (SMD/B3LYP-D3/def2-TZVP//SMD/B3LYP-D3/def2-SVP) = -686.828965 au

C	7.44994200	4.87751300	-6.26236000
C	8.77429400	4.90724000	-6.02143900
C	9.39425100	5.16573900	-4.67522500
H	9.96974700	6.10657300	-4.72514500
H	8.59773100	5.31288900	-3.92680700
H	7.05608700	4.67728600	-7.26307000
H	6.72249400	4.95538700	-5.44742600
H	9.47737000	4.77308000	-6.85325000
C	10.31748500	4.04251000	-4.23640300
C	9.78500800	2.79090700	-3.88083300
C	11.70979900	4.21289100	-4.19870100
C	10.62080800	1.74090300	-3.49071200
H	8.70140700	2.64207800	-3.91260100
C	12.55093900	3.16268300	-3.81056100
H	12.14011900	5.18046400	-4.47365400
C	12.00925000	1.92339700	-3.45478000
H	10.18812100	0.77559000	-3.21314500
H	13.63354100	3.31555600	-3.78561100
H	12.66436400	1.10273800	-3.15007700
C	6.99492900	7.40508900	-6.52144400
F	7.12958000	7.78997200	-7.78298500
F	5.78833900	7.71853200	-6.06706300
F	7.93117400	7.96489300	-5.76619200

$R^{\cdot}$ (S2)
E (SMD/B3LYP-D3/def2-SVP) = -686.098829 au
H (SMD/B3LYP-D3/def2-SVP) = -685.909870 au
G (SMD/B3LYP-D3/def2-SVP) = -685.964791 au
E (SMD/B3LYP-D3/def2-TZVP//SMD/B3LYP-D3/def2-SVP) = -686.884950 au

C	7.30809100	5.32096500	-6.35790000
C	8.63500000	4.77574500	-5.94260200
C	9.16787100	4.97759900	-4.55945600
H	9.60799100	5.99214200	-4.46902900

H	8.32586300	4.96366800	-3.84247400
H	6.94914900	4.84954200	-7.28595200
H	6.54461100	5.16802800	-5.57586600
H	9.31679000	4.41624500	-6.71961000
C	10.20581200	3.94943700	-4.15665000
C	9.83714600	2.60306600	-3.98709900
C	11.54974100	4.30370400	-3.96416800
C	10.78427400	1.63929800	-3.62998400
H	8.79307500	2.31176100	-4.13676000
C	12.50236200	3.34109000	-3.60723800
H	11.85363500	5.34669600	-4.09397500
C	12.12278800	2.00573700	-3.43874800
H	10.47776300	0.59765100	-3.49906900
H	13.54485600	3.63741000	-3.46084300
H	12.86487800	1.25297600	-3.15918200
C	7.32474700	6.81422900	-6.62732900
F	6.11843900	7.26361500	-7.02764700
F	7.66048400	7.52463100	-5.52727500
F	8.20874500	7.14882400	-7.58937800

TS_{B-A}

E (SMD/B3LYP-D3/def2-SVP) = -5172.721749 au
H (SMD/B3LYP-D3/def2-SVP) = -5171.853456 au
G (SMD/B3LYP-D3/def2-SVP) = -5172.000053 au
E (SMD/B3LYP-D3/def2-TZVP//SMD/B3LYP-D3/def2-SVP) = -5176.187513 au

C	-3.03661100	-5.26836600	-3.03003900
O	-2.59729200	-5.11081600	-1.69027300
C	-2.79809600	-3.92943100	-1.06957800
C	-2.07068800	-3.71778600	0.11521000
C	-2.16714000	-2.50910900	0.78569000
C	-3.67971600	-2.93258700	-1.52566900
C	-3.79536700	-1.73362700	-0.81865600
C	-3.01355200	-1.49023800	0.31881700
C	-3.02081600	-0.16637400	0.97162000
C	-4.26033300	0.42478600	1.33527500
C	-4.28533400	1.62701300	2.00322900
C	-3.06772400	2.27813900	2.31241100
C	-1.86848600	1.65363800	1.88266300
N	-1.86117600	0.46017300	1.22506300
Cu	0.03090100	0.22863500	0.26959100
N	1.38122600	0.91931600	-1.14081900
C	2.32735300	0.02173200	-1.50484900
C	2.42686400	-1.22830800	-0.77943300
N	1.60272700	-1.43896200	0.27276100
C	1.72636000	-2.54515500	1.01007300
C	0.88258900	-2.71036200	2.21629700
C	0.45381400	-1.59814700	2.97107500
C	-0.35418200	-1.74918800	4.08899600
C	-0.77432700	-3.03073500	4.49579700
C	-0.35031600	-4.15341500	3.76196800
C	0.47247800	-3.98454000	2.64653900
O	-1.56933600	-3.08204200	5.58346000
C	-2.03972400	-4.34044200	6.04767500
C	2.68408100	-3.54119800	0.66815200
C	3.51291600	-3.35350400	-0.41441900
C	3.42094000	-2.16196500	-1.17014400
C	4.30385200	-1.86978200	-2.25931500
C	4.22511400	-0.67858300	-2.92261100
C	3.24356600	0.29794600	-2.55324300
C	3.15457100	1.56382500	-3.17702800
C	2.19812300	2.46697500	-2.76545800
C	1.28567900	2.10595100	-1.74461700
C	0.21917300	3.04892900	-1.32214300
C	-1.13384700	2.68344200	-1.35676400
C	-2.13484200	3.54827100	-0.90988000
C	-1.78628400	4.81108500	-0.40364200
C	-0.43252500	5.20092700	-0.39676600
C	0.55245900	4.33625900	-0.85908800
O	-2.66739700	5.70480900	0.09544700
C	-4.05362300	5.39618000	0.08026300
N	0.53040500	1.74665000	1.64283800
C	-0.61101200	2.32509000	2.11028200
C	-0.60315200	3.57817800	2.77736800
C	-1.83279800	4.16767900	3.21660300

C	-3.02293000	3.53934600	2.99109500
C	0.64798900	4.21247200	2.95405300
C	1.79055200	3.60015700	2.49218600
C	1.71395100	2.34253900	1.84052600
C	2.97219600	1.70840000	1.38036100
C	3.81763600	2.39891700	0.48908800
C	4.98945300	1.81540600	0.02785800
C	5.36745200	0.53206500	0.47084500
C	4.56736100	-0.13384700	1.41279100
C	3.37672200	0.45282800	1.84745400
O	6.50116300	0.02827000	-0.06207100
C	6.92185100	-1.27664500	0.30871900
H	-2.66848600	-6.24796100	-3.36553500
H	-2.61418700	-4.48319700	-3.68054700
H	-4.13785100	-5.25768000	-3.11074800
H	-1.39785800	-4.50285800	0.46402400
H	-1.57081800	-2.33379400	1.67666700
H	-4.26192800	-3.07538900	-2.43609400
H	-4.45855100	-0.95312700	-1.20035000
H	-5.18253600	-0.10880700	1.10231700
H	-5.23135100	2.08445500	2.30258600
H	0.74703800	-0.59537100	2.66898700
H	-0.68255900	-0.88096500	4.66441700
H	-0.66201200	-5.15906000	4.04321200
H	0.76378200	-4.87237100	2.08170500
H	-2.66302300	-4.13218300	6.92781800
H	-1.20701900	-5.00155300	6.34458500
H	-2.65280900	-4.85156900	5.28501400
H	2.78182300	-4.43334100	1.28668000
H	4.26431100	-4.10140200	-0.67913200
H	5.05543700	-2.61419500	-2.53196900
H	4.91146300	-0.44212100	-3.73885400
H	3.85101900	1.81480100	-3.98081900
H	2.10784500	3.44844900	-3.23256100
H	-1.41259600	1.69516900	-1.72274400
H	-3.17288300	3.21931000	-0.94267400
H	-0.17588100	6.18624300	-0.00203200
H	1.59990500	4.64530000	-0.82819800
H	-4.56594400	6.24727900	0.54898400
H	-4.27700600	4.48248100	0.65576300
H	-4.42841500	5.27223400	-0.95094700
H	-1.79299000	5.13547200	3.72085700
H	-3.96562900	3.99014900	3.30846600
H	0.69337300	5.18187200	3.45591500
H	2.77023900	4.05935000	2.62819600
H	3.52490300	3.38533600	0.12282100
H	5.62477700	2.32503800	-0.69936300
H	4.84249900	-1.11815600	1.78976600
H	2.75908100	-0.08127300	2.56825100
H	7.82712400	-1.48854400	-0.27628600
H	6.15543500	-2.03354100	0.07235100
H	7.16707300	-1.33427400	1.38375900
S	-0.86405000	-1.12826700	-2.74598700
O	-0.90081600	-0.69456100	-1.28979500
O	0.00351800	-0.26654500	-3.57825400
Cl	0.34180100	-3.20508800	-2.62171800

TS_{B-C}
E (SMD/B3LYP-D3/def2-SVP) = -4624.315742 au
H (SMD/B3LYP-D3/def2-SVP) = -4623.461013 au
G (SMD/B3LYP-D3/def2-SVP) = -4623.597046 au
E (SMD/B3LYP-D3/def2-TZVP//SMD/B3LYP-D3/def2-SVP) = -4627.435900 au

C	-3.73663400	-5.37970200	-2.79841900
O	-2.73664300	-5.00201900	-1.86335400
C	-2.84447700	-3.82418400	-1.21348900
C	-1.80787200	-3.51227000	-0.31452300
C	-1.82505200	-2.31379300	0.37776100
C	-3.90729700	-2.91729500	-1.37857000
C	-3.91844300	-1.72391000	-0.65651300
C	-2.87718600	-1.39395800	0.22798800
C	-2.89650200	-0.13501000	0.99493700
C	-4.14131500	0.38340000	1.45400300
C	-4.18719500	1.54779700	2.18181000
C	-2.98724000	2.23892900	2.47382000

C	-1.78058400	1.66940700	1.99525900
N	-1.75233600	0.51248500	1.27306900
Cu	0.15636800	0.28111400	0.41358100
N	1.37647200	0.89994200	-1.07224300
C	2.32327800	0.00458500	-1.44010500
C	2.41572800	-1.24530800	-0.71797200
N	1.58659100	-1.44793700	0.33513000
C	1.72555700	-2.53846000	1.09710900
C	0.86849100	-2.69772800	2.29452600
C	0.45531400	-1.58431100	3.05676000
C	-0.40520700	-1.72715300	4.13495000
C	-0.88996100	-2.99999400	4.49541400
C	-0.47344300	-4.12463900	3.75997800
C	0.39841200	-3.96511500	2.68144100
O	-1.73579500	-3.04071900	5.54374700
C	-2.27976200	-4.28706800	5.95765000
C	2.69366200	-3.53012000	0.77229800
C	3.51766500	-3.35701000	-0.31715000
C	3.41461500	-2.17870300	-1.09477900
C	4.30559600	-1.88457100	-2.17916600
C	4.23089200	-0.69249500	-2.84154200
C	3.24532900	0.28445100	-2.48034200
C	3.15383500	1.55165200	-3.10329500
C	2.18827500	2.44787600	-2.69655800
C	1.27457600	2.08759800	-1.67471800
C	0.20684600	3.02814900	-1.25146000
C	-1.14285300	2.64391200	-1.24441400
C	-2.13886000	3.51316700	-0.79459100
C	-1.79054900	4.78921800	-0.32178300
C	-0.44121800	5.19358900	-0.35657100
C	0.54025700	4.32867800	-0.82562700
O	-2.66789100	5.68410700	0.18295900
C	-4.04748100	5.35099200	0.23239700
N	0.61050500	1.83778500	1.75404500
C	-0.54200700	2.37599700	2.23055000
C	-0.56278900	3.62245800	2.91023600
C	-1.80244600	4.15933600	3.38603600
C	-2.97204000	3.48533200	3.18085100
C	0.67103400	4.29857800	3.06106000
C	1.82637600	3.73146800	2.57026600
C	1.77654600	2.47017300	1.92388600
C	3.02681800	1.84516000	1.43472600
C	3.87523100	2.54473600	0.55419100
C	5.03003200	1.95076600	0.06223900
C	5.38577200	0.64658700	0.46057300
C	4.57561500	-0.03696600	1.38160100
C	3.40280100	0.56079900	1.84660300
O	6.50882000	0.14030800	-0.09209200
C	6.93229600	-1.16630500	0.26964100
H	-3.43123500	-6.35472100	-3.20206200
H	-3.81273400	-4.65524700	-3.62795600
H	-4.72556000	-5.48372300	-2.31854300
H	-0.99149400	-4.22555800	-0.18171100
H	-1.02200400	-2.08819700	1.07156900
H	-4.72166800	-3.12725300	-2.07179400
H	-4.74450400	-1.02775000	-0.81847300
H	-5.05676000	-0.16840700	1.24231000
H	-5.13927200	1.94756700	2.53877800
H	0.80353800	-0.58748700	2.79011200
H	-0.72399400	-0.85942400	4.71651600
H	-0.83165300	-5.12310500	4.00962000
H	0.67937300	-4.85035300	2.10693100
H	-2.93804500	-4.06930300	6.80954500
H	-1.49057800	-4.98723900	6.28223100
H	-2.87341900	-4.75768600	5.15477900
H	2.79976900	-4.40776700	1.41029800
H	4.27667900	-4.10199200	-0.56773300
H	5.06252800	-2.62627300	-2.44373600
H	4.92480400	-0.45490300	-3.65095900
H	3.85342000	1.80772200	-3.90261400
H	2.09426400	3.42795600	-3.16577200
H	-1.40580600	1.63841500	-1.58518100
H	-3.17517400	3.17641000	-0.79476300
H	-0.18533400	6.18950400	0.01121600
H	1.58488100	4.64916700	-0.82783300

H	-4.55598000	6.20396400	0.70196700
H	-4.22896400	4.44742800	0.83801500
H	-4.46282000	5.19568400	-0.77886400
H	-1.78861800	5.11982900	3.90561200
H	-3.92312400	3.89103400	3.53242300
H	0.69322900	5.26630900	3.56785200
H	2.79126200	4.22673400	2.68519400
H	3.60108500	3.54912400	0.22364400
H	5.67063300	2.47058900	-0.65311900
H	4.82982700	-1.04107100	1.71896200
H	2.77398900	0.01410500	2.54846800
H	7.83809300	-1.37216200	-0.31665500
H	6.16867500	-1.92424500	0.02855600
H	7.17697300	-1.22973200	1.34449700
Cl	-0.89486300	-0.70135300	-2.54906800

C
E (SMD/B3LYP-D3/def2-SVP) = -4624.335397 au
H (SMD/B3LYP-D3/def2-SVP) = -4623.480090 au
G (SMD/B3LYP-D3/def2-SVP) = -4623.618073 au
E (SMD/B3LYP-D3/def2-TZVP//SMD/B3LYP-D3/def2-SVP) = -4627.446090 au

C	-3.98378300	-5.85180000	-2.29548200
O	-3.02056400	-5.42204800	-1.34448100
C	-3.08017400	-4.16198700	-0.86319200
C	-2.08981300	-3.80398000	0.07137400
C	-2.06393100	-2.52593600	0.60704200
C	-4.05120300	-3.21323400	-1.23199400
C	-4.02274000	-1.93738300	-0.66585900
C	-3.02664300	-1.56584100	0.25170300
C	-3.00654100	-0.21666500	0.85051300
C	-4.24704300	0.37312500	1.22241300
C	-4.27673500	1.58877400	1.86062900
C	-3.06205600	2.24819400	2.15773000
C	-1.85859200	1.61771100	1.74395200
N	-1.84798100	0.42258000	1.08632500
Cu	0.05920800	0.21146700	0.12495300
N	1.49862300	1.04510100	-1.16200100
C	2.49395200	0.19049200	-1.49762400
C	2.60141800	-1.07631000	-0.79700200
N	1.73920500	-1.34285800	0.21330100
C	1.87356900	-2.46047500	0.93232100
C	0.98475700	-2.68512000	2.09607200
C	0.47345000	-1.60572000	2.84745000
C	-0.38790200	-1.81105400	3.91531000
C	-0.78057800	-3.11578800	4.27239000
C	-0.27186300	-4.20618500	3.54403800
C	0.60322200	-3.98320300	2.47925800
O	-1.63627800	-3.21990200	5.30880600
C	-2.09225000	-4.50391100	5.71287400
C	2.88280800	-3.41312100	0.61383500
C	3.75310500	-3.16904500	-0.42362900
C	3.65055900	-1.96305200	-1.15733200
C	4.58434900	-1.60811300	-2.18485600
C	4.49954900	-0.40137300	-2.81789600
C	3.46240300	0.52832600	-2.47977400
C	3.37324900	1.81381400	-3.06419000
C	2.37140600	2.67615900	-2.67462300
C	1.41084900	2.25474900	-1.71925000
C	0.31461100	3.17224800	-1.31422800
C	-1.03377300	2.78932100	-1.37679600
C	-2.05251000	3.64769700	-0.95767800
C	-1.73109800	4.91670400	-0.44889000
C	-0.38259300	5.32098700	-0.40939400
C	0.62170000	4.46504800	-0.84589700
O	-2.63435800	5.80408400	0.02277800
C	-4.01694400	5.48616700	-0.03714000
N	0.55486800	1.68878200	1.60279900
C	-0.60151600	2.28250800	2.01158500
C	-0.61221300	3.54179200	2.67154200
C	-1.84848300	4.14446100	3.06923000
C	-3.03298900	3.51519200	2.82429500
C	0.63252800	4.17076100	2.89794600
C	1.78924800	3.54487900	2.49639500
C	1.73053100	2.28096700	1.85491500

C	3.01563000	1.64492100	1.47159500
C	3.90593700	2.33129900	0.62176700
C	5.11125200	1.75716000	0.24177600
C	5.47820000	0.48682800	0.72781800
C	4.63321300	-0.17229500	1.63478100
C	3.41182200	0.40624900	1.98829900
O	6.64667000	-0.01147600	0.26868100
C	7.05988200	-1.30564400	0.68175800
H	-3.73686900	-6.89446100	-2.53789500
H	-3.94151000	-5.24863800	-3.21928500
H	-5.00823900	-5.81134000	-1.88559500
H	-1.34249400	-4.54687300	0.35703300
H	-1.28650700	-2.25414700	1.31379800
H	-4.82606700	-3.45623400	-1.95888800
H	-4.77490300	-1.21136900	-0.98318800
H	-5.17158500	-0.16955700	1.02596300
H	-5.22426000	2.04577200	2.15587300
H	0.73781900	-0.58693300	2.57522300
H	-0.78378800	-0.96826400	4.48601900
H	-0.56034000	-5.22837500	3.78816100
H	0.95872000	-4.84747400	1.91455000
H	-2.77878700	-4.33790900	6.55408900
H	-1.25864600	-5.14449900	6.04959200
H	-2.63586500	-5.01611200	4.90001800
H	2.98608500	-4.31526200	1.21675300
H	4.54609300	-3.88019200	-0.66717500
H	5.38012100	-2.31549200	-2.42935900
H	5.22532400	-0.11452600	-3.58229100
H	4.10710400	2.11252100	-3.81673500
H	2.28422000	3.67255700	-3.10966500
H	-1.29232400	1.79541200	-1.74730000
H	-3.08625700	3.30802900	-1.01346400
H	-0.14571400	6.31014100	-0.01172900
H	1.66418400	4.78796300	-0.79127900
H	-4.55042300	6.33654800	0.40897400
H	-4.25391200	4.57450200	0.53616000
H	-4.35652700	5.35364000	-1.07948100
H	-1.81671900	5.11739300	3.56405600
H	-3.98273200	3.96662200	3.11913300
H	0.66123800	5.14432200	3.39293700
H	2.76532300	3.99722300	2.67482900
H	3.62449500	3.30725500	0.22035200
H	5.78175000	2.26337100	-0.45559100
H	4.89866600	-1.14478800	2.04773900
H	2.76275000	-0.12215400	2.68498900
H	8.00056000	-1.51410000	0.15417000
H	6.31778200	-2.07540200	0.41125100
H	7.24222100	-1.34686500	1.77005000
Cl	-0.77246800	-0.74206600	-1.82647500

MECP1
E (SMD/B3LYP-D3/def2-SVP) = -4164.1942440000

C	-1.00711200	-4.86290800	-3.71786100
O	-1.65745400	-5.08968900	-2.47387100
C	-1.86852000	-4.05236000	-1.63953400
C	-2.48833400	-4.35526100	-0.40898200
C	-2.71822000	-3.35679900	0.52735100
C	-1.51929000	-2.72088900	-1.92227700
C	-1.74245500	-1.73223400	-0.96537700
C	-2.32155000	-2.02407500	0.28089000
C	-2.49832200	-0.97839700	1.30634600
C	-3.67132300	-0.94531800	2.10652700
C	-3.89573100	0.09778100	2.98001800
C	-2.93874200	1.13647400	3.09127300
C	-1.75631000	1.00557000	2.32248200
N	-1.55322500	-0.03091200	1.45913200
Cu	0.34266900	0.09500000	0.62595100
N	1.14501400	0.48917000	-1.18672200
C	1.79534400	-0.62341300	-1.63850600
C	1.71004400	-1.80229500	-0.82487300
N	0.99032300	-1.73555500	0.32215800
C	0.96644100	-2.82308700	1.20269200
C	0.31396500	-2.67773000	2.51223700
C	0.50879800	-1.52883100	3.31362400

C	-0.16528100	-1.35499000	4.51638900
C	-1.05289600	-2.34202900	4.98178600
C	-1.21669000	-3.52207100	4.23178200
C	-0.53860700	-3.67791900	3.02102500
O	-1.69385900	-2.07398100	6.14465100
C	-2.63639700	-3.00746000	6.64610400
C	1.57127200	-4.01754600	0.80432800
C	2.24493100	-4.14216600	-0.41439500
C	2.36286600	-2.98748100	-1.26940100
C	3.08189300	-2.96054100	-2.49187700
C	3.17195300	-1.80938600	-3.28039300
C	2.53576500	-0.62595600	-2.86654600
C	2.58093800	0.61906000	-3.56986400
C	1.91112500	1.71094200	-3.09479600
C	1.13664300	1.62423400	-1.88350100
C	0.33545700	2.77218800	-1.41711700
C	-0.95624600	2.59675800	-0.89352100
C	-1.67386100	3.65560600	-0.33857200
C	-1.09792000	4.93718500	-0.29943900
C	0.16434800	5.14368300	-0.89178400
C	0.86258200	4.08056800	-1.44922600
O	-1.67119300	6.01369400	0.27731200
C	-2.90937400	5.85995300	0.95809800
N	0.43560500	1.80379200	1.74780500
C	-0.72889000	2.00513800	2.43094400
C	-0.95178800	3.15291800	3.23010000
C	-2.17421500	3.27684100	3.96854800
C	-3.12521200	2.29462200	3.91578500
C	0.07396200	4.12766000	3.25589500
C	1.24376600	3.90705500	2.56018600
C	1.43095600	2.70861500	1.82286000
C	2.71735200	2.43779300	1.15048600
C	3.43127200	3.47525500	0.51107700
C	4.60160200	3.21248000	-0.18708100
C	5.11482200	1.90062700	-0.25662000
C	4.44682700	0.86641900	0.42236400
C	3.26899000	1.14582700	1.11339600
O	6.23737800	1.73531200	-0.98210400
C	6.78197800	0.43031000	-1.13582600
H	-0.92211200	-5.84310800	-4.20629900
H	0.00311500	-4.44098500	-3.57746500
H	-1.59302800	-4.18795700	-4.36586800
H	-2.76202900	-5.39250600	-0.20448600
H	-3.16848600	-3.62278700	1.48506300
H	-1.06181900	-2.44706700	-2.87250300
H	-1.46463700	-0.70531000	-1.20601500
H	-4.42098300	-1.72693000	1.97850800
H	-4.81473100	0.14561400	3.56871700
H	1.20022300	-0.75378300	2.97269800
H	-0.02162600	-0.45388900	5.11680500
H	-1.89110400	-4.31053800	4.56713600
H	-0.71731500	-4.57918800	2.43130900
H	-3.03658500	-2.57842900	7.57516500
H	-2.16944600	-3.98191700	6.87473200
H	-3.46830900	-3.16730700	5.93712700
H	1.53215700	-4.86548700	1.49261800
H	2.72401500	-5.08165900	-0.69993900
H	3.58566300	-3.87733100	-2.81016900
H	3.73904300	-1.81465900	-4.21405500
H	3.15305200	0.68289500	-4.49964500
H	1.92278100	2.65583900	-3.63945600
H	-1.40497300	1.60086100	-0.89767400
H	-2.66380800	3.46895700	0.07628700
H	0.59143400	6.14866700	-0.87183800
H	1.85555200	4.25606900	-1.86666200
H	-3.15141700	6.84224900	1.38604700
H	-2.83358700	5.11973500	1.77332400
H	-3.71834800	5.55869200	0.27004700
H	-2.32141700	4.16798000	4.58271200
H	-4.05038000	2.37865000	4.49056900
H	-0.06203500	5.03813800	3.84410900
H	2.05705800	4.63142900	2.60672800
H	3.03932400	4.49329200	0.52167000
H	5.13476900	4.00955300	-0.70959000
H	4.82924800	-0.15373400	0.41612300

H	2.76661700	0.32812500	1.63517100
H	7.66079900	0.53612400	-1.78602100
H	6.05985700	-0.25549400	-1.61196600
H	7.09994800	0.00611600	-0.16761700

MECP 2

E (SMD/B3LYP-D3/def2-SVP) = -4164.1868887300

C	-1.01601500	-4.86650200	-3.74428000
O	-1.69831400	-5.09911500	-2.51789300
C	-1.89025800	-4.07381400	-1.66891200
C	-2.47460000	-4.39281700	-0.42830700
C	-2.64835500	-3.40858700	0.53517900
C	-1.55467200	-2.73901600	-1.95836000
C	-1.72640800	-1.76159300	-0.97693600
C	-2.22908500	-2.08596400	0.29563300
C	-2.38821000	-1.05455800	1.32598600
C	-3.61451000	-0.96917900	2.05398600
C	-3.85409400	0.05778700	2.92037200
C	-2.90749000	1.09897900	3.06391300
C	-1.70006700	0.95317900	2.33854300
N	-1.42756700	-0.15193000	1.58703200
Cu	0.28420800	0.12176700	0.58384700
N	1.27228100	0.55781300	-1.10074400
C	1.93521800	-0.58524700	-1.56445900
C	1.75190600	-1.74029100	-0.81837900
N	0.99058200	-1.61698700	0.35772900
C	0.83359900	-2.81746900	1.20896000
C	0.26506900	-2.65608300	2.54555600
C	0.49679700	-1.52783000	3.36970500
C	-0.14543000	-1.36731200	4.59408100
C	-1.04306700	-2.34027000	5.05972100
C	-1.24863000	-3.49739700	4.29569200
C	-0.60682400	-3.63179600	3.06052000
O	-1.68572400	-2.07431500	6.24197900
C	-2.64779200	-3.00148500	6.69038800
C	1.41734400	-3.98188100	0.78170300
C	2.13343100	-4.13757100	-0.45140300
C	2.33083900	-2.95491900	-1.26127600
C	3.08689500	-2.92264600	-2.46994500
C	3.24099900	-1.77955400	-3.24371800
C	2.64476900	-0.56786900	-2.82921800
C	2.60253500	0.62804100	-3.55537200
C	1.85607100	1.71565200	-3.10424900
C	1.17875300	1.65288200	-1.84946600
C	0.35956900	2.80228300	-1.40005300
C	-0.94945900	2.62319500	-0.92209600
C	-1.68164600	3.67628100	-0.37917000
C	-1.11137100	4.95939400	-0.31273200
C	0.16160700	5.17205000	-0.88140300
C	0.87897000	4.11628000	-1.42127600
O	-1.69601500	6.02600300	0.26813000
C	-2.92811300	5.85172700	0.95602400
N	0.36665400	1.87522800	1.58153100
C	-0.72568000	1.99529400	2.38749300
C	-0.92943600	3.11668500	3.22130700
C	-2.14690900	3.22761500	3.96826800
C	-3.10981000	2.25818700	3.87968900
C	0.09608300	4.08488800	3.24526300
C	1.25789200	3.87147900	2.53859600
C	1.40969600	2.71101200	1.71831300
C	2.69967100	2.44453400	1.07115100
C	3.42905700	3.48857500	0.45836900
C	4.62673400	3.21839900	-0.19309000
C	5.14115100	1.91270300	-0.23264600
C	4.46932300	0.87695800	0.45186900
C	3.25919900	1.16175200	1.09265300
O	6.27076200	1.73516900	-0.93648300
C	6.79161500	0.42125100	-1.10865400
H	-0.91946900	-5.84377300	-4.23411100
H	-0.01024100	-4.44636800	-3.57074600
H	-1.58543200	-4.18653700	-4.40133800
H	-2.75600800	-5.42792800	-0.22909900
H	-3.07085700	-3.67824100	1.50383200
H	-1.14782700	-2.44998000	-2.92326400

H	-1.46863000	-0.73204200	-1.22250500
H	-4.36618600	-1.73887600	1.87338800
H	-4.79807800	0.11328300	3.47594000
H	1.17389800	-0.73936200	3.02201800
H	0.02299800	-0.47342300	5.20093600
H	-1.93330600	-4.28035000	4.61847900
H	-0.82219800	-4.51109800	2.44654500
H	-3.06676400	-2.59604700	7.62156800
H	-2.20374600	-3.99270800	6.90064000
H	-3.46764700	-3.13612700	5.95915900
H	1.41247000	-4.81033400	1.50205200
H	2.59932800	-5.07915800	-0.73780400
H	3.53755300	-3.86036400	-2.82053900
H	3.78131100	-1.81793900	-4.19758600
H	3.12912400	0.68618000	-4.51245400
H	1.74427700	2.62087700	-3.69551600
H	-1.40366900	1.62781700	-0.94615200
H	-2.68110000	3.48428600	0.01464800
H	0.58182800	6.18218500	-0.85327300
H	1.87742400	4.29124700	-1.81768500
H	-3.17877600	6.82598100	1.39630300
H	-2.83612500	5.10363700	1.76286400
H	-3.73793800	5.54774400	0.27035900
H	-2.29070100	4.10944400	4.59760300
H	-4.04721100	2.34450500	4.43192600
H	-0.01973100	4.98470500	3.85552000
H	2.08622600	4.56619300	2.60206100
H	3.03377300	4.50233700	0.45312300
H	5.17380100	4.01519300	-0.70425300
H	4.85730500	-0.13341500	0.47907100
H	2.74720500	0.34821700	1.61320500
H	7.66713500	0.51894100	-1.76227200
H	6.05180800	-0.24306400	-1.58815400
H	7.10479700	-0.01850800	-0.14622200

MECP3E
(SMD/B3LYP-D3/def2-SVP) = -4164.1628808900

C	-1.17040900	-4.65148500	-4.01284200
O	-1.77000900	-4.94849700	-2.75730500
C	-1.93488900	-3.96546900	-1.85370900
C	-2.46102600	-4.34911000	-0.60369900
C	-2.62590000	-3.41175000	0.40621200
C	-1.63001700	-2.61122300	-2.08799100
C	-1.78558400	-1.68531400	-1.06112900
C	-2.24873800	-2.06727000	0.21030000
C	-2.38097700	-1.07889900	1.29305900
C	-3.58449100	-1.03216900	2.05331300
C	-3.81756800	-0.02092200	2.95397700
C	-2.86857000	1.02207100	3.09775000
C	-1.67845700	0.90088000	2.34849600
N	-1.41308800	-0.18039700	1.55494100
Cu	0.29830400	0.09935100	0.56717100
N	1.34146300	0.57528400	-0.97001100
C	1.87257200	-0.61336800	-1.55327700
C	1.65673500	-1.77766900	-0.89407400
N	0.80109500	-1.73150000	0.24221800
C	0.86092500	-2.77290300	1.15358100
C	0.28964000	-2.59109700	2.51444200
C	0.63676400	-1.47920400	3.30872700
C	0.07352600	-1.27268400	4.56032400
C	-0.86677700	-2.18640500	5.07352300
C	-1.19128300	-3.32646900	4.31895500
C	-0.60964900	-3.51848000	3.06071800
O	-1.39720200	-1.88307900	6.28015400
C	-2.38239800	-2.73769500	6.83914500
C	1.44692500	-3.96960700	0.79894100
C	2.11811500	-4.12234800	-0.47673700
C	2.27316300	-3.02753800	-1.29302900
C	3.04929700	-2.96571600	-2.51851300
C	3.20757600	-1.80498500	-3.22262700
C	2.61061400	-0.54975400	-2.79764600
C	2.64679000	0.64429700	-3.47753300
C	1.85366900	1.76101900	-2.98964700
C	1.17711400	1.68585800	-1.79500400

C	0.29230100	2.79982100	-1.35993400
C	-1.02375400	2.57318200	-0.92669600
C	-1.82582600	3.59725300	-0.41905700
C	-1.31913300	4.90616300	-0.35214400
C	-0.03020900	5.16544700	-0.85452700
C	0.75735100	4.12975500	-1.34760400
O	-1.98238100	5.95927700	0.17941100
C	-3.22259700	5.73594200	0.83248000
N	0.38941400	1.82393400	1.58608600
C	-0.69937300	1.93888200	2.40252600
C	-0.89051600	3.03959600	3.26487800
C	-2.09274000	3.12966400	4.03732200
C	-3.05563900	2.16105900	3.94527400
C	0.13994800	4.01220800	3.28820100
C	1.28514600	3.80795000	2.55682200
C	1.43525100	2.65798500	1.72946000
C	2.73469200	2.37694600	1.10068400
C	3.49483300	3.39766800	0.49359900
C	4.70667000	3.10847100	-0.11853500
C	5.20967400	1.79132900	-0.12110500
C	4.49281200	0.77671500	0.54130100
C	3.27249100	1.07887400	1.13516800
O	6.37264300	1.59712400	-0.76877600
C	6.88580700	0.27607200	-0.89924000
H	-1.10035600	-5.60451100	-4.55425400
H	-0.15779300	-4.23113200	-3.88653500
H	-1.78522700	-3.94814200	-4.60036600
H	-2.71348000	-5.39920100	-0.44320100
H	-3.01001500	-3.73308500	1.37539000
H	-1.25429800	-2.27520600	-3.05366100
H	-1.54662000	-0.63965100	-1.26555100
H	-4.33327200	-1.80375600	1.87730300
H	-4.74563900	0.01122800	3.52926600
H	1.34970300	-0.74658000	2.92328400
H	0.33471300	-0.39640400	5.15777400
H	-1.90773000	-4.05967000	4.69012800
H	-0.89163500	-4.39557600	2.47654000
H	-2.67372500	-2.28993900	7.79927800
H	-1.98829500	-3.75303100	7.02146800
H	-3.27391000	-2.80984500	6.19097300
H	1.45557100	-4.78175600	1.52660900
H	2.56376600	-5.08246800	-0.74960500
H	3.52868400	-3.88555900	-2.86834700
H	3.79325900	-1.80451200	-4.14749600
H	3.19681100	0.73112400	-4.41789600
H	1.73370600	2.65687900	-3.60025600
H	-1.42552600	1.55586700	-0.94220300
H	-2.82819300	3.36189700	-0.06153600
H	0.34903200	6.18937300	-0.81388200
H	1.77080100	4.34384200	-1.69046900
H	-3.54123000	6.70725200	1.23537200
H	-3.11932500	5.01747400	1.66552300
H	-3.99559800	5.36777600	0.13519100
H	-2.22457600	3.99312000	4.69245700
H	-3.98121800	2.23198300	4.52015900
H	0.02996900	4.89880900	3.91670700
H	2.11485400	4.51141300	2.61660900
H	3.10999500	4.41795800	0.46100000
H	5.27978900	3.88746400	-0.62534700
H	4.86360300	-0.24655000	0.57914600
H	2.71604000	0.27869300	1.62630900
H	7.80490900	0.35924700	-1.49452100
H	6.17135100	-0.38172700	-1.42375600
H	7.13132600	-0.16400100	0.08256400

TS$_{C\text{-}P1}$
E (SMD/B3LYP-D3/def2-SVP) = -5310.458549 au
H (SMD/B3LYP-D3/def2-SVP) = -5309.413533 au
G (SMD/B3LYP-D3/def2-SVP) = -5309.577295 au
E (SMD/B3LYP-D3/def2-TZVP//SMD/B3LYP-D3/def2-SVP) = -5314.344506 au

C	-0.29421600	-0.93173900	6.85032600
O	-0.81319900	-2.07985800	6.19272600
C	-0.43741100	-2.33949200	4.92443100
C	-1.02571000	-3.46545900	4.31500600

C	-0.71564400	-3.80066100	3.00367600
C	0.48230900	-1.56917500	4.19155500
C	0.77912100	-1.91692600	2.87467200
C	0.18496900	-3.02272400	2.24325100
C	0.45690900	-3.33801700	0.82083700
C	0.40677100	-4.68841400	0.37316300
C	0.71404000	-4.98843800	-0.93357600
C	1.10647500	-3.95570900	-1.81777200
C	1.11791700	-2.63123900	-1.30238900
N	0.77223200	-2.34951800	-0.02301400
Cu	0.60858400	-0.06290500	0.15591100
N	2.42306900	0.61891100	1.11448500
C	2.13176900	1.77755600	1.76455100
C	0.74470500	2.09058900	2.05420100
N	-0.23285800	1.21821400	1.67290800
C	-1.51563400	1.46133600	2.00493900
C	-2.58133100	0.46739600	1.75307700
C	-2.28701600	-0.90509500	1.66021900
C	-3.27968600	-1.85527700	1.48870400
C	-4.62844500	-1.46458500	1.38405800
C	-4.94955800	-0.09811000	1.46999500
C	-3.93507000	0.84236000	1.65794600
O	-5.52526400	-2.45306400	1.19667100
C	-6.91214600	-2.14252000	1.15502400
C	-1.86370400	2.67111800	2.67215900
C	-0.89912900	3.58666100	3.01096100
C	0.45725000	3.30844300	2.72664400
C	1.50499300	4.21397600	3.09022400
C	2.80697800	3.92285700	2.80682700
C	3.14585900	2.69828200	2.14834700
C	4.48350800	2.36477700	1.83638400
C	4.75573000	1.17859200	1.19540500
C	3.69609300	0.30384000	0.84477500
C	4.03657000	-0.96287600	0.14954400
C	3.70407000	-2.21536200	0.67704900
C	4.03011100	-3.39704300	0.00642800
C	4.68604300	-3.33550600	-1.23306500
C	5.06648500	-2.08141400	-1.75020800
C	4.75870100	-0.91694300	-1.06000900
O	4.99056900	-4.41089900	-1.99218500
C	4.59099300	-5.70165000	-1.55586300
N	1.63107200	-0.29208300	-1.67628600
C	1.58736900	-1.55685000	-2.16180200
C	2.04565100	-1.86778900	-3.47048700
C	1.99386900	-3.21523400	-3.95606300
C	1.53413100	-4.22303600	-3.15895100
C	2.57708900	-0.80864500	-4.24272500
C	2.64608300	0.45854500	-3.70850000
C	2.14705000	0.70296300	-2.40235400
C	2.24906600	2.07199400	-1.83299300
C	3.49609000	2.73004500	-1.84737100
C	3.64533200	3.99502700	-1.29128000
C	2.54036100	4.65204200	-0.71824000
C	1.28605600	4.02325400	-0.73227100
C	1.15022500	2.74660200	-1.28106400
O	2.77765700	5.86927100	-0.17964700
C	1.68628600	6.63114800	0.31543900
H	-0.75076700	-0.91378900	7.84915600
H	-0.56011900	-0.00357000	6.31488700
H	0.80325400	-0.98478500	6.95736200
H	-1.74404200	-4.05381900	4.89001600
H	-1.21910200	-4.65754400	2.55234000
H	0.96326900	-0.69445500	4.62885400
H	1.47032700	-1.28893900	2.31852800
H	0.15847000	-5.48453200	1.07483600
H	0.69483300	-6.02140200	-1.28932700
H	-1.25493800	-1.22755100	1.73666500
H	-3.03333300	-2.91696800	1.42768000
H	-4.22355900	1.89331100	1.69929700
H	-7.43655500	-3.09466100	0.99717000
H	-7.15339400	-1.45901600	0.32667800
H	-7.25170300	-1.69601200	2.10602500
H	-2.90117400	2.85597100	2.94542700
H	-1.16328900	4.51594100	3.52146500
H	1.23235600	5.14264100	3.59641900

H	3.61156800	4.61265000	3.07018400
H	5.28571300	3.05435300	2.10990400
H	5.77934500	0.88944100	0.95492900
H	3.18508000	-2.28066300	1.63217800
H	3.74216400	-4.35230700	0.44394400
H	5.58420600	-2.04737800	-2.71097400
H	5.04074700	0.04963300	-1.48261200
H	4.89834300	-6.40429200	-2.34235000
H	3.49791900	-5.76512100	-1.42382900
H	5.08587900	-5.98295100	-0.60958000
H	2.34625500	-3.41506100	-4.97067100
H	1.50760600	-5.25497600	-3.51643600
H	2.93782300	-1.01085700	-5.25427400
H	3.05873300	1.28890200	-4.28263600
H	4.36766900	2.22731500	-2.27307900
H	4.61748600	4.49241800	-1.27501800
H	0.41312500	4.51000100	-0.30419600
H	0.17002500	2.26649200	-1.27525100
H	2.10797200	7.57873000	0.67743500
H	1.17820700	6.12316800	1.15170000
H	0.94897200	6.84456700	-0.47828700
Cl	-1.27910000	0.05726800	-1.23825400
H	-5.97988000	0.24302000	1.37442900
C	-3.78188500	0.37759700	-2.71485500
C	-4.26144100	-0.97093500	-2.28657000
H	-3.77358100	-1.77142000	-2.86031000
H	-4.03806600	-1.14143400	-1.21979300
C	-4.27569100	1.62299600	-2.05626700
H	-4.48927700	1.41174700	-0.99682100
H	-5.26227800	1.89459300	-2.49346300
C	-3.36353500	2.82691900	-2.16683600
C	-2.89886100	3.27879000	-3.41297700
C	-2.97843300	3.53320300	-1.01641400
C	-2.07249200	4.40386200	-3.50625900
H	-3.18863100	2.74615800	-4.32340400
C	-2.15826400	4.66208200	-1.10608200
H	-3.31672800	3.18919800	-0.03671900
C	-1.70058300	5.10281900	-2.35228100
H	-1.71921400	4.73764800	-4.48596200
H	-1.86643100	5.18983000	-0.19415300
C	-5.75188400	-1.18861200	-2.43351900
F	-6.11592500	-2.41858300	-2.01622400
F	-6.47904400	-0.30624200	-1.70355900
F	-6.17076600	-1.06348200	-3.70899400
H	-3.10667800	0.44639900	-3.56877700
H	-1.05149000	5.97936600	-2.42348500

TS$_{A-P2}$
E (SMD/B3LYP-D3/def2-SVP) = -5858.844543 au
H (SMD/B3LYP-D3/def2-SVP) = -5857.785004 au
G (SMD/B3LYP-D3/def2-SVP) = -5857.958862 au
E (SMD/B3LYP-D3/def2-TZVP//SMD/B3LYP-D3/def2-SVP) = -5863.085531 au

C	-2.60083300	-6.32175000	2.29826400
O	-1.39772700	-5.61661300	2.02262800
C	-1.32322500	-4.29989100	2.30293100
C	-0.12842500	-3.64874800	1.94273300
C	0.02243700	-2.29147000	2.16662000
C	-2.35159300	-3.55562900	2.90840800
C	-2.17801400	-2.18914800	3.13456400
C	-0.99267200	-1.52486500	2.76710000
C	-0.81816900	-0.08377500	3.04054600
C	-1.48861700	0.46369500	4.17392900
C	-1.35698600	1.78974700	4.49738100
C	-0.52564000	2.61771000	3.70962000
C	0.13717900	2.01399600	2.60927400
N	-0.03047600	0.69830700	2.27768300
Cu	0.71108000	0.44940400	0.28521600
N	0.54511200	1.06028700	-1.70868300
C	1.22867300	0.28120900	-2.57796300
C	2.09438100	-0.75550100	-2.05550000
N	2.22663500	-0.87817200	-0.71357600
C	3.06730300	-1.78216400	-0.20586300
C	3.23758400	-1.86648700	1.26331300
C	3.15838200	-0.72329900	2.08717900

C	3.24744100	-0.82285200	3.46825800
C	3.41705100	-2.07846600	4.08356500
C	3.52021400	-3.22633400	3.27810800
C	3.43740700	-3.10933000	1.88945000
O	3.46730500	-2.08215600	5.43121700
C	3.61428300	-3.31616700	6.12046300
C	3.80350800	-2.65023300	-1.05806100
C	3.66853900	-2.54873700	-2.42348300
C	2.80842500	-1.56973200	-2.97185600
C	2.67476900	-1.36290800	-4.38288500
C	1.87888700	-0.36541800	-4.87102100
C	1.15065800	0.48769800	-3.97966000
C	0.37062900	1.57788200	-4.42963500
C	-0.28164700	2.38008500	-3.51783200
C	-0.20447300	2.07924500	-2.13457900
C	-0.94914400	2.89485000	-1.14501900
C	-1.78789000	2.29629200	-0.19364200
C	-2.45945700	3.05345600	0.76529200
C	-2.29816100	4.44814800	0.78813200
C	-1.48811300	5.06395300	-0.18541600
C	-0.83221800	4.29813100	-1.14247800
O	-2.87332200	5.26738000	1.69529800
C	-3.72458100	4.71083200	2.68657200
N	1.66222900	2.26701000	0.75165200
C	0.99823500	2.84078700	1.79359000
C	1.11789100	4.22634300	2.08697500
C	0.41337200	4.79724600	3.19372800
C	-0.37331700	4.01386900	3.98537900
C	1.93796200	5.00472200	1.23899400
C	2.61487200	4.39896100	0.20638700
C	2.48663800	3.00274500	-0.00733000
C	3.30801000	2.38313700	-1.07343800
C	3.24106300	2.87156000	-2.39311900
C	4.00885900	2.30287400	-3.40007600
C	4.89405900	1.24630500	-3.10896700
C	5.02137100	0.80034500	-1.78348700
C	4.22260900	1.36242900	-0.78608400
O	5.56823500	0.72878800	-4.15784200
C	6.46747500	-0.34963900	-3.94378400
H	-2.43362000	-7.35735900	1.97221900
H	-3.45153900	-5.90138600	1.73663200
H	-2.83739700	-6.31920600	3.37660900
H	0.65470300	-4.22131700	1.44665700
H	0.94138000	-1.80340300	1.86084600
H	-3.29549000	-4.02312300	3.18706000
H	-3.00953500	-1.63657900	3.57549900
H	-2.09196300	-0.19017800	4.80143200
H	-1.87574600	2.21114300	5.36158600
H	3.00611400	0.25809500	1.64326800
H	3.17654500	0.06547800	4.09965000
H	3.64560600	-4.21399300	3.72117400
H	3.48416600	-4.02078000	1.28984400
H	3.61849900	-3.07381300	7.19184300
H	4.56332200	-3.81722500	5.86140500
H	2.77471700	-4.00144100	5.90987500
H	4.49572500	-3.37180100	-0.62403900
H	4.23842400	-3.19913500	-3.09144200
H	3.24229700	-2.00830100	-5.05746100
H	1.79286800	-0.18881600	-5.94573600
H	0.29788400	1.77903000	-5.50138100
H	-0.88710600	3.22615000	-3.84229300
H	-1.91789800	1.21467800	-0.19967400
H	-3.09857000	2.54691800	1.48707400
H	-1.37292600	6.14952300	-0.15737800
H	-0.19250500	4.79082500	-1.87837500
H	-4.05560000	5.54743700	3.31683300
H	-3.19478900	3.97553100	3.31525400
H	-4.60887900	4.22767300	2.23504100
H	0.52118800	5.86721000	3.38372000
H	-0.91513000	4.43477600	4.83497900
H	2.03454600	6.07743700	1.42246600
H	3.27935400	4.96919800	-0.44352600
H	2.54541300	3.67736000	-2.63691100
H	3.92554400	2.64459000	-4.43368200
H	5.71208500	-0.00043300	-1.52256500

H	4.32299000	0.99860900	0.23568400
H	6.86941500	-0.61835300	-4.93013500
H	5.95613700	-1.22642400	-3.51272100
H	7.30218400	-0.05760700	-3.28263900
S	-1.45808800	-1.70018900	-1.20463400
O	-0.77759800	-0.86642000	-0.12238900
O	-1.46096800	-1.05121500	-2.53705600
Cl	0.19114900	-3.56424400	-1.54582900
C	-3.86365000	-1.42213800	-0.58295100
C	-4.20597400	-2.77066900	-0.02405300
H	-3.45557000	-3.08143100	0.71608400
H	-5.17246900	-2.72152600	0.51289500
C	-4.51015800	-0.91371600	-1.84045500
H	-5.55377000	-1.28199800	-1.85630300
H	-4.02817700	-1.37926600	-2.71814200
C	-4.52147900	0.59131600	-1.99013300
C	-3.78328000	1.23324600	-2.99513800
C	-5.29985400	1.37663100	-1.12150100
C	-3.83585500	2.62447500	-3.14048300
H	-3.14964900	0.63636600	-3.65449600
C	-5.34560000	2.76664100	-1.25819000
H	-5.87838700	0.88850800	-0.33118800
C	-4.61583500	3.39601100	-2.27413000
H	-3.25548700	3.10759900	-3.93017400
H	-5.95305500	3.36185000	-0.57085300
C	-4.34144400	-3.90482200	-1.01229600
F	-4.52610400	-5.07693500	-0.36562200
F	-5.39569300	-3.75565700	-1.84338600
F	-3.25345400	-4.05599100	-1.79262000
H	-3.59904400	-0.68534700	0.18314600
H	-4.64800200	4.48328700	-2.38248400

P2(S2)
E (SMD/B3LYP-D3/def2-SVP) = -1694.591084 au
H (SMD/B3LYP-D3/def2-SVP) = -1694.381870 au
G (SMD/B3LYP-D3/def2-SVP) = -1694.445885 au
E (SMD/B3LYP-D3/def2-TZVP//SMD/B3LYP-D3/def2-SVP) = -1695.907136 au

C	2.92189700	1.21332400	0.35464100
C	3.75327200	2.47201800	0.09907400
H	3.41332200	3.30970000	0.72349900
H	4.81647400	2.29442400	0.31259600
C	3.60938100	-0.09346200	-0.09139200
H	4.50252100	-0.26244000	0.52961800
H	3.97634200	0.11799500	-1.10722700
C	2.72785800	-1.32013800	-0.13086800
C	1.65118000	-1.38567800	-1.03072800
C	2.96821000	-2.41054900	0.71688600
C	0.82830700	-2.51410000	-1.07635200
H	1.45811600	-0.54182400	-1.70035500
C	2.14938400	-3.54401000	0.67010200
H	3.79961200	-2.36650200	1.42593400
C	1.07549000	-3.59795400	-0.22453400
H	-0.00663900	-2.55019700	-1.78143900
H	2.34942000	-4.38604500	1.33830400
C	3.63886300	2.97072000	-1.32597400
F	4.28587900	4.13938400	-1.46692300
F	4.16619000	2.11829000	-2.22958000
F	2.35661200	3.17406400	-1.68629000
S	2.58753500	1.07841300	2.16508900
O	1.70355800	-0.04560200	2.44634700
O	3.83112200	1.25656500	2.90967100
Cl	1.39266200	2.86415500	2.49654600
H	1.91285600	1.30728200	-0.07315200
H	0.43329200	-4.48213900	-0.25992900

TS$_{S2}$
E (SMD/B3LYP-D3/def2-SVP) = -1694.713179 au
H (SMD/B3LYP-D3/def2-SVP) = -1694.508713 au
G (SMD/B3LYP-D3/def2-SVP) = -1694.577264 au
E (SMD/B3LYP-D3/def2-TZVP//SMD/B3LYP-D3/def2-SVP) = -1696.033884 au

C	2.87235400	1.23447300	-0.19360200
C	3.56039400	2.56253900	-0.30357900
H	3.11494300	3.30112500	0.38180100

H	4.63074900	2.47799200	-0.05698200
C	3.61094900	-0.01251300	-0.58487800
H	4.46340400	-0.14499600	0.10659600
H	4.05886700	0.14712900	-1.58510200
C	2.75283800	-1.25793500	-0.61093400
C	2.05931500	-1.61409700	-1.77983700
C	2.60245800	-2.06206100	0.53148400
C	1.23824400	-2.74633100	-1.81186200
H	2.16707600	-0.99490900	-2.67581800
C	1.78348600	-3.19616500	0.50077300
H	3.12027600	-1.78238800	1.45128500
C	1.09907000	-3.54330600	-0.66971300
H	0.70877500	-3.00882400	-2.73217200
H	1.67845100	-3.81224400	1.39847700
C	3.47791100	3.17346800	-1.68641900
F	4.09211400	4.37260700	-1.73730500
F	4.05887900	2.39855900	-2.62986300
F	2.20213100	3.36851900	-2.07914200
S	2.85418500	1.03295000	2.36540800
O	1.57817400	0.33111400	2.66615000
O	4.06729400	0.18642200	2.56868400
Cl	3.05567300	2.78390400	4.30579100
H	1.78071400	1.23904900	-0.27890800
H	0.46051800	-4.43076400	-0.69204300

E(S2)
E (SMD/B3LYP-D3/def2-SVP) = -1694.7302602 au
H (SMD/B3LYP-D3/def2-SVP) = -1694.522829 au
G (SMD/B3LYP-D3/def2-SVP) = -1694.591231 au
E (SMD/B3LYP-D3/def2-TZVP//SMD/B3LYP-D3/def2-SVP) = -1696.04874954 au

C	2.90269000	1.19374900	0.31929400
C	3.75938300	2.44871600	0.16594600
H	3.40040500	3.21741500	0.87043400
H	4.81784900	2.23859100	0.37918000
C	3.52563500	-0.05991900	-0.31286300
H	4.48863800	-0.24387100	0.18828800
H	3.75243300	0.19464500	-1.35939700
C	2.67582800	-1.30851600	-0.28712900
C	1.67810500	-1.51006300	-1.25460000
C	2.86189400	-2.28889300	0.70048500
C	0.88428900	-2.66167100	-1.23696200
H	1.52416700	-0.75435400	-2.03119500
C	2.07687200	-3.44553600	0.71668500
H	3.61627100	-2.12482800	1.47391200
C	1.08379100	-3.63581100	-0.25165500
H	0.11205000	-2.80214700	-1.99859600
H	2.23653600	-4.20002700	1.49223700
C	3.68502000	3.08433500	-1.19791700
F	4.35811400	4.25326100	-1.22070100
F	4.21287000	2.32221400	-2.18415100
F	2.41436900	3.35608700	-1.56425700
S	2.63405500	0.90772100	2.17047200
O	1.46367400	-0.02361200	2.27394800
O	3.96271300	0.38704600	2.65655600
Cl	1.69871700	3.58334300	2.65412500
H	1.88010800	1.37789800	-0.04092000
H	0.46852800	-4.53977500	-0.23939200

B
E (SMD/B3LYP-D3/def2-SVP) = -4164.094008 au
H (SMD/B3LYP-D3/def2-SVP) = -4163.240191 au
G (SMD/B3LYP-D3/def2-SVP) = -4163.372800 au
E (SMD/B3LYP-D3/def2-TZVP//SMD/B3LYP-D3/def2-SVP) = -4167.047594 au

C	-3.10201200	-4.88325300	2.93725000
O	-1.80832800	-5.29596400	2.51200200
C	-0.84672400	-4.37115600	2.33035500
C	0.38389900	-4.83901800	1.82355400
C	1.41214500	-3.95088200	1.53883200
C	-0.99935000	-3.00154100	2.61012500
C	0.03815900	-2.12182800	2.30951900
C	1.23900000	-2.56458500	1.73029900
C	2.26640400	-1.59656300	1.29632300
C	3.64631700	-1.81551100	1.55417900

C	4.57405000	-0.84835900	1.23132600
C	4.15140200	0.36624800	0.63497600
C	2.77153900	0.48802600	0.34309600
N	1.87502400	-0.48278500	0.66075100
Cu	-0.00532500	0.06009500	0.03186000
N	-1.83988400	0.57999300	0.79297300
C	-2.75909900	-0.37467500	0.48899900
C	-2.31583400	-1.54360600	-0.22802100
N	-1.01279600	-1.60679800	-0.61541800
C	-0.54993500	-2.66814700	-1.29270700
C	0.85203800	-2.68804800	-1.75914200
C	1.48295300	-1.53186300	-2.26594100
C	2.81779800	-1.54499100	-2.63967900
C	3.57780100	-2.72530000	-2.51791800
C	2.95397600	-3.89947300	-2.05570100
C	1.60713500	-3.87238500	-1.69451200
O	4.87486300	-2.63699400	-2.86647900
C	5.70953300	-3.78336000	-2.75713300
C	-1.41441000	-3.76663700	-1.55761100
C	-2.73451200	-3.72588400	-1.16793800
C	-3.23723600	-2.58390900	-0.49680700
C	-4.60498300	-2.44589400	-0.08927600
C	-5.03447300	-1.31682500	0.55339000
C	-4.12102900	-0.25427500	0.85742200
C	-4.50188700	0.93713600	1.52420200
C	-3.55332700	1.89064200	1.82407200
C	-2.19298100	1.67907300	1.47413900
C	-1.14394600	2.64776300	1.84706100
C	0.09777900	2.20941200	2.33430300
C	1.14881900	3.09515400	2.56198800
C	0.96961800	4.46386700	2.29389200
C	-0.29681600	4.92624300	1.87905800
C	-1.33862200	4.03306500	1.67034300
O	1.93796800	5.39296800	2.40260800
C	3.25407800	4.98961300	2.76223000
N	0.96160800	1.74016000	-0.62289900
C	2.28183300	1.68034100	-0.30037400
C	3.17525000	2.74552000	-0.56392000
C	4.56340300	2.60564500	-0.23211300
C	5.03692500	1.45289100	0.33307700
C	2.62368800	3.91136200	-1.15016300
C	1.28759500	3.94529300	-1.48406400
C	0.45584200	2.81751200	-1.24187300
C	-0.95524800	2.80948700	-1.67914600
C	-1.75723800	3.96533400	-1.56202700
C	-3.10131900	3.93777100	-1.90553700
C	-3.69032200	2.75531500	-2.39997600
C	-2.89082000	1.61191300	-2.57694900
C	-1.54445700	1.65341700	-2.22038500
O	-5.00624400	2.81486000	-2.67633500
C	-5.67743900	1.64755500	-3.13491800
H	-3.72272600	-5.78895000	2.96049100
H	-3.54619700	-4.15828700	2.23309500
H	-3.07569700	-4.43908200	3.94715700
H	0.49549900	-5.90831200	1.63190900
H	2.34063400	-4.32878100	1.10805100
H	-1.92002900	-2.61119300	3.04224700
H	-0.09704400	-1.05784500	2.51363300
H	3.95065100	-2.73795600	2.04973800
H	5.63353600	-0.99703300	1.45346100
H	0.91404800	-0.60542200	-2.36610600
H	3.30260100	-0.64550800	-3.02419500
H	3.51078600	-4.83111400	-1.95954500
H	1.15217100	-4.78587700	-1.30880500
H	6.71006800	-3.46910100	-3.08326300
H	5.35938900	-4.60385300	-3.40693800
H	5.76614500	-4.14432100	-1.71558700
H	-1.02539600	-4.62560300	-2.10448400
H	-3.40532300	-4.56042900	-1.38502800
H	-5.29652400	-3.26146600	-0.31183600
H	-6.07843700	-1.20612600	0.85501900
H	-5.54626700	1.08055500	1.81153900
H	-3.82383600	2.79823700	2.36463200
H	0.25671200	1.14534200	2.52326300
H	2.10124200	2.70949000	2.92342300

H	-0.42715800	5.99485700	1.69578800
H	-2.29859600	4.40587400	1.30944100
H	3.87112700	5.89791500	2.74407000
H	3.66376500	4.25941400	2.04350200
H	3.28187400	4.55552400	3.77651400
H	5.23446900	3.43958100	-0.44919400
H	6.09624700	1.34162300	0.57519000
H	3.26940200	4.76928400	-1.35149100
H	0.86142700	4.82180700	-1.97226100
H	-1.33341200	4.88337300	-1.15265300
H	-3.73061800	4.82180200	-1.78344200
H	-3.30384800	0.69019300	-2.98523100
H	-0.93837300	0.75587500	-2.36014500
H	-6.73200000	1.92702000	-3.26094100
H	-5.60617400	0.82559400	-2.40154200
H	-5.27754100	1.30400300	-4.10445100

$R^{\cdot}$(S2)

E (SMD/B3LYP-D3/def2-SVP) = -686.098809 au
H (SMD/B3LYP-D3/def2-SVP) = -685.909851 au
G (SMD/B3LYP-D3/def2-SVP) = -685.965593 au
E (SMD/B3LYP-D3/def2-TZVP//SMD/B3LYP-D3/def2-SVP) = -686.884941 au

C	2.66624700	1.09828600	0.02212600
C	3.45995500	2.29405300	0.43578400
H	2.92153800	2.89946200	1.18133300
H	4.43422100	2.00441900	0.86603600
C	3.29652900	-0.04781100	-0.70467600
H	4.32892300	-0.18757200	-0.33419900
H	3.40558000	0.20381800	-1.77990500
C	2.51949000	-1.34203900	-0.57381600
C	1.79065100	-1.86789400	-1.65147400
C	2.49353800	-2.03008900	0.65206900
C	1.05687700	-3.05255000	-1.51223200
H	1.79958300	-1.34385000	-2.61183800
C	1.76372500	-3.21367600	0.79499100
H	3.05506200	-1.63031900	1.50190200
C	1.04134000	-3.72959600	-0.28858000
H	0.49655300	-3.44731300	-2.36442600
H	1.75909000	-3.73742500	1.75507000
C	3.78231800	3.23383000	-0.71125700
F	4.47886300	4.31038400	-0.29536700
F	4.52661400	2.63264200	-1.66651300
F	2.67068600	3.69490500	-1.31991100
H	1.57406700	1.16069500	0.05490800
H	0.47071000	-4.65579300	-0.17856400

R^{+}(S2)

E (SMD/B3LYP-D3/def2-SVP) = -685.935192 au
H (SMD/B3LYP-D3/def2-SVP) = -685.743245 au
G (SMD/B3LYP-D3/def2-SVP) = -685.794076 au
E (SMD/B3LYP-D3/def2-TZVP//SMD/B3LYP-D3/def2-SVP) = -686.719702 au

C	2.80515700	0.59546700	-1.03416100
C	2.79230200	1.47967500	0.18981500
H	1.76776600	1.62108700	0.56134100
H	3.41512600	1.08172700	1.00122500
C	3.89361900	-0.27429100	-1.36841300
H	4.71686600	-0.39815900	-0.66064100
H	4.10076100	-0.51915800	-2.41189300
C	2.53864400	-1.00083300	-0.83903200
C	1.69645700	-1.59869700	-1.83023000
C	2.58519000	-1.57007700	0.47281300
C	0.93208800	-2.70692500	-1.51679100
H	1.66876900	-1.16184500	-2.83144600
C	1.81952900	-2.68258000	0.77002000
H	3.23596300	-1.12993700	1.23007500
C	0.99459200	-3.24461400	-0.21977100
H	0.28612700	-3.16216500	-2.26964900
H	1.85644300	-3.12366000	1.76764600
C	3.34536300	2.84898700	-0.14598700
F	3.33736100	3.64791100	0.93335800
F	4.61751300	2.77945500	-0.58629900
F	2.63070600	3.46610800	-1.10742000
H	2.18459400	0.95893600	-1.85764900

H	0.38944000	-4.12137400	0.02476700

P1(S2)
E (SMD/B3LYP-D3/def2-SVP) = -1146.237297 au
H (SMD/B3LYP-D3/def2-SVP) = -1146.041755 au
G (SMD/B3LYP-D3/def2-SVP) = -1146.097418 au
E (SMD/B3LYP-D3/def2-TZVP//SMD/B3LYP-D3/def2-SVP) = -1147.182868 au

C	2.68891400	1.19671200	0.21687900
C	3.50247400	2.49084200	0.16756300
H	2.93758000	3.29763300	0.65511200
H	4.46023400	2.37534100	0.69627800
C	3.44254800	-0.05607200	-0.23793900
H	4.32980600	-0.18658700	0.40106600
H	3.80343200	0.14441200	-1.25845200
C	2.59671100	-1.30840700	-0.24947200
C	1.67635900	-1.52906400	-1.28801600
C	2.68471400	-2.25561300	0.78232300
C	0.86344100	-2.66679300	-1.29545100
H	1.59949800	-0.79953700	-2.10021000
C	1.87550900	-3.39709000	0.77682700
H	3.39275100	-2.09388700	1.59948600
C	0.96115600	-3.60577200	-0.26157500
H	0.15440200	-2.82347100	-2.11311400
H	1.95936200	-4.12596800	1.58777000
C	3.80630200	2.98821700	-1.22783100
F	4.33474800	4.22615300	-1.18591500
F	4.69224900	2.21362900	-1.89079600
F	2.69883400	3.05523000	-1.99591000
Cl	2.11971700	0.98448900	1.95019900
H	1.76591100	1.31618200	-0.36679300
H	0.32876500	-4.49771000	-0.26703200

P2(S1)
E (SMD/B3LYP-D3/def2-SVP) = -1863.16009699 au
H (SMD/B3LYP-D3/def2-SVP) = -1862.921626 au
G (SMD/B3LYP-D3/def2-SVP) = -1862.992416 au
E (SMD/B3LYP-D3/def2-TZVP//SMD/B3LYP-D3/def2-SVP) = -1864.6667199 au

C	2.65245500	1.16456900	0.00892000
C	3.31676500	2.28216000	-0.78196500
H	4.31003900	2.51728600	-0.37877600
H	3.45694500	1.88980800	-1.80102100
C	3.53439800	-0.10017200	-0.00731200
C	2.53249000	3.56831000	-0.92990800
F	3.05190600	4.31157900	-1.92433400
F	1.23862500	3.34258600	-1.23270200
F	2.55726500	4.32876900	0.17790800
N	2.89554000	-1.27571300	-0.24591100
C	1.49038900	-1.38116600	-0.53270500
C	0.58055100	-1.61788800	0.50514600
C	1.04555200	-1.27631500	-1.85702800
C	-0.78244100	-1.73122300	0.21502400
H	0.94527500	-1.69004900	1.53063300
C	-0.31848400	-1.39028200	-2.14026200
H	1.77217900	-1.09542600	-2.65258900
C	-1.23295000	-1.61675900	-1.10500600
H	-1.49501900	-1.90630700	1.02502700
H	-0.66738500	-1.30037300	-3.17208500
H	-2.29952900	-1.70358600	-1.32780400
O	4.73498000	0.01329900	0.19236200
C	3.65115900	-2.52738000	-0.23688300
H	3.58204300	-3.01806700	-1.22059000
H	4.70122900	-2.31231900	-0.00756500
H	3.23967400	-3.20717600	0.52584200
H	1.63030000	0.98581100	-0.35257000
S	2.27743400	1.54170100	1.78454700
O	2.19313700	0.28762200	2.52759100
O	1.19504900	2.51475600	1.86009700
Cl	4.06136800	2.51427400	2.45599200

E(S1)
E (SMD/B3LYP-D3/def2-SVP) = -1863.30490869 au
H (SMD/B3LYP-D3/def2-SVP) = -1863.070931 au

G (SMD/B3LYP-D3/def2-SVP) = -1863.147864 au
E (SMD/B3LYP-D3/def2-TZVP//SMD/B3LYP-D3/def2-SVP) = -1864.81243179 au

C	2.69055500	1.15912700	-0.00559700
C	3.43206500	2.38394800	-0.51863600
H	4.18227700	2.72171000	0.21130000
H	3.94844700	2.13647500	-1.45795400
C	3.53927700	-0.09237800	-0.10920700
C	2.53393500	3.55316600	-0.82562500
F	3.24315300	4.57948800	-1.34030400
F	1.58549500	3.23811600	-1.73973500
F	1.88375500	4.02850200	0.25117500
N	2.86978300	-1.27039600	-0.30922300
C	1.45318000	-1.36004300	-0.52454800
C	0.56918700	-1.41932200	0.56031100
C	0.96225800	-1.42429500	-1.83663700
C	-0.80570700	-1.52927400	0.32798400
H	0.96889500	-1.34339200	1.57216200
C	-0.41219700	-1.53695500	-2.06284100
H	1.66492300	-1.37557400	-2.67211000
C	-1.29848100	-1.58943500	-0.97996300
H	-1.49522100	-1.56259200	1.17573100
H	-0.79237000	-1.57952300	-3.08689200
H	-2.37394100	-1.67412500	-1.15713200
O	4.75978200	-0.02481100	-0.00527900
C	3.60548900	-2.52954300	-0.34038200
H	3.48203200	-3.02806900	-1.31602500
H	4.66913700	-2.32397300	-0.17131600
H	3.23412000	-3.20733700	0.44585500
H	1.70087700	1.05188700	-0.46264000
S	2.22444800	1.38695000	1.88293100
O	2.56539800	0.08835100	2.55299400
O	0.76844900	1.73898300	1.85572700
Cl	4.12320400	3.28957700	2.73737900

TS_{S1}
E (SMD/B3LYP-D3/def2-SVP) = -1863.29750631 au
H (SMD/B3LYP-D3/def2-SVP) = -1863.063211 au
G (SMD/B3LYP-D3/def2-SVP) = -1863.139004 au
E (SMD/B3LYP-D3/def2-TZVP//SMD/B3LYP-D3/def2-SVP) = -1864.80572836 au

C	2.72988500	1.16066500	-0.28935100
C	3.47830200	2.44806300	-0.47616900
H	4.05197300	2.71985700	0.42670000
H	4.20235900	2.36748400	-1.30387400
C	3.53752900	-0.07062000	-0.26538800
C	2.57259300	3.60920500	-0.78749800
F	3.27122800	4.75413300	-0.92392800
F	1.88848300	3.42820500	-1.94016000
F	1.65175900	3.82180700	0.17375400
N	2.85577900	-1.26867600	-0.32053700
C	1.43787200	-1.36046700	-0.50556200
C	0.58519800	-1.46166300	0.60217500
C	0.90652500	-1.37869500	-1.80327000
C	-0.79529000	-1.57181600	0.40839100
H	1.01272500	-1.42323200	1.60511300
C	-0.47446400	-1.48628700	-1.99201100
H	1.58453600	-1.29950900	-2.65657500
C	-1.32724900	-1.58388000	-0.88595600
H	-1.45891300	-1.63929400	1.27456600
H	-0.88534300	-1.49333100	-3.00506100
H	-2.40729300	-1.66681800	-1.03369100
O	4.76650500	-0.01686600	-0.16214200
C	3.57467400	-2.52555800	-0.17017000
H	3.44925700	-3.15856000	-1.06510400
H	4.64017400	-2.30780100	-0.02886600
H	3.19834100	-3.08431700	0.70341800
H	1.69544300	1.11470200	-0.63510700
S	2.18724700	1.30602500	2.06087100
O	2.64558500	0.04199000	2.69933700
O	0.73361300	1.56891100	2.20470700
Cl	3.55733600	3.24259200	3.23807500

R$^{\cdot}$(S1)
E (SMD/B3LYP-D3/def2-SVP) = -854.681635945 au

H (SMD/B3LYP-D3/def2-SVP) = -854.462407 au
G (SMD/B3LYP-D3/def2-SVP) = -854.523459 au
E (SMD/B3LYP-D3/def2-TZVP//SMD/B3LYP-D3/def2-SVP) = -855.659530635 au

C	2.98530700	1.08507900	-0.30507600
C	3.74757300	2.35908600	-0.23751400
H	3.93796700	2.64503500	0.81528200
H	4.72643700	2.26181300	-0.72799700
C	3.71387100	-0.17052300	-0.47540300
C	3.00967300	3.51782700	-0.86320400
F	3.71497900	4.66005100	-0.76609100
F	2.76269200	3.31330700	-2.17333500
F	1.81558300	3.74054400	-0.27327800
H	1.90757400	1.12193400	-0.15056900
N	2.97372600	-1.34533000	-0.50588000
C	3.64209900	-2.60464700	-0.52845500
C	4.67338600	-2.86618600	-1.44821300
C	3.25061600	-3.61684800	0.36533400
C	5.29900300	-4.11470500	-1.46404500
H	4.98103100	-2.08970200	-2.14718400
C	3.87639400	-4.86675700	0.33703800
H	2.45884100	-3.42483200	1.09241600
C	4.90547500	-5.12195200	-0.57470600
H	6.09731200	-4.30391700	-2.18694900
H	3.55921600	-5.64180600	1.03996200
H	5.39682300	-6.09814100	-0.59390300
O	4.94751200	-0.16593900	-0.56246800
C	1.51414000	-1.34226300	-0.36590400
H	1.19017400	-1.11180500	0.66223000
H	1.06098000	-0.61500100	-1.05390300
H	1.12828200	-2.33147200	-0.63940200

F(S1)
E (SMD/B3LYP-D3/def2-SVP) = -1863.30490869 au
H (SMD/B3LYP-D3/def2-SVP) = -1863.070931 au
G (SMD/B3LYP-D3/def2-SVP) = -1863.147864 au
E (SMD/B3LYP-D3/def2-TZVP//SMD/B3LYP-D3/def2-SVP) = -1864.81243179 au

C	2.87335300	1.23279100	-0.37370100
C	3.57667600	2.49390100	-0.03037400
H	3.24107800	2.82815300	0.96860300
H	4.66103300	2.33756600	0.03509200
C	3.59354600	-0.03400600	-0.34246700
C	3.30059000	3.61577400	-0.99529300
F	3.92099700	4.75339800	-0.62295900
F	3.71948800	3.33321300	-2.24820900
F	1.98212400	3.90248500	-1.08731300
N	2.86969000	-1.21303600	-0.42799000
C	1.45007800	-1.30002100	-0.36668700
C	0.73990500	-0.74145900	0.70943100
C	0.75068500	-1.98439400	-1.37637300
C	-0.65403200	-0.84694800	0.75325100
H	1.27801200	-0.22405800	1.50893700
C	-0.63998300	-2.10070200	-1.31394400
H	1.30241200	-2.41765700	-2.21404400
C	-1.34988200	-1.52698400	-0.25202600
H	-1.19655000	-0.40298700	1.59263200
H	-1.17226600	-2.63385000	-2.10631600
H	-2.43864800	-1.61370300	-0.20739200
O	4.82980100	-0.07323400	-0.29457400
C	3.65318800	-2.43896000	-0.56904700
H	4.19361700	-2.46538300	-1.52981100
H	4.40190600	-2.50101000	0.23602300
H	2.98661000	-3.30710800	-0.50571000
H	1.81578100	1.27517700	-0.63489700
Cl	2.58369000	1.36044400	3.13541400
S	5.13769800	0.76564400	2.85062800
O	5.69489800	2.07141100	2.41632800
O	5.50556700	0.39151800	4.23713800

P'1_1
E (SMD/B3LYP-D3/def2-SVP) = -1863.18797032 au
H (SMD/B3LYP-D3/def2-SVP) = -1862.950054 au
G (SMD/B3LYP-D3/def2-SVP) = -1863.025793 au
E (SMD/B3LYP-D3/def2-TZVP//SMD/B3LYP-D3/def2-SVP) = -1864.68281273 au

C	2.69282900	1.26045500	0.76474400
C	3.29102700	2.51198200	0.13272600
H	2.78640200	3.40512300	0.52587100
H	4.36349600	2.58868200	0.35146500
C	3.53658200	0.00748800	0.49481000
C	3.10633400	2.52909900	-1.36676800
F	3.64692300	3.63127100	-1.91059800
F	3.68422600	1.46408100	-1.96983300
F	1.80340100	2.50584200	-1.71807000
N	2.87672800	-1.10369700	0.09586100
C	1.46434200	-1.12865800	-0.17616000
C	0.57071300	-1.48891400	0.83900400
C	0.99955600	-0.81570300	-1.45951300
C	-0.80041900	-1.52519400	0.56871000
H	0.95584500	-1.72184700	1.83423300
C	-0.37272900	-0.85437700	-1.72268800
H	1.71445300	-0.52616500	-2.23233300
C	-1.27241100	-1.20895700	-0.71057400
H	-1.50173500	-1.79819000	1.36123100
H	-0.74007100	-0.60207000	-2.72064500
H	-2.34508900	-1.23630900	-0.91879700
O	4.76093100	0.03819700	0.65031700
C	3.60699800	-2.34607600	-0.15592600
H	3.61001900	-2.57348600	-1.23428700
H	4.63906200	-2.23744200	0.19630500
H	3.11688300	-3.17397000	0.37799800
H	1.65543900	1.13645200	0.44266400
Cl	2.61239700	1.45242600	2.57353500
S	6.33173600	1.20397800	2.31919700
O	5.92650700	2.61011200	2.16488400
O	5.89068500	0.53184000	3.54698400

P1(S1)
E (SMD/B3LYP-D3/def2-SVP) = -1314.8078304 au
H (SMD/B3LYP-D3/def2-SVP) = -1314.583045 au
G (SMD/B3LYP-D3/def2-SVP) = -1314.646377 au
E (SMD/B3LYP-D3/def2-TZVP//SMD/B3LYP-D3/def2-SVP) = -1315.94491767 au

C	2.90598800	1.19908400	-0.24019800
C	3.70480500	2.27373100	-0.95552700
H	4.65793300	2.46289300	-0.44456800
H	3.93446600	1.90214600	-1.96615400
C	3.69198600	-0.12254400	-0.21335000
C	2.98796300	3.58958300	-1.14484200
F	3.66548200	4.37316200	-2.00766100
F	1.74796000	3.42205200	-1.65415000
F	2.85120600	4.29422600	-0.00733800
N	2.94971700	-1.26732600	-0.20769500
C	1.51478700	-1.28946000	-0.26892000
C	0.76319800	-1.26910600	0.91255100
C	0.87338700	-1.37180100	-1.51218700
C	-0.63266300	-1.31764700	0.84750600
H	1.27835100	-1.20359100	1.87253300
C	-0.52238800	-1.41803000	-1.57225300
H	1.47577200	-1.38944700	-2.42356100
C	-1.27630700	-1.39044900	-0.39298400
H	-1.21895300	-1.29591000	1.76973400
H	-1.02218100	-1.47489500	-2.54262900
H	-2.36778500	-1.42595500	-0.44122500
O	4.91543300	-0.10533400	-0.18333100
C	3.61211800	-2.56707300	-0.12364700
H	4.69739500	-2.41441300	-0.09591200
H	3.29326500	-3.09895100	0.78738200
H	3.35106500	-3.18387000	-0.99852200
H	1.91720400	1.08494500	-0.69605000
Cl	2.58240200	1.64187700	1.51011400

R$^+$(S1)
E (SMD/B3LYP-D3/def2-SVP) = -854.494819394 au
H (SMD/B3LYP-D3/def2-SVP) = -854.272991 au
G (SMD/B3LYP-D3/def2-SVP) = -854.331972 au
E (SMD/B3LYP-D3/def2-TZVP//SMD/B3LYP-D3/def2-SVP) = -855.472102073 au

C	2.97938400	1.10086800	-1.14303900
C	3.20643800	2.14651300	-0.09959600

H	2.22394600	2.40360000	0.32908800
H	3.85286600	1.78065100	0.71033300
C	3.11682400	-0.29151800	-0.86410800
C	3.80800000	3.41248700	-0.67317700
F	3.94992300	4.34557600	0.27847500
F	5.02139500	3.18583900	-1.20881000
F	3.03742300	3.93547700	-1.64522900
H	2.46439900	1.38275200	-2.06916000
N	2.68763900	-1.40923300	-0.39724700
C	3.49549200	-2.60778200	-0.50806200
C	4.06111500	-2.94013400	-1.74391900
C	3.68283500	-3.41163900	0.62088000
C	4.84173900	-4.09444500	-1.84245300
H	3.88488900	-2.31467400	-2.62121000
C	4.46164400	-4.56549000	0.50467800
H	3.23712800	-3.14005800	1.57895500
C	5.04267100	-4.90677700	-0.72171100
H	5.28579500	-4.36126400	-2.80426700
H	4.61695700	-5.19782500	1.38189400
H	5.65109100	-5.81058600	-0.80459200
O	4.18463400	0.14220900	-1.43953000
C	1.35999100	-1.46697900	0.24705900
H	1.48516500	-1.67050500	1.31840800
H	0.85547100	-0.50281200	0.11060700
H	0.78181200	-2.27140000	-0.22548600

P2_3
E (SMD/B3LYP-D3/def2-SVP) = -1828.473892 au
H (SMD/B3LYP-D3/def2-SVP) = -1828.187998 au
G (SMD/B3LYP-D3/def2-SVP) = -1828.263042 au
E (SMD/B3LYP-D3/def2-TZVP//SMD/B3LYP-D3/def2-SVP) = -1829.935214 au

C	2.95604500	1.23977800	0.37677500
C	3.80046900	2.47739600	0.06671600
H	3.51829500	3.32963700	0.70019200
H	4.86936900	2.27503500	0.22116400
C	3.61084800	-0.09246900	-0.05453000
H	4.49906900	-0.27532700	0.57046200
H	3.98948500	0.10716900	-1.06867000
C	2.70099300	-1.29448400	-0.08342100
C	1.64312000	-1.37223000	-1.00312800
C	2.86746400	-2.37255400	0.79619800
C	0.78389100	-2.46762100	-1.04726700
H	1.48438200	-0.54944000	-1.70816300
C	2.02532000	-3.48349900	0.76787300
H	3.67620100	-2.34334900	1.53267400
C	0.94985700	-3.56497700	-0.15733600
H	-0.01910300	-2.46987900	-1.78374100
H	2.20663500	-4.29055800	1.47695400
C	3.61427500	2.96388000	-1.35446200
F	4.31847800	4.08860900	-1.56286100
F	4.01887200	2.06833600	-2.27968700
F	2.32357100	3.24148400	-1.62353700
S	2.66326000	1.15510100	2.19468400
O	1.76299200	0.05852600	2.52747200
O	3.92851000	1.32493100	2.90482000
Cl	1.51236600	2.97943100	2.51384800
H	1.93827900	1.33741600	-0.02914600
N	0.10899200	-4.65485600	-0.19346600
C	0.30217800	-5.75324800	0.73406200
H	-0.45591100	-6.52468400	0.54975800
H	1.29635300	-6.22330300	0.61912800
H	0.20734300	-5.43103900	1.78789200
C	-0.98971700	-4.69586100	-1.13979100
H	-1.70080700	-3.86220700	-0.98947000
H	-0.63850300	-4.64764800	-2.18711600
H	-1.54333700	-5.63502400	-1.01581100

E(S3)
E (SMD/B3LYP-D3/def2-SVP) = -1828.611983 au
H (SMD/B3LYP-D3/def2-SVP) = -1828.327745 au
G (SMD/B3LYP-D3/def2-SVP) = -1828.405533 au
E (SMD/B3LYP-D3/def2-TZVP//SMD/B3LYP-D3/def2-SVP) = -1830.076056 au

C	2.93165600	1.22530300	0.33872200

C	3.80345900	2.46389900	0.14013900
H	3.48868100	3.24421500	0.85327900
H	4.86603000	2.23551000	0.31045400
C	3.50359200	-0.04639100	-0.30874000
H	4.47848700	-0.24681700	0.16372900
H	3.70784700	0.20657500	-1.36059200
C	2.63345600	-1.27855100	-0.26040000
C	1.64456500	-1.50796700	-1.22852200
C	2.76744600	-2.23931400	0.75370600
C	0.82648400	-2.63803700	-1.20018100
H	1.50565100	-0.77965600	-2.03452000
C	1.96868600	-3.38042800	0.80013900
H	3.50563000	-2.07460000	1.54311000
C	0.97187000	-3.61992100	-0.18390700
H	0.07629600	-2.75721200	-1.98152800
H	2.12132300	-4.08881800	1.61406600
C	3.68407900	3.09196300	-1.22387200
F	4.37155400	4.25169100	-1.28125600
F	4.16214600	2.31746500	-2.22579700
F	2.40373100	3.37962700	-1.54173800
S	2.73545400	0.96344200	2.20260500
O	1.54217800	0.07192100	2.37130900
O	4.06713900	0.40616200	2.63818300
Cl	1.90031000	3.68017500	2.71267100
H	1.89821900	1.42599300	0.02062000
N	0.18400900	-4.75324600	-0.15463200
C	0.28851600	-5.67893600	0.95661600
H	-0.40600200	-6.51444000	0.80162100
H	1.30529900	-6.10179800	1.04529100
H	0.04069500	-5.20579300	1.92661400
C	-0.88317400	-4.91849100	-1.12212100
H	-1.64821600	-4.12095500	-1.05037300
H	-0.50118000	-4.91777100	-2.15868300
H	-1.38238700	-5.88131700	-0.95411700

TS_{S3}
E (SMD/B3LYP-D3/def2-SVP) = -1828.594828 au
H (SMD/B3LYP-D3/def2-SVP) = -1828.313618 au
G (SMD/B3LYP-D3/def2-SVP) = -1828.392850 au
E (SMD/B3LYP-D3/def2-TZVP//SMD/B3LYP-D3/def2-SVP) = -1830.061107 au

C	2.91221100	1.33283300	-0.22256900
C	3.69470400	2.61039600	-0.29645000
H	3.25766400	3.38432400	0.35427900
H	4.73827800	2.45539300	0.02136700
C	3.57156400	0.03593500	-0.59272100
H	4.41654500	-0.13284600	0.10041700
H	4.03154800	0.15711500	-1.59391200
C	2.64706600	-1.16066500	-0.60447100
C	1.92564500	-1.50680100	-1.75749000
C	2.43754900	-1.94793600	0.53892500
C	1.04053200	-2.58543900	-1.78439700
H	2.05686500	-0.91380400	-2.66879600
C	1.55997900	-3.03247400	0.53503300
H	2.96112000	-1.69265900	1.46299300
C	0.83565600	-3.39133600	-0.63289400
H	0.51151600	-2.80171800	-2.71230500
H	1.43808300	-3.60245800	1.45589500
C	3.74974500	3.21375500	-1.68386000
F	4.43282300	4.37638600	-1.69256200
F	4.35549900	2.40079400	-2.57840200
F	2.52014000	3.47751800	-2.17273400
S	2.81835500	1.14525200	2.34047100
O	1.50950000	0.50146500	2.62893500
O	3.99201100	0.25219100	2.57292300
Cl	3.06387400	2.91114000	4.27100100
H	1.82694300	1.41388200	-0.34469900
N	-0.02519400	-4.47131100	-0.64834900
C	-0.84108900	-4.72843500	-1.81912100
H	-0.22450000	-4.91258800	-2.71689400
H	-1.45213600	-5.62400300	-1.64841700
H	-1.52475200	-3.88893800	-2.05294900
C	-0.28651900	-5.20489100	0.57491600
H	-0.97119000	-6.03601600	0.36257600
H	0.63760800	-5.63566700	0.99937200

H	-0.74871800	-4.57382700	1.35915900

$R^{\cdot}$(S3)

E (SMD/B3LYP-D3/def2-SVP) = -819.980639 au
H (SMD/B3LYP-D3/def2-SVP) = -819.714964 au
G (SMD/B3LYP-D3/def2-SVP) = -819.781095 au
E (SMD/B3LYP-D3/def2-TZVP//SMD/B3LYP-D3/def2-SVP) = -820.912029 au

C	2.72093600	1.13411900	0.05497400
C	3.58190400	2.29511000	0.43268300
H	3.12763300	2.89354300	1.23783400
H	4.58023400	1.96643500	0.76915800
C	3.26010100	-0.02251100	-0.72545700
H	4.31184700	-0.19520600	-0.42584700
H	3.31382600	0.24464700	-1.80174200
C	2.45351800	-1.29595900	-0.57429700
C	1.77419400	-1.88147400	-1.65178600
C	2.34084700	-1.93709600	0.67086300
C	1.02110000	-3.04904400	-1.50941500
H	1.83133500	-1.41247300	-2.63941000
C	1.59653800	-3.10340400	0.84000500
H	2.85178400	-1.50973700	1.53984000
C	0.91130100	-3.70146300	-0.25316500
H	0.51679300	-3.45122900	-2.38769600
H	1.55061200	-3.55209400	1.83206600
C	3.83280400	3.25930900	-0.71158800
F	4.59657900	4.30278600	-0.32948200
F	4.47185500	2.66650300	-1.74502600
F	2.68744800	3.77112600	-1.20714400
H	1.63602000	1.23634800	0.15860400
N	0.17614700	-4.85821700	-0.09882000
C	0.04354500	-5.46538400	1.21127400
H	-0.56735700	-6.37351800	1.13257500
H	1.02285700	-5.75611900	1.63344400
H	-0.44440200	-4.79083600	1.94061500
C	-0.55631100	-5.40756000	-1.22305800
H	-1.31939600	-4.70598800	-1.61128000
H	0.11164200	-5.66771100	-2.06448300
H	-1.07093100	-6.32516800	-0.91104900

R^{+}(S3)

E (SMD/B3LYP-D3/def2-SVP) = -819.851143 au
H (SMD/B3LYP-D3/def2-SVP) = -819.581351 au
G (SMD/B3LYP-D3/def2-SVP) = -819.641322 au
E (SMD/B3LYP-D3/def2-TZVP//SMD/B3LYP-D3/def2-SVP) = -820.780707 au

C	2.73423800	0.58509500	-1.01545800
C	2.76186100	1.49493900	0.19152900
H	1.74486300	1.69749500	0.55845100
H	3.35256700	1.07726300	1.01748000
C	3.85475800	-0.28955500	-1.36554700
H	4.69667500	-0.36027100	-0.67202900
H	4.08511200	-0.47734100	-2.41654800
C	2.53845500	-0.96301300	-0.84459500
C	1.68145200	-1.61188000	-1.82551800
C	2.55928800	-1.56569300	0.47933300
C	0.93756200	-2.70929000	-1.53031100
H	1.64544400	-1.18789200	-2.83262300
C	1.82355900	-2.66655500	0.78462000
H	3.19970200	-1.12525100	1.24604600
C	0.97136300	-3.29086500	-0.20546400
H	0.31466500	-3.15132800	-2.30515300
H	1.88514600	-3.07873700	1.78968900
C	3.38763800	2.82759400	-0.14871800
F	3.39827400	3.64918400	0.91701300
F	4.66570700	2.69463500	-0.56053100
F	2.72530000	3.46682400	-1.13515100
H	2.14052200	0.97668700	-1.84633600
N	0.24525300	-4.36644200	0.09138300
C	0.29062500	-4.96316000	1.43107700
H	-0.38052000	-5.82756700	1.45842400
H	1.30977000	-5.30072500	1.67365000
H	-0.03730600	-4.23947900	2.19234700
C	-0.61866800	-4.99484600	-0.91432500
H	-1.37382400	-4.28286900	-1.28012000

H	-0.02542200	-5.35652000	-1.76813100
H	-1.13292100	-5.84701200	-0.45892500

P1_3
E (SMD/B3LYP-D3/def2-SVP) = -1280.119422 au
H (SMD/B3LYP-D3/def2-SVP) = -1279.847143 au
G (SMD/B3LYP-D3/def2-SVP) = -1279.913954 au
E (SMD/B3LYP-D3/def2-TZVP//SMD/B3LYP-D3/def2-SVP) = -1281.210316 au

C	2.69318600	1.18810700	0.22923200
C	3.47934600	2.49994200	0.19529400
H	2.90550300	3.28564500	0.70633600
H	4.44654100	2.39372800	0.70876700
C	3.46507300	-0.04267800	-0.25631000
H	4.36164400	-0.16309600	0.37282600
H	3.81667800	0.19012700	-1.27360000
C	2.65285900	-1.31508400	-0.28800900
C	1.76400200	-1.57926300	-1.34194800
C	2.72053300	-2.26557700	0.74111000
C	0.97718300	-2.73010100	-1.37701800
H	1.68428000	-0.86360800	-2.16701400
C	1.94690700	-3.42623400	0.72863800
H	3.39852000	-2.09518800	1.58244900
C	1.04209700	-3.69346100	-0.33392600
H	0.31078300	-2.87901800	-2.22618500
H	2.05095100	-4.12855500	1.55526700
C	3.75459400	3.03199300	-1.19313800
F	4.25575100	4.28090600	-1.13254300
F	4.64976900	2.29197400	-1.88280800
F	2.63656900	3.08981100	-1.94671500
Cl	2.14659400	0.93692300	1.96563900
H	1.76111300	1.29889400	-0.34173400
N	0.26790800	-4.83413300	-0.35473100
C	0.39706100	-5.81820400	0.70251000
H	0.16268100	-5.39121100	1.69461000
H	-0.30378000	-6.64280100	0.52140000
H	1.41659600	-6.24538900	0.75804100
C	-0.59430800	-5.10665000	-1.48855300
H	-1.35136100	-4.31424300	-1.63213000
H	-0.02772500	-5.19908400	-2.43477900
H	-1.12842800	-6.05083400	-1.32285500

P2_4
E (SMD/B3LYP-D3/def2-SVP) = -1848.32818 au
H (SMD/B3LYP-D3/def2-SVP) = -1848.054782 au
G (SMD/B3LYP-D3/def2-SVP) = -1848.125093 au
E (SMD/B3LYP-D3/def2-TZVP//SMD/B3LYP-D3/def2-SVP) = -1849.812992 au

C	2.66653900	1.32428200	0.21220200
C	3.66758200	2.49451400	0.21004600
H	3.24701300	3.35344000	0.74935300
H	4.60016100	2.20238900	0.71428600
C	3.27476300	0.05232800	-0.44814200
H	4.29437000	-0.08315200	-0.06010100
H	3.37548200	0.33104600	-1.50768800
C	2.50311400	-1.24306800	-0.35640500
C	1.44688300	-1.52390000	-1.24288700
C	2.82686900	-2.21033300	0.60372800
C	0.73380700	-2.71574000	-1.15973400
H	1.18331000	-0.79611800	-2.01478100
C	2.12454100	-3.41655300	0.69994800
H	3.64301700	-2.01912400	1.30566100
C	1.06344900	-3.67510100	-0.18349400
H	-0.08528700	-2.93321700	-1.84918600
H	2.41152300	-4.14146100	1.46184600
O	0.31687800	-4.80431900	-0.17855500
C	0.58985600	-5.81099600	0.78300900
H	-0.13452900	-6.61754700	0.60402700
H	1.61131000	-6.21662600	0.67298300
H	0.46220900	-5.43733300	1.81453100
C	1.27326400	1.68398600	-0.28367400
H	1.32431500	1.86733000	-1.36581800
H	0.56547400	0.86410600	-0.10723600
H	0.89947500	2.59519200	0.20477600
C	4.07992800	3.04464500	-1.14027900

F	4.88935900	4.10511700	-0.95967500
F	4.76183100	2.16209700	-1.89711000
F	3.03574400	3.46964500	-1.87819100
S	2.47147000	0.85244900	2.04890200
O	1.30389700	0.00021800	2.24822900
O	3.77184300	0.47339400	2.60066500
Cl	1.93829100	2.75522900	2.98951300

E(S4)
E (SMD/B3LYP-D3/def2-SVP) = -1848.468572 au
H (SMD/B3LYP-D3/def2-SVP) = -1848.197004 au
G (SMD/B3LYP-D3/def2-SVP) = -1848.270807 au
E (SMD/B3LYP-D3/def2-TZVP//SMD/B3LYP-D3/def2-SVP) = -1849.955646 au

C	2.73661600	1.25340200	0.24389000
C	3.70435200	2.44779900	0.19263100
H	3.34454400	3.22802500	0.88454100
H	4.70484900	2.13545300	0.52617100
C	3.34459900	0.00062400	-0.43044100
H	4.34690300	-0.15432900	-0.00410500
H	3.48632700	0.26986800	-1.48850700
C	2.55576100	-1.28617800	-0.36090200
C	1.58583100	-1.59580600	-1.33159700
C	2.76889600	-2.21651800	0.66517700
C	0.85521900	-2.78059800	-1.27595400
H	1.40088300	-0.89311200	-2.14906600
C	2.05149600	-3.41601100	0.73461100
H	3.50084300	-1.98744200	1.44323800
C	1.08169400	-3.70491900	-0.23983500
H	0.10280400	-3.01774500	-2.03205100
H	2.25144300	-4.11108600	1.55060500
O	0.32947300	-4.83365600	-0.26729100
C	0.50278800	-5.80364000	0.75130800
H	-0.20294500	-6.61704300	0.53187400
H	1.52858900	-6.21389700	0.75864700
H	0.27555900	-5.39431800	1.75202200
C	1.31824200	1.58219000	-0.19791200
H	1.29291200	1.77401800	-1.28152000
H	0.64486300	0.74281500	0.02149900
H	0.95413100	2.47474100	0.33257200
C	3.89571100	3.13765300	-1.13370800
F	4.80678200	4.12791800	-1.01398800
F	4.34595500	2.32547600	-2.11766900
F	2.76610500	3.71283800	-1.59999000
S	2.62674200	0.89027800	2.15120100
O	1.45921300	-0.03013500	2.34552300
O	3.98412300	0.33571700	2.50198000
Cl	1.78644800	3.60443800	2.82779400

TS_{S3}
E (SMD/B3LYP-D3/def2-SVP) = -1848.454862 au
H (SMD/B3LYP-D3/def2-SVP) = -1848.185878 au
G (SMD/B3LYP-D3/def2-SVP) = -1848.26108 au
E (SMD/B3LYP-D3/def2-TZVP//SMD/B3LYP-D3/def2-SVP) = -1849.946999 au

C	2.74712500	1.30359800	-0.19838200
C	3.54205800	2.58686200	-0.23481200
H	3.10470700	3.33814100	0.44164000
H	4.58101900	2.41442900	0.08744000
C	3.48373700	0.06033500	-0.65174600
H	4.39150000	-0.04125000	-0.03254300
H	3.84171100	0.24646200	-1.68262700
C	2.69226500	-1.22867800	-0.63514100
C	1.95822800	-1.63546200	-1.76446400
C	2.64372100	-2.04601500	0.50235700
C	1.20524500	-2.80722500	-1.75889600
H	1.97793300	-1.01925200	-2.66842200
C	1.89436400	-3.22920600	0.52577800
H	3.18985900	-1.74428900	1.39818300
C	1.16534300	-3.61813400	-0.60986500
H	0.63806400	-3.12009400	-2.63898200
H	1.88577200	-3.83540600	1.43214000
O	0.40907400	-4.74156700	-0.69357300
C	0.32360600	-5.60155500	0.42975800
H	-0.33394600	-6.43249900	0.13804100

H	1.31085200	-6.00987200	0.71142600
H	-0.11403200	-5.09112400	1.30646300
C	1.26280000	1.39084000	-0.37000500
H	0.98606200	1.38448000	-1.44239100
H	0.76002400	0.53356400	0.10240600
H	0.86190500	2.31653300	0.07132000
C	3.62367900	3.25184800	-1.59266900
F	4.33885600	4.39404500	-1.53555300
F	4.21563300	2.47078500	-2.52378900
F	2.40770800	3.57831900	-2.08088100
S	2.84512400	1.04035600	2.29909700
O	1.60752600	0.27483200	2.62030400
O	4.10217200	0.24632100	2.45613300
Cl	3.02598400	2.80400300	4.28102200

R⁺(S4)
E (SMD/B3LYP-D3/def2-SVP) = -839.6861856 au
H (SMD/B3LYP-D3/def2-SVP) = -839.429745 au
G (SMD/B3LYP-D3/def2-SVP) = -839.488027 au
E (SMD/B3LYP-D3/def2-TZVP//SMD/B3LYP-D3/def2-SVP) = -840.6421168 au

C	2.97649900	0.87067500	0.25400400
C	3.16081800	1.61847000	-1.05463600
H	3.51355400	2.63126600	-0.80078100
H	3.92988200	1.16421500	-1.69013600
C	4.08031600	0.07273700	0.75505500
H	4.18640000	-0.04697400	1.83599300
H	5.01108500	0.03664900	0.18349900
C	2.94699300	-0.81460500	0.13635700
C	3.15595400	-1.40073400	-1.16466500
C	2.01749700	-1.48015800	1.01014800
C	2.46736600	-2.51242700	-1.56401600
H	3.88449200	-0.95412800	-1.84221000
C	1.31551100	-2.59074700	0.61492600
H	1.86840000	-1.09690400	2.01996200
C	1.51989400	-3.11723900	-0.69093500
H	2.62160600	-2.95580400	-2.54907200
H	0.61656300	-3.06140700	1.30488200
O	0.88404900	-4.16265000	-1.17190800
C	-0.10546100	-4.85844600	-0.39490700
H	-0.48139000	-5.65926900	-1.04271600
H	0.34719800	-5.29222400	0.50987500
H	-0.92968300	-4.18167200	-0.12181500
C	1.97346900	1.47810400	1.21253300
H	0.94345100	1.38407900	0.84335900
H	2.03893900	1.04057200	2.21581000
H	2.20418100	2.55232200	1.29916400
C	1.92502100	1.79512200	-1.91047300
F	2.24620000	2.38461100	-3.07659100
F	1.33005500	0.62245800	-2.20826400
F	0.98798700	2.56838400	-1.32552500

P1_4
E (SMD/B3LYP-D3/def2-SVP) = -1299.971850 au
H (SMD/B3LYP-D3/def2-SVP) = -1299.712326 au
G (SMD/B3LYP-D3/def2-SVP) = -1299.775229 au
E (SMD/B3LYP-D3/def2-TZVP//SMD/B3LYP-D3/def2-SVP) = -1301.089742 au

C	2.58640900	1.24690100	0.18897400
C	3.52191500	2.47277100	0.19520800
H	3.04217800	3.29361700	0.74697600
H	4.45857200	2.22377700	0.71440500
C	3.28551600	-0.00881200	-0.37735300
H	4.23888900	-0.13616600	0.15787400
H	3.53581100	0.22607600	-1.42264300
C	2.49005500	-1.29301000	-0.34140600
C	1.64198000	-1.64888300	-1.40688100
C	2.56263800	-2.16830000	0.75038900
C	0.89163400	-2.82153700	-1.37721000
H	1.56998100	-0.99205400	-2.27823400
C	1.81806200	-3.35298500	0.79904300
H	3.21429400	-1.92053900	1.59173600
C	0.96975300	-3.68629000	-0.27002000
H	0.23579000	-3.09437700	-2.20744500
H	1.90838300	-4.00510800	1.66809600

O	0.20552600	-4.80426000	-0.32688500
C	0.23603300	-5.71889700	0.75610700
H	-0.45417200	-6.53357000	0.49620000
H	1.24541300	-6.14108500	0.90886300
H	-0.10173700	-5.25110900	1.69817500
C	1.23205800	1.52539200	-0.45118400
H	1.35115900	1.64101000	-1.53878800
H	0.53966500	0.69296800	-0.26815800
H	0.79044800	2.44788300	-0.04714600
C	3.92753200	3.06551900	-1.13765900
F	4.73095500	4.12982900	-0.93455900
F	4.61582300	2.21025000	-1.92369800
F	2.88128300	3.50535000	-1.86669000
Cl	2.26209700	0.94712200	2.00526400

X1

E (SMD/B3LYP-D3/def2-SVP) = -3362.3164 au
H (SMD/B3LYP-D3/def2-SVP) = -3361.8864 au
G (SMD/B3LYP-D3/def2-SVP) = -3361.9722 au
E (SMD/B3LYP-D3/def2-TZVP//SMD/B3LYP-D3/def2-SVP) = -3364.080 au

Cu	0.30471700	0.10834600	0.12638900
N	1.57943400	1.06631700	-1.12475700
C	2.60194100	0.23705500	-1.46674300
C	2.66119600	-1.05189200	-0.83273000
N	1.73873600	-1.33514300	0.12911200
C	1.78971200	-2.49136300	0.80560500
C	0.91264000	-2.70605000	1.97055500
C	0.69906700	-1.66937400	2.90274800
C	-0.07538200	-1.87697400	4.03339200
C	-0.68177100	-3.12853800	4.26046300
C	-0.47833700	-4.17226600	3.33808500
C	0.32382700	-3.95750600	2.21701400
O	-1.42871500	-3.23340800	5.37689200
C	-2.07414200	-4.46423000	5.67622000
C	2.75350000	-3.47455300	0.44443800
C	3.68088100	-3.21744800	-0.54021000
C	3.68209400	-1.96123200	-1.19648200
C	4.65980900	-1.57499300	-2.17177100
C	4.63145700	-0.33006300	-2.73848300
C	3.60632300	0.60990300	-2.39100100
C	3.53087400	1.92313800	-2.91750300
C	2.50304600	2.75760100	-2.53617300
C	1.49834500	2.30148700	-1.63831800
C	0.36147900	3.17989700	-1.28634300
C	-0.95967700	2.70169900	-1.25645400
C	-2.03424600	3.53382600	-0.94262800
C	-1.80127500	4.89007400	-0.65360300
C	-0.48147300	5.38627800	-0.69379500
C	0.57761800	4.54832300	-1.01295000
O	-2.76096000	5.77988500	-0.33793200
C	-4.11708100	5.35581600	-0.27551000
H	1.15876900	-0.69236500	2.73542100
H	-0.23565100	-1.07903200	4.76145600
H	-0.93847800	-5.14922900	3.48424200
H	0.46903900	-4.77629300	1.50817000
H	-2.61755000	-4.30707800	6.61773600
H	-1.34574200	-5.28233100	5.81276600
H	-2.79331100	-4.74771100	4.88824800
H	2.77662900	-4.41412100	0.99708800
H	4.43940700	-3.96006500	-0.79876700
H	5.43856000	-2.29368700	-2.43637700
H	5.38860800	-0.02709700	-3.46498000
H	4.28701500	2.26125600	-3.63004100
H	2.42451900	3.76481900	-2.94618100
H	-1.16061300	1.65530900	-1.49964700
H	-3.04152400	3.11808600	-0.93398800
H	-0.31447900	6.44098100	-0.46481300
H	1.59183700	4.95392700	-1.02611300
H	-4.70484600	6.24075900	0.00312500
H	-4.26208000	4.57116500	0.48707000
H	-4.47015900	4.98168200	-1.25210500
Cl	-1.64711800	-0.48224600	0.95993200

L

E (SMD/B3LYP-D3/def2-SVP) = -1261.9503 au
H (SMD/B3LYP-D3/def2-SVP) = -1261.5276 au
G (SMD/B3LYP-D3/def2-SVP) = -1261.6072 au
E (SMD/B3LYP-D3/def2-TZVP//SMD/B3LYP-D3/def2-SVP) = -1263.3168 au

N	1.66419000	1.13408100	-1.10861000
C	2.64983700	0.27652300	-1.42906700
C	2.74478400	-0.99728600	-0.71656000
N	1.84154900	-1.28190400	0.23937200
C	1.89938600	-2.43250200	0.90631700
C	0.86423400	-2.66448200	1.94805700
C	0.05193500	-1.59456500	2.38644100
C	-0.92323200	-1.77723700	3.35647200
C	-1.13251900	-3.04819500	3.92867500
C	-0.33992500	-4.12726400	3.50165100
C	0.64145400	-3.92597800	2.52696300
O	-2.10264300	-3.13049800	4.86430200
C	-2.36331800	-4.37825100	5.49021600
C	2.91997000	-3.38883400	0.63332500
C	3.85345700	-3.11803700	-0.34418100
C	3.79488700	-1.89884300	-1.06251400
C	4.73827300	-1.56130100	-2.08894700
C	4.64952500	-0.37087200	-2.75490900
C	3.61126800	0.56797600	-2.44222200
C	3.47935300	1.80963800	-3.11021300
C	2.46006000	2.67278500	-2.76834100
C	1.54920000	2.30191800	-1.73732800
C	0.42829900	3.18703200	-1.32338900
C	-0.60345900	2.68189200	-0.51081200
C	-1.67360800	3.47500300	-0.09586700
C	-1.73520000	4.82448900	-0.48834500
C	-0.70880600	5.34773600	-1.29781000
C	0.34854400	4.54343500	-1.70662100
O	-2.72106200	5.68039100	-0.14318400
C	-3.78545800	5.22318100	0.67853000
H	0.20647500	-0.60628000	1.95089700
H	-1.54324900	-0.94443800	3.69674600
H	-0.47992700	-5.12586000	3.91561500
H	1.22773600	-4.79053500	2.21031400
H	-3.17518400	-4.20145400	6.20894000
H	-1.47851300	-4.75501300	6.03279200
H	-2.68937400	-5.14031400	4.76076400
H	2.97857300	-4.32012100	1.19706100
H	4.64829200	-3.83456700	-0.56835500
H	5.52960300	-2.27760800	-2.32530200
H	5.36714300	-0.11244300	-3.53809500
H	4.18775900	2.07092300	-3.90096600
H	2.35139300	3.61993100	-3.29676800
H	-0.56137900	1.63576700	-0.20365400
H	-2.45207200	3.03547200	0.52810900
H	-0.76098400	6.39863200	-1.59139200
H	1.12972000	4.99376500	-2.32173800
H	-4.45456500	6.08193600	0.82580800
H	-3.42186100	4.87796400	1.66229500
H	-4.35084000	4.40615700	0.19682500

X4
E (SMD/B3LYP-D3/def2-SVP) = -3822.574 au
H (SMD/B3LYP-D3/def2-SVP) = -3822.1411 au
G (SMD/B3LYP-D3/def2-SVP) = -3822.2323 au
E (SMD/B3LYP-D3/def2-TZVP//SMD/B3LYP-D3/def2-SVP) = -3824.5015au

Cu	0.15794100	0.42020500	0.52101000
N	1.47914700	1.34291100	-0.82135600
C	2.56805300	0.56203700	-1.05006900
C	2.61580800	-0.74683300	-0.43849100
N	1.60787600	-1.10611500	0.40074900
C	1.63316900	-2.29468900	1.01790900
C	0.61740200	-2.62219300	2.04073300
C	0.19395200	-1.66150700	2.98231500
C	-0.73391600	-1.98197900	3.96175100
C	-1.28332000	-3.27752600	4.02837500
C	-0.86991700	-4.24788000	3.09802400
C	0.07855300	-3.91618700	2.12854900
O	-2.18541500	-3.49390300	5.00802700
C	-2.78143900	-4.77693100	5.13787700
C	2.67219400	-3.22509900	0.73549800

C	3.69422500	-2.88529500	-0.12139000
C	3.70955000	-1.60356400	-0.72363700
C	4.77133000	-1.15402100	-1.57533900
C	4.74632600	0.09971200	-2.11923900
C	3.64502600	0.98286800	-1.87133900
C	3.56028700	2.28442100	-2.42349900
C	2.44177300	3.05301900	-2.19339300
C	1.37310100	2.54539600	-1.40324800
C	0.12137900	3.31977000	-1.27392300
C	-1.13038000	2.68568200	-1.32911200
C	-2.31972900	3.40479100	-1.22342600
C	-2.27736800	4.79968600	-1.05261300
C	-1.02799200	5.45020500	-1.00666300
C	0.14850900	4.72206900	-1.12580900
O	-3.36498000	5.58804000	-0.92939300
C	-4.65764900	4.99825600	-0.94355900
H	0.59834200	-0.64759700	2.94860400
H	-1.05898600	-1.23723700	4.69155600
H	-1.28001800	-5.25735000	3.11839200
H	0.37875300	-4.68072000	1.40802800
H	-3.47119800	-4.71350700	5.99050700
H	-2.02801600	-5.55764600	5.34234900
H	-3.35174500	-5.05418200	4.23408200
H	2.67295100	-4.19035600	1.24248600
H	4.51039600	-3.58359900	-0.32232600
H	5.60334200	-1.83451000	-1.77065200
H	5.55871400	0.45069800	-2.75986900
H	4.37432700	2.65630800	-3.05060600
H	2.34337500	4.03791700	-2.65067700
H	-1.18774000	1.60217000	-1.45428100
H	-3.26802400	2.86950100	-1.26715800
H	-1.00892600	6.53350200	-0.86883400
H	1.10495900	5.24711200	-1.07104900
H	-5.37500100	5.82081700	-0.81896400
H	-4.78578700	4.27986500	-0.11515200
H	-4.86094800	4.48570600	-1.90004900
Cl	-1.78876800	-0.73932200	0.38921300
Cl	-0.33662900	2.11268300	1.95467800

X3

E (SMD/B3LYP-D3/def2-SVP) = -4370.9645 au
H (SMD/B3LYP-D3/def2-SVP) = -4370.5191 au
G (SMD/B3LYP-D3/def2-SVP) = -4370.6208 au
E (SMD/B3LYP-D3/def2-TZVP//SMD/B3LYP-D3/def2-SVP) = -4373.2452 au

Cu	0.06636400	0.26285200	0.22891800
N	1.44132500	1.26337800	-0.91928600
C	2.55757700	0.51338200	-1.12333000
C	2.61725500	-0.79324200	-0.52021700
N	1.59720000	-1.16385200	0.29483300
C	1.64796500	-2.32591600	0.95638800
C	0.64131000	-2.61251900	1.99733100
C	0.23519700	-1.59815200	2.88935900
C	-0.70263900	-1.85473200	3.87760800
C	-1.27475900	-3.13648000	4.00205700
C	-0.86817600	-4.16099600	3.12785900
C	0.08935100	-3.89362300	2.14716500
O	-2.19096300	-3.28749100	4.98134300
C	-2.82256100	-4.54718800	5.15955100
C	2.71176500	-3.23843900	0.71292100
C	3.73997900	-2.89470900	-0.13692800
C	3.74097100	-1.62190400	-0.75954900
C	4.81666200	-1.14224800	-1.57761800
C	4.78140800	0.11711200	-2.10976400
C	3.65497500	0.97688400	-1.88854400
C	3.56983300	2.29579600	-2.39925000
C	2.43789700	3.04429300	-2.16975100
C	1.35055300	2.49297400	-1.43983900
C	0.10116000	3.26019400	-1.27711000
C	-1.14751000	2.65820600	-1.48804000
C	-2.33592400	3.36743000	-1.31464100
C	-2.28812100	4.71385800	-0.91273400
C	-1.03707900	5.33011900	-0.70710700
C	0.13816200	4.61564000	-0.89620300
O	-3.37330400	5.48815900	-0.70276000
C	-4.66978600	4.93049400	-0.86449900

H	0.66756300	-0.59931300	2.80396600
H	-1.01932500	-1.06999200	4.56814500
H	-1.29265500	-5.16229700	3.19916500
H	0.38405000	-4.69620900	1.46656100
H	-3.52524200	-4.42625500	5.99529200
H	-2.09401000	-5.33673500	5.41416200
H	-3.38408600	-4.85191000	4.25903000
H	2.72583900	-4.18916700	1.24699000
H	4.57519600	-3.57830400	-0.30808200
H	5.67187200	-1.79994900	-1.74948400
H	5.60883500	0.49306700	-2.71604100
H	4.39985800	2.70125800	-2.98306700
H	2.34320400	4.05310200	-2.57298300
H	-1.19627500	1.60867500	-1.78920800
H	-3.28674800	2.86348300	-1.48688900
H	-1.01752800	6.37419800	-0.38684500
H	1.09861200	5.09755500	-0.70277000
H	-5.38337300	5.73302400	-0.63326000
H	-4.83929400	4.08736500	-0.17214600
H	-4.83913600	4.58621900	-1.89980200
Cl	-1.77907200	-1.01432800	0.21268900
S	2.19449800	2.44572400	2.27428500
O	2.54189700	3.38098600	1.18846100
O	2.71684000	1.07345800	2.11483000
Cl	-0.49750500	1.93283400	1.77201300

X2

E (SMD/B3LYP-D3/def2-SVP) = -4370.9548 au
H (SMD/B3LYP-D3/def2-SVP) = -4370.509 au
G (SMD/B3LYP-D3/def2-SVP) = -4370.6081 au
E (SMD/B3LYP-D3/def2-TZVP//SMD/B3LYP-D3/def2-SVP) = -4373.2352 au

Cu	0.33937200	0.33447900	0.17906900
N	1.63771600	1.17645700	-1.18028400
C	2.66025400	0.32455100	-1.45754500
C	2.67794400	-0.95631100	-0.79455300
N	1.70697000	-1.22387000	0.12257600
C	1.71581100	-2.37900500	0.80459300
C	0.75834000	-2.60033900	1.90554600
C	0.40642300	-1.55314500	2.78111800
C	-0.48496200	-1.75566100	3.82182700
C	-1.06475700	-3.02286300	4.02426900
C	-0.71863200	-4.08336200	3.16590100
C	0.18996300	-3.86626200	2.12842900
O	-1.93142500	-3.12540400	5.05284400
C	-2.57032500	-4.36784700	5.30952300
C	2.69263900	-3.37038900	0.50438000
C	3.66802200	-3.12858600	-0.43555400
C	3.70548200	-1.88076000	-1.10551600
C	4.72553000	-1.52615700	-2.04803700
C	4.72638300	-0.29836200	-2.65037600
C	3.69440900	0.65506300	-2.36797200
C	3.63774800	1.94048300	-2.96251700
C	2.58741300	2.78348000	-2.67575900
C	1.55612100	2.36785700	-1.78883200
C	0.37945600	3.22585500	-1.54788800
C	-0.91502900	2.68328800	-1.47920700
C	-2.02049000	3.47088800	-1.17154100
C	-1.84858000	4.84362000	-0.92112200
C	-0.56223000	5.40884500	-1.03134300
C	0.53153600	4.61266900	-1.34591500
O	-2.84235100	5.68740200	-0.57836700
C	-4.14072000	5.16343100	-0.32227300
H	0.82853300	-0.55574600	2.65262900
H	-0.75951000	-0.92666400	4.47597400
H	-1.15467300	-5.07422900	3.29178600
H	0.43020900	-4.69830800	1.46235500
H	-3.22534000	-4.20607300	6.17646100
H	-1.84033600	-5.15936400	5.55350200
H	-3.18376000	-4.69491200	4.45179800
H	2.68392600	-4.30635900	1.06321100
H	4.43333700	-3.87807300	-0.65115600
H	5.50729300	-2.25769400	-2.26482000
H	5.50889200	-0.02182400	-3.36059600
H	4.42092200	2.24277400	-3.66198800
H	2.51107900	3.76083900	-3.15323700

H	-1.07081700	1.61576100	-1.65406800
H	-3.00038900	3.00210500	-1.09758600
H	-0.44456600	6.47787900	-0.84077800
H	1.52495500	5.06536600	-1.39144400
H	-4.76030100	6.01514500	-0.00958500
H	-4.11099900	4.41432900	0.48682500
H	-4.58411500	4.71292500	-1.22753100
Cl	-1.64684500	-0.67890300	0.36252200
S	-0.42643800	2.51526100	2.60716100
O	0.02913500	2.05733800	1.23390500
O	-0.24515900	1.48172700	3.64975900
Cl	-2.83311500	2.63927500	2.36495900
